# HIV‐sensitive social protection for vulnerable young women in East and Southern Africa: a systematic review

**DOI:** 10.1002/jia2.25787

**Published:** 2021-09-02

**Authors:** Ran van der Wal, David Loutfi, Quan Nha Hong, Isabelle Vedel, Anne Cockcroft, Mira Johri, Neil Andersson

**Affiliations:** ^1^ Department of Family Medicine McGill University Montreal Quebec Canada; ^2^ EPPI‐Centre UCL Social Research Institute University College London London UK; ^3^ CIET Trust Gaborone Botswana; ^4^ Centre de recherche du Centre Hospitalier de l'Université de Montréal (CRCHUM) Montreal Quebec Canada; ^5^ Département de gestion d’évaluation et de politique de santé École de santé publique de l'Université de Montréal Montreal Quebec Canada; ^6^ Centro de Investigación de Enfermedades Tropicales Universidad Autónoma de Guerrero Acapulco Mexico

**Keywords:** adolescent girls and young women, Africa region, HIV prevention, social support, structural drivers, structural interventions

## Abstract

**Introduction:**

Social protection programmes are considered HIV‐sensitive when addressing risk, vulnerability or impact of HIV infection. Socio‐economic interventions, like livelihood and employability programmes, address HIV vulnerabilities like poverty and gender inequality. We explored the HIV‐sensitivity of socio‐economic interventions for unemployed and out‐of‐school young women aged 15 to 30 years, in East and Southern Africa, a key population for HIV infection.

**Methods:**

We conducted a systematic review using a narrative synthesis method and the Mixed Methods Appraisal Tool for quality appraisal. Interventions of interest were work skills training, microfinance, and employment support. Outcomes of interest were socio‐economic outcomes (income, assets, savings, skills, (self‐) employment) and HIV‐related outcomes (behavioural and biological). We searched published and grey literature (January 2005 to November 2019; English/French) in MEDLINE, Scopus, Web of Science and websites of relevant international organizations.

**Results:**

We screened 3870 titles and abstracts and 188 full‐text papers to retain 18 papers, representing 12 projects. Projects offered different combinations of HIV‐sensitive social protection programmes, complemented with mentors, safe space and training (HIV, reproductive health and gender training). All 12 projects offered work skills training to improve life and business skills. Six offered formal (*n* = 2) or informal (*n* = 5) livelihood training. Eleven projects offered microfinance, including microgrants (*n* = 7), microcredit (*n* = 6) and savings (*n* = 4). One project offered employment support in the form of apprenticeships. In general, microgrants, savings, business and life skills contributed improved socio‐economic and HIV‐related outcomes. Most livelihood training contributed positive socio‐economic outcomes, but only two projects showed improved HIV‐related outcomes. Microcredit contributed little to either outcome. Programmes were effective when (i) sensitive to beneficiaries' age, needs, interests and economic vulnerability; (ii) adapted to local implementation contexts; and (iii) included life skills. Programme delivery through mentorship and safe space increased social capital and may be critical to improve the HIV‐sensitivity of socio‐economic programmes.

**Conclusions:**

A wide variety of livelihood and employability programmes were leveraged to achieve improved socio‐economic and HIV‐related outcomes among unemployed and out‐of‐school young women. To be HIV‐sensitive, programmes should be designed around their interests, needs and vulnerability, adapted to local implementation contexts, and include life skills. Employment support received little attention in this literature.

**Abbreviations**
AGYW: adolescent girls and young women
ELA: Empowerment and Livelihood for Adolescents
HIV: human immunodeficiency virus
HSV‐2: herpes simplex virus‐2
IGA: income‐generating activity
IMAGE: Intervention with Microfinance for AIDS and Gender Equity
IPV: intimate partner violence
SCIP: Strengthening Communities through Integrated Programming
SHAZ!: Shaping the Health of Adolescents in Zimbabwe
SS&CF: Stepping Stones and Creating Futures
TRY: Tap and Reposition Youth
WINGS: Women's Income Generating Support
ZOE: ZOE Orphan Empowerment

## 1 INTRODUCTION

In 2018, East and Southern Africa represented nearly one half of global human immunodeficiency virus (HIV) incident cases [1]. Adolescent girls and young women (AGYW) aged 15 to 25 years accounted for 26%, despite making up 10% of the population [1]. With 6000 new infections per week, their HIV risk is 60% higher than for same‐aged males [1].

Vulnerable young women—defined as unemployed and out‐of‐school, aged 15 to 30 years—are at especially high risk of HIV infection [2, 3]. They may know about this risk [4] but structural drivers of HIV vulnerability like poverty and gender inequality can reduce their ability to act on HIV prevention choices [5]. Absolute poverty is linked with unprotected and transactional sex [6], and unemployment predicts young women's disproportionate HIV burden [2]. Economic vulnerability constrains their ability to negotiate safe sex and makes it harder to leave abusive relationships [7]. Gender inequality at individual level can translate into women's low relationship power; at societal level, harmful hegemonic masculine norms can result in sexual risk taking and violence against women [8]. Out‐of‐school girls do not benefit from the protection implicit in educational attainment [9, 10] or even the lower risk associated with school attendance [11]. HIV infection among female school dropouts is triple that of schoolgirls [3].

In 2005, UNAIDS advanced consensus on combining programmes reducing HIV risk, vulnerability and impact, formalized as ‘combination HIV prevention’ in 2009 [12, 13]. Socio‐economic interventions addressing HIV vulnerabilities like poverty and gender inequality have since been fully endorsed as part of combination HIV prevention [13, 14]. Socio‐economic interventions could improve young women's power to negotiate contraception and pregnancy, delay sexual debut [15], reduce fertility [16], hence influence lifetime earnings and HIV risk. In the context of social protection, socio‐economic interventions aim to enhance income and employability through livelihood and skills development programmes [17]. Such programmes are considered HIV‐sensitive when they also help reduce HIV risk and vulnerability, or mitigate social and economic impacts of the infection [18].

The United Nations Fast‐Track Strategy recommends leveraging HIV‐sensitive social protection to end AIDS by 2030 [19]. Commitment 6 prescribes that 75% of people at risk of, living with, or affected by, HIV benefit from HIV‐sensitive social protection by 2020; Commitment 3 recognizes young women in high‐prevalence countries as key beneficiaries; Commitment 5 states 90% of youth should have the skills, knowledge and capacity to protect themselves from HIV in order to reduce new infections among young women [20]. Beyond income transfers that aim to prevent extreme poverty, like welfare or child grants, the 2018 UNAIDS Guidance Note also encourages using socio‐economic approaches to address structural drivers of HIV vulnerability [21].

Existing systematic reviews on HIV prevention have summarized combined structural interventions [22, 23], income generating [24], microenterprise [25], microcredit [23], and household economic strengthening interventions [26]. No published systematic review has examined HIV‐sensitive social protection interventions for unemployed and out‐of‐school young women, and *how* they were leveraged for HIV prevention. Most existing reviews included men and women of all ages [22, 23, 24, 26]. Some focused on female sex workers [25, 26] or included quantitative studies only [22, 23, 24]. Additionally, despite their premise that socio‐economic empowerment could reduce HIV risk, none assessed socio‐economic outcomes when reporting HIV outcomes.

In the context of HIV prevention, we reviewed published and grey literature on HIV‐sensitive social protection interventions that aim to enhance livelihood and employability among vulnerable young women in East and Southern Africa. We aimed to collate their documented effects on socio‐economic and HIV‐related outcomes and how programmes achieved them.

## 2 METHODS

We conducted a systematic review using the narrative synthesis method by Popay et al. (2006), which supports synthesis of complex interventions with considerable heterogeneity [27]. The method relies on text to synthesize findings from studies using different methods. It involves four steps: (i) developing a theory of change or conceptual framework; (ii) a preliminary synthesis; (iii) exploring of relationships within and across studies; (iv) assessing the robustness of the synthesis [27].

### 2.1 Conceptual framework for HIV‐sensitive social protection

Our theory of change is as follows: socio‐economic and gender inequality increase HIV risk among vulnerable young women, defined as unemployed and out‐of‐school, aged 15 to 30 years, in East and Southern Africa. ‘Cash’ social protection reduced sexual risk behaviours among adolescents in South Africa [28]. HIV‐sensitive social protection interventions that improve livelihood and employability could enhance income and capabilities and similarly enable young women to act on HIV prevention choices. This could reduce sexual risk behaviours and intimate partner violence (IPV) [29], which in turn may reduce incidence of HIV infection.

Interventions of interest are work skills training, microfinance, and employment support. Work skills training include life skills and professional skills training, like business or livelihood training. Livelihood training can be formal (vocational) or informal (income‐generating activity, IGA). Microfinance includes microcredit, savings and microgrants in the form of transfers in cash, in‐kind or productive assets. Employment support can be offered in the form of income transfers for public works, work‐integrated learning like apprenticeships, or job matching services like job placement or career counselling support. Box 1 provides detailed definitions.

**Box 1: Definitions of HIV‐sensitive social protection components**

**Table:** 

HIV‐sensitive social protection components	Definitions
**Work skills training**	
Business training	Entrepreneurial training with goal setting, budgeting, cash flow management, development of business and marketing plans.
Financial literacy	Ranges from basic numeracy to budgeting and accounting. Financial literacy is a combination of awareness, knowledge, skill, attitude and behaviour necessary to make sound financial decisions and ultimately achieve individual financial wellbeing.
Life skills	Set of (non)cognitive skills and abilities that connect knowledge, attitudes and behaviour. Skills that increase self‐ and social awareness; management of self‐ and relationships; stress, coping, communication, negotiation, conflict resolution and self‐efficacy. Higher order life skills include problem solving, responsible decision‐making and critical thinking.
Income‐generating activity (IGA) training	Informal professional skills training for low‐skill self‐employment.
Vocational training	Formal professional skills training at nationally accredited institutions for wage employment.
**Microfinance**	
Microfinance (MFI)	Financial services for the poor who are unable to access formal banking services. It encompasses a range of services including microgrants, microcredit and savings.
Microcredit	Small business loans given to credit groups who use social pressure for loan repayment. Group collateral often consists of mandatory savings. Upon repayment, groups can request larger loans. These small business loans are characterized by short repayment periods and high interest rates.
MFI in‐kind	Material contributions to provide investment capital like kits with products to sell, waiving of training fees or subsidies of materials to support training and IGA.
MFI savings	Services or support that encourage saving to absorb economic shocks or invest in future expenditure: adolescent‐friendly savings accounts; providing a safe place to save; informal revolving group saving schemes.
Productive asset transfers	Transfer of material as investment capital to generate sustainable income. Examples are tools, sewing machines, or agricultural inputs like seed, fertilizer or livestock.
**Employment support**	
Job matching	Services that link individuals with public or private sector employment opportunities, career counselling, job searching and placement support, including support for producing and sharing of curriculum vitae.
Public works	Infrastructure and development projects to transfer income to the poor through (temporary) low‐skill employment. Wages are kept low to target the poorest through self‐selection.
Work‐integrated learning	Occupational opportunities to apply professional training in the real world through observation (internships) or mentoring (apprenticeships).
**Social support**	
Mentorship	Provision of (health) information and (psychosocial) support, training and coaching by often slightly older female mentors who model positive behaviour.
Safe space (social and physical)	Social safe space: regular group meetings that serve as venues for training, information dissemination, critical dialogue, but also for sharing of personal experiences and peer and mentor support. Physical safe space: girls‐only or girl‐friendly clubs where girls benefit from social safe space (meetings) or merely hangout with peers; often with social activities.

**Figure 1:**
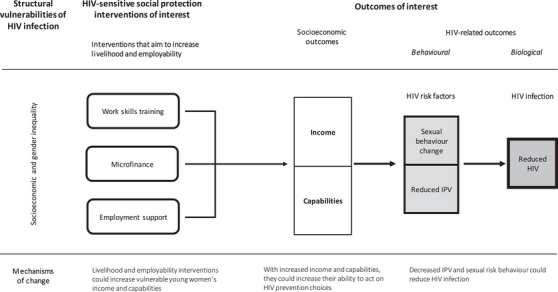
Conceptual framework HIV‐sensitive social protection. The rounded rectangles are intervention components. The arrows represent causal effect. The squares are intended outcomes with more distal outcomes darker.

We consider these interventions HIV‐sensitive when they address both socio‐economic and HIV‐related outcomes. Socio‐economic outcomes include (self‐) employment, income, assets, savings and skills (professional and life skills). HIV‐related outcomes are behavioural (sexual risk behaviour and IPV) and biological: HIV infection, measured as HIV incidence or prevalence, or sexually transmitted infections (Figure 1).

### 2.2 Search strategy

A specialized librarian supported the search strategy based on population, interventions and outcomes of interest described in the theory of change. Studies reporting both socio‐economic and HIV‐related outcomes were included. We used text words and indexing terms to identify published studies in three health and social science databases (MEDLINE, Scopus, and Web of Science Core Collection) and grey literature from websites of the World Bank, International Labour Organization, Centre for Social Protection (IDS), UNAIDS and socialprotection.org. We conducted the search on 28 October 2019 with start date January 2005, when socio‐economic interventions were acknowledged as part of combination HIV prevention [13]. The search was limited by language (English and French) and place (countries in East and Southern Africa with an adult HIV prevalence higher than 2.5%). Study designs included qualitative, quantitative and mixed methods. We checked references of included papers with backward and forward citation tracking. See Box 2 for inclusion and exclusion criteria and Additional file 1 for the search string.

1 **Box 2: Inclusion and exclusion criteria**

**Table:** 

Inclusion criteria (PICOS)	Exclusion criteria
Population: young women aged 15 to 30 years, unemployed and out‐of‐school (baseline dropouts) Intervention of interest: HIV‐sensitive social protection interventions, like work skills training, microfinance, employment support Context: East and Southern African countries with HIV prevalence >2.5% based on UNAIDS Africa ‐ East and Southern: Botswana, Eswatini, Kenya, Lesotho, Malawi, Mozambique, Namibia, South Africa, Uganda, Tanzania, Zambia and Zimbabwe Outcomes of interest: socio‐economic outcomes include wage and (self‐)employment, income, earnings, assets, savings, consumption, and capabilities like business, financial or life skills. HIV‐related outcomes include behavioural outcomes like sexual behaviour and intimate partner violence, and biological outcomes: prevalence and incidence of HIV or sexually transmitted illnesses Study design: quantitative, qualitative, and mixed methods research papers Published in English or French Published from January 2005 to 28 October 2019	Adolescents with mean age lower than 15 years Young women with mean age older than 29 years Female sex workers Studies reporting data not stratified by gender and age Studies that do not report on both socio‐economic and HIV‐related outcomes Preventive or protective social protection like unconditional cash transfers or emergency relief Interventions focused on return to regular education rather than training in support of livelihood and employability Studies reporting the effect of HIV‐sensitive social protection interventions outside the context of HIV prevention, like testing, linkage to care, adherence to treatment, viral suppression Editorials Commentaries Reviews Conference abstracts and proceedings Protocols

### 2.3 Study selection

We removed duplicates with EndNote and screened records in Rayyan QCRI. A two‐stage process involved screening of titles and abstracts, followed by full‐text screening. For review efficiency, we double‐screened a random sample of records until reaching a good interrater agreement [30, 31]. Two reviewers (RW and DL) independently screened a random sample of 10% of titles and abstracts. They resolved disagreements through discussion, which helped clarify selection criteria. As the interrater agreement was good (*k* = 0.85), the first author (RW) screened remaining records [32]. We followed the same process for full‐text screening. During title and abstract screening, we excluded six protocols pertaining to our review topic. In April 2020, we performed forward citation tracking of these protocols, identified associated published papers, and screened them against eligibility criteria. Two reviewers (RW and DL) reviewed all selected papers to confirm the final sample of included studies.

### 2.4 Data extraction, appraisal and synthesis

Following a convergent data‐based synthesis design, we processed included papers with the same synthesis method [33]. One reviewer (RW) extracted data from included papers in two stages. For step 2 of the narrative synthesis (the preliminary synthesis), data extraction followed the population, intervention, context, outcome, study design (PICOS) framework, reported by paper [34]. Several papers reported results for the same project at different stages (pilot and trial) or for different aspects (qualitative and quantitative results). Hence, the second data‐extraction stage involved extraction of detailed implementation data per project (Additional file 2) and programme delivery data (mentorship and safe space) (Additional file 3).

For synthesis step 3, the exploration within and across studies [27, 35], we shifted our focus from projects to intervention components for which we developed two additional tables: (i) the Synthesis Table shows intervention components clustered under work skills training, microfinance and employment support. We report socio‐economic and HIV‐related outcomes and give brief comments on the implementation; (ii) the Summary Table lists *all* intervention components per project, including supporting intervention components, to show projects offered different intervention combinations.

To assess the robustness of included studies (synthesis step 4), two reviewers (RW and DL) independently appraised included papers with the Mixed Methods Appraisal Tool [36]. We rated papers as high, moderate or low quality and contacted authors when missing information. No papers were excluded but ratings were taken into account during the interpretation of findings.

## 3 RESULTS

### 3.1 Study selection

**Figure 2:**
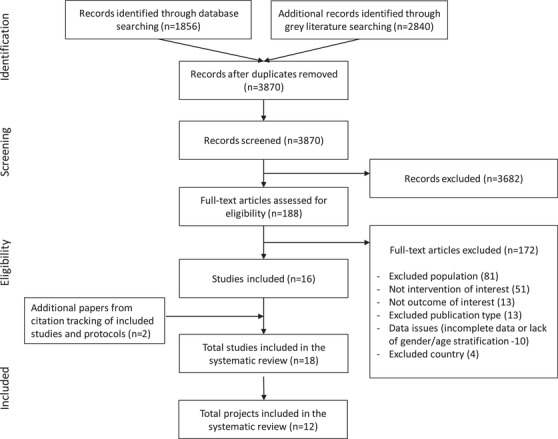
PRISMA flowchart.

The PRISMA flow diagram presents results of the search and selection process (Figure 2) [34]. After removal of duplicate records, we reviewed 3870 titles and abstracts, excluding 3682 in accordance with eligibility criteria (Box 2). Full‐text screening of 188 papers identified 16 papers. Forward citation tracking of relevant protocols identified two additional papers. The resulting 18 papers represented 12 projects. Additional file 4 presents excluded full‐text papers with reasons for exclusion.

### 3.2 Study characteristics

Table 1 shows descriptive characteristics of the 18 included papers. Five papers used qualitative methods, two used mixed methods, six were cluster‐randomized controlled trials (CRCT), one was a randomized controlled trial (RCT) and four were observational, of which one was analytical cross‐sectional; one a clustered non‐equivalent two‐stage cohort trial; one a longitudinal pre–post intervention with matched controls, and one a shortened interrupted time series. The 18 papers represented 12 different projects that included 22,288 participants from eight countries in East and Southern Africa. All had been implemented by nongovernmental organizations. The average intervention duration was 2.8 years, ranging from 18 months to five years. Four projects focused solely on adolescent girls (13 to 19 years) [37, 38, 39, 40, 41, 42, 43, 44]; three on young women 18 years and above [45, 46, 47, 48]; and five on both AGYW [49, 50, 51, 52, 53, 54].

**Table 1:** Descriptive characteristics of HIV‐sensitive social protection studies

Author	Study design	Population	Intervention	Country/year	Outcomes	Score
Austrian 2015	Qualitative Interviews and focus groups	Young women 18–25 years *n* = 128	Asset study Comparison of three groups of young women: Binti comprehensive youth development programme: sexual and reproductive health (SRH), HIV, financial education, leadership and communications skills; cash stipends Vocational training Income generation activities (IGA) without training	Kenya Feb–May 2010	Economic need was key barrier to translate health knowledge into positive behaviour. Most positive outcomes were in Binti group (unwanted sex, pregnancy and education). Social assets facilitated finding employment and mitigating pressure leading to risky sex. Human assets helped to avoid health risks and supported IGA. Economic assets increased negotiation power. Assets interacted and reinforced each other	High
Austrian 2020	Cluster randomized controlled trial (CRCT)	Unmarried, out‐of‐school adolescent girls and young women (AGYW) *n* = 4661 (3515:1146) 15–23 years	**AGEP** (1) Core component: weekly group meetings (safe spaces) with female mentor to receive training on SRH, HIV, life skills, financial education (2) Health vouchers to access free SRH and general wellness services (3) Adolescent‐friendly savings accounts: low fees/opening balance	Zambia 2013–2015 2 years + 2 years after programme end (4 years)	Sustained change on SRH knowledge, self‐efficacy and savings, but intervention did not lead to a combined set of social, health and economic assets. It did reduce transactional sex. Short‐term changes did not lead to long‐term impacts on education or fertility	High
Bandiera 2015	CRCT	In/out‐of‐school adolescent girls 14–20 years Mean (Mn) age 16 *n* = 4800	**ELA‐Uganda** (1) Vocational training: 2‐year training period: general business skills, financial literacy, training for IGA (2) Life skills for SRH/HIV knowledge (menstruation, pregnancy, STI, HIV, family planning) and gender issues (bride price, child marriage, gender‐based violence—GBV) (3) Physical safe space for girls	Uganda 2008–2010 2 years	Girls in intervention areas were twice as likely engaged in IGA (self‐employment). Their earnings and monthly consumption expenditure increased. They reported higher levels of entrepreneurial skills. Fewer girls worried about future jobs. Intervention reduced teenage pregnancy, marriage, sex against their will and increased condom use	Moderate
Bandiera 2018	CRCT	In/out‐of‐school adolescent girls 14–20 years Mn age 16 *n* = 5966 (3964:2002)	**ELA‐Uganda** See under Bandiera 2015 Only physical safe space continued during the 2 years of follow‐up. Girls continued using clubs as safe space without receiving further training	Uganda 2008–2012 4 years	Sustained increases for self‐reported entrepreneurial skills and IGA; not for monthly expenditure. Control over body: significant increase in SRH and HIV knowledge. Trends continued for delayed pregnancy/marriage and reduced unwanted sex. Increased condom use was not sustained	Moderate
Buehren 2017	CRCT	In/out‐of‐school adolescent girls 13–19 years *n* = 5454 (3197 follow‐up)	**ELA‐Tanzania** (1) Vocational training: IGA in local context (2) Life skills for SRH/HIV knowledge (family planning, menstruation, pregnancy, STI, HIV) (3) Physical safe space for girls (4) Community engagement (sensitization of parents and village elders on issues regarding adolescent girls) (5) Microfinance (MFI): offered to older adolescents to engage in IGA; financial literacy and individual business support (planning and management)	Tanzania 2009–2011 2 years	Replication trial of ELA‐Uganda with MFI added to club activities. Despite low MFI uptake, MFI increased club participation, having savings and participation in informal saving groups. There were no other significant effects on outcomes of interest, likely due to lack of implementation fidelity. Unpublished qualitative process evaluation revealed resources constraints, inadequate mentor training and donated safe spaces were not always accessible or safe. Tanzanian girls had different priorities (preferred educational support rather than IGA)	Low
Burke 2019 A	Quasi‐experimental (clustered non‐equivalent two‐stage cohort trial)	Adolescent girls 13–19 years Mn age 15.5 *n* = 885 Interviews: *n* = 266	**SCIP** (1) Business education and business kits of increasing value. Girls had to sell and repay kits. Graduation after third kit; then eligible to receive a bicycle. Some had access to group saving options and linked business capital (2) Facilitator‐led education sessions on gender norms, pregnancy, HIV, unwanted sexual advances, planning goals, assessing values, money, gifts and skills to communicate with adults/partners. To reduce HIV risk and encourage return to school	Mozambique Evaluation 2015 6 months (intervention lasted 5 years)	The 6‐month incidence of intergenerational (1%) and transactional sex (7%) at baseline was so low that measures were dropped No evidence that intervention had impact on girls’ GBV knowledge or school attendance. No accurate measures could be obtained for other outcomes	Moderate
Burke 2019 B	Qualitative Interviews and focus groups Two rounds of data collection (follow‐up)	49 AGYW 13–25 years, 24 head of households, 36 influential males, 12 community leaders	**SCIP** See Burke 2019 A	Mozambique 2015–2016 12 months	Round 1: Most girls reported earning money with business kits. This helped to stay in/return to school, buy necessities, reduce transactional sex Round 2: A quarter of girls continued earning money. Perceptions: Intervention was too short, business kits not sustainable. Most income was used to repay kits. Girls felt shame when having to return to transactional sex. Girls’ GBV knowledge was superficial. Others perceived intervention contributed to reduced early marriage and pregnancies and improved social interaction with girls whose behaviour they perceived as more respectful. Respondents from all groups reported increased community awareness had decreased perpetration of GBV	High
Dunbar 2010	Mixed methods	Out‐of‐school adolescent orphan girls 16–19 years *n* = 50	**SHAZ!‐I** (1) All received life skills (communication and relationship skills) and health education on HIV, and gender. Intervention group also received: (2) Livelihood support: business training, skill building workshops and mentors identified by community (3) MFI (group lending model)	Zimbabwe 2004 6 months	Income and savings significantly increased (likely due to loans), as did girls’ relationship power in nonsexual romantic relationships. There was no change in sexual activity, condom use or future aspirations. There was poor loan repayment, business success, mentoring. Unintended consequences: cross‐border trade increased their vulnerability. MFI is not suitable for adolescent girls	Moderate
Dunbar 2014	RCT	Out‐of‐school HIV‐negative orphan girls 16–19 years Mn age 18 *n* = 315 (158:157)	**SHAZ!‐II** (1) All received life skills (communication and relationship skills) and health education on HIV, and gender (2) All received access to health services: SRH, HIV screening/treatment at every study visit, free condoms, contraception upon request, HIV referral, payment CD4 tests Intervention group also received: (2) Cognitive, material and social support for livelihood: financial literacy, vocational training of choice (nationally accredited), business plan development, microgrant and self‐selected mentors	Zimbabwe 2006–2008	Significant results for reduced food insecurity, having own income, less transactional sex, condom use. There were fewer unintended pregnancies (40%). No statistically significant changes for contraceptive use, HIV and herpes simplex virus‐2 (HSV‐2) incidence. Social support, relationship power and sexual activity were the same across study arms	Moderate
Dunbar 2017	Qualitative Case study interviews, focus groups, process data	See Dunbar 2014	**SHAZ!‐II** See Dunbar 2014	Zimbabwe 2006–2008	Authors explored qualitative (community maps) and process data of Dunbar 2014 study. Effects were likely diluted due to the lack of a true standard of care and control girls using transport money for IGA. Few girls received grant: they moved out/returned to school. Barriers to vocational training were language (not in Shona), length (6 months) and girls' care responsibilities	Low
Erulkar 2005	Longitudinal pre–post with matched controls	Out‐of‐school AGYW 16–22 years *n* = 100 pilot *n* = 326 baseline	**TRY** ‐ Modified group‐based microcredit model in three phases: (1) Pilot (1998–2000): minimalist model; locked up group collateral; social support (2) 2001: loans required adult guarantors. 2002: adult mentors and educational seminars added (health and gender) (3) 2004 assets replace adult guarantors. Young Savers Club (savings only): with passbook	Kenya 1998–2000 (pilot) 2001–2004 (scale‐up)	TRY girls worked more for pay (from 44% to 57%) and significantly improved their income, assets, savings and safe savings (saving at a bank). Older TRY participants (aged 20 and older) had significantly more assets, earnings from paid work, safe savings TRY girls did not have more SRH knowledge but significantly increased their ability to refuse sex (OR 1.7) and insist on condom use (OR 2.86). Overall, the percentage reporting ability to refuse sex decreased, however	Moderate
Erulkar 2006	Qualitative Case study	See Erulkar 2005	**TRY** See Erulkar 2005		There was a mismatch between project design and AGYW. Barriers: inflexible group lending system, long waiting times for credit (1–30 months; average 6 months), inability to access savings locked up as collateral, divisive nature group collateral, lack of credit officers Needs AGYW: acquiring social capital, a safe space to meet, a place to save money. There is a need for a staged programme model with older, bolder AGYW moving on to vocational and business training, work‐integrated learning, MFI	Low
Gibbs 2020	CRCT	Unemployed and out‐of‐school youth 18–30 years Mn age 23.8 (both sexes) *n* = 1351 (677 women; 674 men)	**SS&CF** (1) Stepping Stones: A gender transformative participatory training on HIV and violence prevention programme aimed at more gender‐equitable relationships, communication skills (2) Creating Futures: Participatory learning to critically reflect on livelihood/skill development using existing resources in their environment to develop IGA. Training included business training, psychosocial skills to get/keep jobs, savings, manage debt, cope with shocks	South Africa 2015–2018	At 2‐year endline with ITT: No difference in any of the intimate partner violence (IPV) outcomes, but men's self‐reported physical/severe IPV perpetration significantly reduced For women, significant increase of past‐month earnings (47% increase) and savings (25%) Future studies should try recruiting couples to validate men's self‐reported reductions in IPV perpetration	High
Goodman 2015	Analytical cross‐sectional	Orphan and vulnerable children with 1, 2, 3 years in empowerment programme Median age 18 (Y1 and 2) and 19 (Y3) for girls *n* = 1060	**ZOE** (ZOE Orphan Empowerment) (1) Economic empowerment: Groups received microgrant and decided how to invest it (which entrepreneurial endeavours/training, financial products, or cash); community mentor (2) Sexual behaviour change training and voluntary counselling and testing	Kenya 2012–2014	Although overall programme participation seemed protective against sexual initiation/unprotected sex, material transfers and increased monthly income were largely unassociated with risky sex behaviour. Self‐efficacy was protective for sexual initiation (past 6 months); unprotected sex and multiple sex partners (past year). Improved food consumption increased odds of all outcomes. Differential gender impact: Girls had less but boys more sexually activity	High
Green 2015	CRCT	Young women Mn age 27.3 *n* = 896 Second experiment: *n* = 904 young women +partners. Total n = 1800 (86% female)	**WINGS** ‐ Two interventions to start non‐farm businesses: (1) Economic intervention with business training + seed grant + follow‐up (FU) support for monitoring and advice (2) Economic intervention became control. New intervention (Women Plus—W+): Women and male household member (mostly intimate partner) receive additional 1‐day gender training/communication skills	Uganda 2009–2011 3 years	WINGS: No effect on IPV. Women increased household chores. Doubling of business ownership and income (*p* < 0.01) 16 months after initial grants, moderated by initial quality of relationships. Those suffering IPV increased assets and consumption but not income W+: still no effect on IPV/gender norms, but W+ improved relationship quality and male support for business and household chores. There was little impact on economic outcomes	High
Jewkes 2014	Shortened interrupted time‐series design	Out‐of‐school youth 18–34 years *n* = 232 (*n* = 122 young women and *n* = 110 young men)	**SS&CF** Pilot of trial described above (Gibbs 2020)	South Africa 2012–2013 58 weeks	Significant increases in last month earnings (278%), financially supporting children, receiving child grant, ability to mobilize emergency money and feelings about work situation Significantly improved gender attitudes, decreased sexual IPV, combined physical/sexual IPV in the past 3 months. Problem alcohol drinking increased from 26.6% to 35.5% but quarrelling about alcohol drinking reduced by a half	High
Pettifor 2019	Qualitative Exploratory Narrative timeline interviews	Out‐of‐school AGYW 15–23 years *n* = 40	**WORTH+** AGYW attending at least 10 hours of behaviour change communication were eligible to receive cash transfers (CT $31/3 months) for 18 months. They were offered a place in a small MFI group (savings and loans) and received financial literacy training and mentorship	Tanzania 2017–2018 18 months	AGYW internalized stated aim to develop business, earn money and become less dependent on men. Cash helped to reduce transactional sex among the poorest by meeting basic needs. Business skills enhanced future aspirations and self‐esteem gave AGYW agency to refuse unwanted sex. Social support (family and mentors) enhanced entrepreneurial success	High
Pronyk 2008	Mixed method CRCT + qualitative interviews, focus groups, observation, diaries	Poorest AGYW Nearly all out‐of‐school 14–35 years Mn age 29 *n* = 262 (108:112)	**IMAGE** (Intervention with Microfinance for AIDS and Gender Equity) (1) MFI: Small business loans (2) Sisters for life: Participatory learning sessions about gender roles, IPV, HIV, cultural beliefs, relationships, communication every 2 weeks for about a year	South Africa 2002–2004 2 years	Significant results for increased communications about sex, having gone for testing, reduced unprotected sex with non‐spousal partner HIV incidence was too low (*n* = 8) to examine impact. Qualitative findings revealed increased communication about sex/HIV, especially with children	High

### 3.3 Quality assessment

We rated nine papers as high, six as moderate and three as low quality. Additional file 5 shows the full appraisal of each paper.

### 3.4 HIV‐sensitive social protection interventions and socio‐economic and HIV‐related outcomes

All projects included work skills training, nine offered microfinance, one offered employment support in the form of apprenticeships. None leveraged employment support in the form of public works or job matching. The Synthesis Table of HIV‐sensitive social protection interventions (Table 2) shows intervention components with associated socio‐economic and HIV‐related outcomes and additional implementation information.

**Table 2:** Synthesis Table: Outcomes per HIV‐sensitive social protection component and implementation comments

	Project	Socio‐economic outcomes	HIV‐related outcomes Work skills training	SE	HIV	Comments on implementation
**Life skills**	**All projects**					Lack of description of content, length, quality
	**AGEP**	Life skills training increased self‐efficacy at programme end and 2 years later	Decreased transactional sex, sustained 2 years after programme end. No effect on longer term outcomes (education and fertility)	+	+/−	Self‐efficacy was a life skills outcome, but life skills training was not described
	**Asset**	Teachings in Binti increased self‐esteem	Self‐esteem helped translate knowledge to behaviour change. It helped withstand peer pressure into transactional sex and negotiate condoms	+	+	Life skills training included communication and leadership skills but also reproductive health and HIV information
	**ELA‐Uganda**	Life skills mediated socio‐economic outcomes	Life skills mediated HIV‐related outcomes	+	+	Life skills pertained to management skills: negotiation, conflict resolution, leadership
	**IMAGE**	Improved communication skills, increased self‐confidence, improved bargaining power to negotiate safe sex (life skills)	Testing and communications about sex improved and unprotected sex reduced	+	+	IMAGE offered bi‐weekly gender and HIV training for a year. Authors suggested there was potential for protective effects of increased testing and communications on sexual behaviour change
	**SS&CF**	Critical dialogue and reflection on how to use existing resources in the environment to develop IGA contributed to impressive increases in last month earnings	Gender training effect on combined sexual and physical IPV in pilot; not in trial. No effect on experience of IPV in women. No effects on sexual behaviour change	+	+/−	Harsh environment of informal settlements may be conducive to resorting to violence, which might be difficult to change
	**ZOE Kenya**	Increased self‐efficacy and resilience	Increased self‐efficacy was protective for sexual initiation, unprotected sex, multiple sex partners. Increased resilience was associated with small increases of sexual initiation and multiple sex partners	+	+/−	The paper refers to ‘other life skills training’ on top of entrepreneurial training without specifying content
**Business training**	**Nearly all**					Lack of description of content, length, quality.
	**AGEP**	Modest increase of financial literacy at programme end; not sustained 2 years later. Savings increased and were sustained	Total intervention decreased transactional sex sustainably, without impacting longer term education or fertility outcomes	+	+/−	Intervention component is financial education
	**Asset**	Only Binti girls saved, planned and spent responsibly. They found more ways to increase income	The little money they saved helped young women to not engage in transactional sex	+	+	Intervention component referred to financial education, which was formally integrated in Binti curriculum and helped instil saving behaviour
	**ELA‐Uganda**	Self‐reported entrepreneurial skills up by 8% (ITT); 50% (TOT). This increased self‐employment	The total mix of interventions significantly reduced marriage/cohabitation, teenage pregnancy and sex against their will	+	+	Business skills refer to general business skills and financial literacy (budgeting, financial services, accounting) to support IGA activities. Voluntary participation in development clubs: 21% participation but intense participation (2 or 3/week for 2 years)
**Vocational training**	**Asset**	Young women only receiving vocational training reported saving the least and having the lowest economic assets	They were also perceived as more vulnerable to peer pressure and transactional sex than young women engaged in IGA or graduates from a comprehensive programme (Binti)	−	−	Overwhelming unemployment and high poverty context. Vocational training girls lacked seed capital and professional networks to start IGA. They lacked the positive peer groups and social support Binti girls enjoyed
	**SHAZ!‐II**	In combination with other components reduced food insecurity, having own income	Pilot with small sample size (*n* = 315); underpowered to detect effect on HIV or HSV‐2. Reduced transactional sex, unintended pregnancies; increased condom use	+?	+/−?	SHAZ! offered 6 months vocational training at a nationally accredited institution. Only 63% completed training; 60% received grant. Barriers: Vocational training was in English and at inconvenient times; competing care and household demands
**IGA training**	**SS&CF**	One‐year pilot stage, last month income increased by 278%; during the 2‐year CRCT, income increased by 47%	Decreased combined sexual/physical IPV in pilot. Trial: IPV experience among women did not decrease, but men reported reduced perpetration of IPV. Neither the pilot nor the trial showed significant results for sexual behaviour change	+	−	Participatory learning sessions with wide ranging topics included some training on IGA. SS&CF likely invested the little on IGA training. It is unclear what IGA were pursued and how. Economic results were likely due to critical approach
	**SHAZ!‐I**	More income but this could be caused by loan	No significant change in condom use, sexual activity	+?	−	IGA training involved 4‐day workshops on candle or soap making, tie dye. Girls engaged in risky livelihood strategies, like cross‐border trade. Lack of adequate social support; negative unintended consequences, IGA increased HIV risk
	**ZOE**	Monthly income for girls who were 1 year in the programme was twice that of girls just starting the programme; no difference in income between girls who had been 1 or 2 years in the programme	Monthly salary was not associated with sexual initiation or unprotected sex. Multiple partners even increased with programme participation	+?	−	IGA training included barbering, tailoring, car mechanics. Paper does not describe IGA enough to know how much money and time participants spent on IGA
	**ELA‐Uganda**	Increased (and sustained) engagement with IGA due to self‐employment (72% ‐ ITT). Intervention girls were twice as likely engaged in self‐employment than control girls (6× more likely with TOT analysis). Increased income at programme end was not sustained 2 years later	Teen pregnancy fell by 26%; delayed marriage (58%); sex against their will (44%); all sustained 2 years after project	+	+	ELA offered many types of IGA training: Small‐scale agriculture and livestock rearing, hair dressing, tailoring and small trade. Local entrepreneurs were involved in development and delivery of training curriculum
	**ELA‐Tanzania**	IGA training did not result in any employment, nor in more earnings	No effect on sexual risk behaviours	−	−	Resource constraints affected the delivery of interventions. Donated club spaces were not always accessible and safe; the mentor component suffered from inadequate training, long delays in replacement, and lack of supervision
**Microfinance**						
**Microgrants (cash)**	**Asset**	Stipends gave Binti girls financial assets and instilled saving behaviour	Binti girls perceived improved negotiation power to withstand unwanted sex and pregnancy	+	+	Binti girls received stipends and perceived that economic grounding was key to translate health knowledge into safe sexual behaviours
	**SHAZ!‐II**	In combination with other components, food insecurity reduced	Pilot was underpowered to detect effect on HIV/HSV‐2. Reductions in transactional sex, unintended pregnancies; increased condom use	+	+	After finishing vocational training, girls received microgrant (US $100) to invest in supplies, capital equipment or further training. Only 60% of girls received microgrants because of challenges completing vocational training and business plans
	**WINGS**	Near doubling of monthly earnings and IGA (crop sales, animal rearing, petty trade, retail). Women suffering IPV at baseline only increased assets and food consumption	No effect on IPV; slight but significant increase in marital control	+	−	Increase of 5.8 more household chores. WINGS+ included male partners in a second phase. It increased household and business support, but did not reduce IPV, marital control
	**WORTH+**	Increased agency, self‐esteem, aspirations and future orientation. The better off were able to develop/expand businesses, attend training. Skills enhanced future aspirations like going for job training, buying assets (livestock, land, sewing machine) to secure income beyond cash transfer time period	Grant reduced transactional sex for basic needs. Not having to ask parent or boyfriends for money may also have reduced tension with family and boyfriends and potential violence	+	+	Women internalized the aim to develop IGA to become less dependent on men. Grant size (US $31 every 3 months) was likely too small to reduce transactional sex for other motivations than basic needs. Increased agency, self‐esteem, aspirations and future orientation likely affected HIV risk reduction more
	**ZOE**	Cohort 2 girls earned twice the income compared with Cohort 1 girls. No further increase for Cohort 3 girls	Material inputs and monthly income were unassociated with risky sex. Increased food consumption was associated with increase in sexual risk behaviours	+	−	Value of grant is not reported. Not all participants received the same amount of money. Increase in risky sex could be reverse causation or perhaps girls were perceived as more desirable now they were more food secure. Need for longitudinal data
**Microgrants (in kind)**	**SCIP**	Qualitative findings report that three‐fourths of the girls earned money in the first round, half by the second round and only one‐fourth at time of interview	Unable to determine effect due to design and measurement issues and low‐incidence risky sex behaviours. Many reported reducing transactional sex in round 1 but some re‐engaged in round 2 due to financial need	+?	+/−	Girls discontinued selling business kits due to the lack of diversity of products, low profits, travel, high costs in a context of drought and hunger. Project could be made more sustainable by adding life and professional skills, supplier networks; a longer duration
**Productive assets**	**ELA‐Uganda**	Sustained increased self‐employment	Sustained reduced sexual risk behaviours	+	+	ELA‐Uganda offered US $30 worth of productive assets (seeds, tools, chicks ‐ reported in Buehren 2017)
	**ZOE**	Cohort 2 girls earned twice the income compared with Cohort 1 girls. No further increase for Cohort 3 girls	None of the material inputs were associated with reduced odds of sexual initiation and unprotected sex	+	−	Start‐up kits included sewing machines for tailors, haircutting accessories for hair‐dressers and tools for mechanics. Not all participants received start‐up kits
**Microcredit**	**ELA‐Tanzania**	Credit increased savings; spillover effect from social networks: savings mainly occurred in informal rotating credit/saving schemes	No effect on sexual risk behaviours	+?	−	Microcredit only offered to adolescents to engage in IGA; supported with financial and business training. The MFI component increased interest in overall programme, increasing participation with 6%, but there was a very low uptake of MFI (4%)
	**IMAGE**	No microcredit results reported	Testing and communications about sex improved and unprotected sex reduced		+	IMAGE offered traditional microcredit: Young women received small business loans
	**SCIP**	No outcomes reported for small business loans				Some communities used credit associations to provide group saving and linked business capital (loans), but the main component involved kits
	**SHAZ!‐I**	More income, could be caused by loan	No significant change in condom use, sexual activity	?	−	This was conventional microcredit including group lending, weekly repayment meetings and new loans upon full repayment. Loans were US $51–87; repayment 3–9 months. Interest 30% (vs. 50%–60% commercial lending). At 6 months, only 20% had repaid loan; full loan repayment was 6%. The project adapted to no more collateral/weekly instalments upon timely repayment, but then lacked social pressure to encourage repayment. Traditional MFI loans may not be appropriate for vulnerable adolescent girls
	**TRY**	TRY girls worked more, had more income, savings and saved at safer places; more pronounced for those 20 years and older	They significantly increased ability to refuse sex and insist on condom use and had more liberal gender attitudes	+?	+	TRY offered traditional microcredit with group savings as collateral. Groups consisted of five girls. The two with the best plans receive credit; when fully repaid, the next two, etc. There was little interest in microcredit (54% young women borrowed; 56% had problems with repayment) and faced many barriers
	**WORTH+**	No outcomes reported for loan programme				WORTH+ offered microfinance with individual savings, loans and financial literacy
**Savings**	**AGEP**	Girls increased savings	Transactional sex reduced. Short‐term changes did not lead to long‐term impacts on fertility	+	+/−	Low participation: 25% did not participate at all; 30% participated in half or more sessions. Intervention may not have been meaningful enough. Household poverty may need to be addressed to impact fertility
	**Asset**	Young women in Binti and some with IGA activities managed to save	Savings helped to not having to resort to transactional sex	+	+	Main challenge to saving was not earning enough money, which was especially true for those who took care of others (children or siblings)
	**SCIP**	No outcomes reported for this savings option				Some communities used credit associations to provide group saving and linked business capital (loans)
	**TRY**	Savings increased from 43% to 95%; saving at safer places (at a bank; 42% vs. 24% control); savings remained stable in 2002/2003 but took off in 2004 when voluntary saving scheme was introduced	They significantly increased ability to refuse sex and insist on condom use and had more liberal gender attitudes	+	+	In phase 3 (2004), standalone voluntary savings were added for those enjoying the social aspects of the club/needing a safe place to save. TRY girls were interested in (informal) savings options. Standalone savings and low‐risk income generation activities were valued by (younger) girls
	**WORTH**	Savings improve future orientation: AGYW saved to buy land, assets, livestock	As they can ask savings group for money, they are less tempted to engage in transactional sex	+	+	Savings groups were not described
**Employment support**						
**Public works**	**None**					The lack of research on public works may be due to socio‐cultural norms that view public works as appropriate for men. Public works projects may need to pay more attention to gender and consider additional support to make them sensitive to young women
**Work‐integrated learning**	**SHAZ!‐I**	Distrust between mentors and students; 60% of the girls were satisfied with their mentor. More income ‐ likely due to loan	No significant change in condom use, sexual activity	+?	−	Work‐integrated learning may require more attention to training of mentors, compensation of students, access to and involvement with professional networks and hiring opportunities for employment after the apprentice period
**Job matching**	**None**					The lack of research on employment support may be due to incompatibility of low‐skilled vulnerable young women and limited (higher skills) wage jobs available in contexts with generalized poverty

*Note*: +: positive change; +?: positive change is doubtful; −: no change; +/− both positive and no change; empty cell: no results reported.

#### 3.4.1 Work skills training

All projects offered work skills training. Life and business skills contributed improved socio‐economic and HIV‐related outcomes, which were often sustained after interventions ended. Livelihood training produced mixed results: IGA training improved self‐employment and income, but failed to reduce HIV‐risk behaviours with one exception [37]; standalone vocational training was less suitable for vulnerable young women than more comprehensive interventions.

##### 3.4.1.1 Life skills training

All projects offered life skills training, but few described content and only five reported outcomes of interest [37, 38, 45, 46, 47, 48, 49]. Life skills ranged from skills in communication, negotiation, leadership and conflict‐resolution to higher order skills like problem‐solving and critical thinking. Life skills training increased self‐esteem, self‐confidence, self‐efficacy and aspirations, which helped negotiate condom use, resist transactional sex [47, 49], mediate economic empowerment and unwanted sex [37, 38]. Psychosocial and sexual risk behaviour outcomes were sustained two years after projects ended [37, 38, 49]. ZOE Orphan Empowerment (ZOE) in Kenya showed mixed results. Self‐efficacy was significantly associated with reduced odds of unprotected sex, sexual initiation and concurrency. Increased resilience, however, was associated with small increases of sexual initiation and concurrency [48].

Stepping Stones and Creating Futures (SS&CF) in South Africa took a critical participatory approach to life skills training. Vulnerable young women reflected on skills and resources they could leverage for livelihood and income. Both pilot and full trial reported statistically significant increases in earnings by 278% and 47%, respectively. In the pilot, IPV reduced [46] but the trial showed no effect on women's experience of IPV, although self‐reported male IPV‐perpetration significantly decreased [45]. Neither pilot nor the trial found changes in sexual risk behaviour. The pilot saw young women's drinking problem significantly increased by 33% but quarrelling about alcohol reduced by half. Authors suggested improved communication skills may have de‐escalated conflicts. A similar trend in the trial mid‐way was not sustained at two years [45].

##### 3.4.1.2 Business and financial literacy training

Nearly all projects offered some business or financial training without describing content, duration or level of training. The three projects reporting outcomes offered financial education or general business skills like budgeting and accounting [37, 38, 47, 49]. Financial literacy, self‐efficacy and self‐reported entrepreneurial skills increased. Business skills significantly increased self‐employment [37] and helped young women save, plan and spend responsibly [47, 49]. Projects reported reduced sexual risk behaviour [37, 47, 49]. Improved entrepreneurial skills were sustained two years later [37].

##### 3.4.1.3 Livelihood training (vocational and IGA‐training)

Six projects offered livelihoods training, of which four offered IGA training [37, 38, 39, 45, 46, 48]. Shaping the Health of Adolescents in Zimbabwe (SHAZ!‐I and II) offered both IGA and vocational training [42, 43] and the Asset project in Kenya compared the two types of training [47].

*Formal* vocational training took place at nationally accredited institutions. Asset found vulnerable young women with vocational training at increased socio‐economic and HIV risk compared with peers engaged in IGA or in a comprehensive programme [47]. SHAZ‐II combined vocational training with microgrants, mentors and health services. It found statistically significant results for increased income, food security, condom use, reduced transactional sex and unintended pregnancies [42]. While incidence of HIV (2.3/100 years) and herpes simplex virus‐2 (HSV‐2) infection (4.7/100 years) were high, SHAZ!‐II was not powered to detect statistically significant changes. Only 60% of intervention girls completed vocational training, as they struggled with instruction in English and competing family responsibilities [42, 44].

*Informal* IGA training ranged from candle or soap making, tailoring, hair dressing to small‐scale agriculture or animal rearing. Whereas, income increased in all projects but one, IGA training failed to show impact on sexual risk behaviours. The exception was Empowerment and Livelihood for Adolescents (ELA) in Uganda, which reported increased self‐employment, sustained after two years, and significant reductions of teenage pregnancy, unwanted sex and delayed marriage/cohabitation [37, 38]. The ELA replication trial in Tanzania failed to demonstrate any statistically significant outcome. Resource constraints negatively affected implementation fidelity. The process evaluation identified girls would have preferred supplementary tutoring. Authors suggest this could be linked with school enrolment being higher in Tanzania than in Uganda [39].

Some studies reported unintended outcomes. In Zimbabwe's collapsing economy, some orphan girls started cross‐border trading and faced physical and sexual harm that increased their HIV risk [43]. Increased food consumption in ZOE, Kenya, was associated with increased transactional sex. The authors suggested reverse causality, whereby transactional sex might have increased access to food [48].

#### 3.4.2 Microfinance

All projects offered some form of microfinance, except for SS&CF that encouraged leveraging available resources through capabilities development [45, 46]. Microgrants contributed positive socio‐economic outcomes like increased earnings and savings, but did not always reduce IPV [52] or sexual risk behaviour [48], and impacted the poorest and most vulnerable differently [40, 41, 53]. The single microcredit project showing positive effects judged it suitable for ‘older and bolder’ young women only [50]. Projects offering savings reported improved socio‐economic and HIV‐related outcomes.

##### 3.4.2.1 Microgrants: Cash, in‐kind and productive assets

Seven projects offered microgrants, of which five offered cash grants [42, 47, 48, 52, 53]; two offered productive assets [37, 48]. Strengthening Communities through Integrated Programming (SCIP) in Mozambique offered in‐kind grants in the form of business kits [40, 41]. All projects reported improved socio‐economic outcomes like increased earnings [41, 48, 52], food security [42, 52], savings [47] and self‐employment [37].

Results were mixed for HIV‐related outcomes. Five projects reported reduced sexual risk behaviours [37, 41, 42, 47, 53]. When earnings from business kits halted, some SCIP girls married or re‐engaged in transactional sex out of financial need. SCIP also explored perceptions of heads of households, influential males and community leaders. Many credited the intervention for perceived reductions in early marriage and pregnancy, and more ‘respectful’ behaviour in girls, which could reflect prevailing gender norms. Respondents believed gender training had increased community awareness, reducing intergenerational sex and gender‐based violence (GBV) [41]. Productive assets in ZOE were not associated with sexual behaviour change [48]. In Northern Uganda, microgrants in Women's Income Generating Support (WINGS) had no effect on IPV except for a small but significant increase in marital control. A one‐day gender training session for women and their partners, added in a second phase, had no effect on IPV and economic outcomes, but found significant results for improved communications, quality of relationships and male implication in household chores [52]. Out‐of‐school AGYW in WORTH+ received three‐monthly grants for 18 months. They perceived increased self‐esteem, agency and aspirations. They internalized the goal to develop IGA to reduce transactional sex. Linked to basic needs, only the poorest girls reported reducing transactional sex, whereas the better off developed or expanded businesses. The young women also reported cash grants reduced tensions with family and boyfriends and potential IPV [53].

##### 3.4.2.2 Microcredit

Six projects offered microcredit [37, 38, 39, 40, 41, 43, 50, 51, 53, 54]. Only Tap and Reposition Youth (TRY) in Kenya reported both positive socio‐economic and HIV‐related outcomes, but only 53% of young women took up the offer of microcredit and half had difficulties to repay. The inflexible lending system led to high dropout rates, but young women appreciated the club's safe space and mentors and leveraged their newfound social networks to start informal rotating saving schemes. Those 20 years and older had significantly more assets, income and savings than adolescent girls, and authors concluded that microcredit was appropriate for ‘older and bolder’ young women only [50, 51]. In ELA‐Tanzania, savings similarly increased. Despite low uptake (4%), the offer of microcredit triggered interest in club participation, offering opportunities for informal saving schemes [39].

The Intervention with Microfinance for AIDS and Gender Equity (IMAGE) in our review [54] concerns the subgroup of young women (*n* = 262) from the CRCT in South Africa, which had been ineligible due to women's mean age (41 years) [29]. It combined microcredit with gender training and reported significant results for reduced sexual risk behaviour, improved communications about sex and having gone for testing. Qualitative findings suggested that discussing sex and testing increased young women's self‐confidence and facilitated negotiating safe sex. With eight new HIV infections, the event rate was too low to measure impact on HIV incidence [54].

##### 3.4.2.3 Savings

Of five projects that mentioned savings [40, 41,47, 49–51, 53], SCIP did not report savings outcomes [40, 41]. Four projects reported improved socio‐economic outcomes with increased savings [47, 49, 50, 51], saving at safer places [50, 51], and increased future orientation, as young women saved to buy land or productive assets [53]. Savings enabled young women to refuse sex, insist on condom use [50, 51] and resist transactional sex [47, 49, 53]. In the Adolescent Girls Empowerment Program (AGEP), outcomes did not impact fertility two years after the intervention ended and the most vulnerable girls were more likely married, pregnant or had given birth [49].

#### 3.4.3 Employment support

Only one project, SHAZ!‐I, offered employment support in the form of work‐integrated learning. SHAZ!‐I identified mentors for apprenticeships through community outreach. Hampered by trust issues due to perceived exploitation when mentors lacked time for on‐the‐job training, and perceived laziness of mentees not showing up for work when lacking transport money, SHAZ!‐I changed to mentees choosing their own mentors in SHAZ!‐II. Increased income likely resulted from loans and sexual risk behaviours did not change [42, 43, 44].

#### 3.4.4 Supporting intervention components

All projects offered supporting intervention components that likely contributed to outcomes too (Table 3). Except for WINGS [52], all projects stressed the link with HIV through HIV and sexual and reproductive health education. Some projects facilitated access to healthcare by offering health services [42, 44], health vouchers [49], or encouraged voluntary counselling and testing [48]. Nine projects offered gender training [37, 38, 39, 40, 41, 42, 43, 44, 45, 46, 50, 51, 52, 53, 54].

**Table 3:** Summary Table of all intervention components

		HIV‐sensitive social protection intervention components							Other intervention components					
		Workforce training			Microfinance			Employment support	SRH/HIV/GBV training		Social support			Health
	Project name Country	Life skills	Business and financial training	Livelihood training: IGA & vocational	Grants: cash & in‐kind assets	Credit	Savings	Apprentice ship	Gender training	SRH/HIV training	(Female) Mentor	Social safe space	Physical safe space	Health services
1	AGEP Zambia	x	x				x			x	x	x		x
2	Asset Kenya	x	x	x	x					x	x	x	x	
3	ELA‐Uganda	x	x	x	x				x	x	x	x	x	
4	ELA‐Tanzania	x	x	x		x			x	x	x	x	x	
5	IMAGE South Africa	x				x			x	x		x		
6	SCIP Mozambique	x	x		x	x	x		x	x		x		
7	SHAZ! Zimbabwe	x	x	x	/x	x/		x/	x	x	x	x		/x
8	SS&CF South Africa	x	x	x					x	x	x	x		
9	TRY Kenya	/x	x			x/	/x		/x	/x	/x	x		
10	WINGS Uganda	/x	x		x				/x		x			
11	WORTH+ Tanzania	x	x		x	x	x		x	x	x	x		
12	ZOE Kenya	x	x	x	x					x	x	x		

*Note*: ‘x/’ means that component was offered in an earlier phase of the intervention; and ‘/x’ in the later phase. Abbreviations: IGA, income‐generating activity; SRH, sexual and reproductive health.

#### 3.4.5 Mentorship and safe spaces

All projects instrumentalized mentorship and/or safe space to deliver interventions. Ten projects used mentors who were slightly older young women from the same community [37, 38, 39, 45, 46, 47, 49] or adults [42, 43, 44, 50, 51]. They were positive role models [38, 47] delivering health, gender or life skills training [37, 48, 49, 50, 51], or offering business support [42, 43, 44, 49, 50, 51, 52]. Most mentors received remuneration and mentor training. Mentors helped create social cohesion, boost attendance [50] and were generally appreciated by girls and young women. The lack of a structured framework in SHAZ!‐I led to mistrust between mentors and mentees [43] and inadequate mentor training in ELA Tanzania contributed to null results [39].

Safe space was *social* space, in the form of regular group meetings, or *physical* space, as girls‐only clubs. Except for WINGS [52], all projects offered regular group meetings, although only three referred to it as safe space [37, 38, 39, 49]. Meetings were venues for peer or mentor support, critical dialogue and sharing of experiences. Many offered socialization free from pressures from (older) men and several offered recreational activities. In TRY, these meetings were the only source of social contact and support for girls [50]. Binti Pamoja Centre in Kenya and ELA clubs in Uganda and Tanzania were physical safe spaces [37, 38, 39, 47]. Girls and young women formed new social networks in social and physical safe spaces and leveraged them to start informal rotating saving schemes [39, 50, 51]. They relied on these social networks in times of need, reducing their reliance on transactional sex [47, 53]. In ELA‐Uganda, sustained reductions in sexual risk behaviours at four‐year follow‐up were attributed to mentors and physical safe spaces, as girls continued attending clubs after training activities halted at two years [37]. In contrast, donated club spaces ELA‐Tanzania used were not safe, contributing nonsignificant outcomes [39].

## 4 DISCUSSION

Our systematic review identified 12 HIV‐sensitive social protection projects that aimed to improve socio‐economic and HIV‐related outcomes among unemployed and out‐of‐school young women in East and Southern Africa. All projects offered work skills training, with a majority also offering some type of microfinance. Most projects leveraged mentorship and safe space for programme delivery. Impact on socio‐economic outcomes was mostly positive, albeit modest, but impact on HIV‐related outcomes was less consistent. Employment support was under‐researched.

Our review found insufficient tailoring to participants and local implementation contexts in several interventions. This offers three transferable lessons. First, sensitivity to needs, age, interests, and socio‐economic vulnerability of target populations is essential. Of all interventions, microcredit seemed least responsive to vulnerable young women's needs. Low uptake, as little as 4%, indicates little interest in microcredit among adolescent girls [39, 43, 50, 51]. With few assets and high mobility they are considered credit risks [55]. Loan repayment among microcredit users was low indeed [43, 50, 51]. A recent study found that constraints in savings rather than credit contributed to the inability to sustain increased income after receiving microgrants [56]. Our review shows that young women were eager to save, even starting informal saving schemes in their newfound social networks [39, 50, 51]. These informal saving schemes can help smooth consumption and guard against negative income shocks, but savings will not overcome poverty if all members are poor [57]. Participants' socio‐economic vulnerability also requires attention in programme design. Although microgrants contributed positive socio‐economic and HIV‐related outcomes, grants only reduced transactional sex among the poorest who used it for basic needs, whereas the financially better off managed to develop or expand IGA [53]. The poor are often reluctant to go into debt and lack time and resources to invest in credit groups [58]. In TRY, authors recommended microcredit, but also work‐integrated learning and vocational training for ‘older and bolder’ young women only [50]. Livelihood training should be adapted to young women's social realities. For example, offered at flexible hours with free childcare to account for competing care responsibilities [59]. Vulnerable young women may also need psychosocial support to benefit from interventions. Mentorship and safe space were key to programme delivery but their spillover effect on social capital may indicate another change mechanism. Frequent socialization and sharing of personal experiences created social networks of trust and reciprocity on which young women relied for psychosocial and economic support, enabling some to reduce transactional sex. IMAGE found social networks increased self‐confidence and self‐esteem [60], which facilitated acting on HIV‐prevention choices. Another study found young women belonging to voluntary savings clubs more likely to drink alcohol and engage in casual sex, however [61]. Safe space may therefore require supportive mentors who model positive behaviour.

Second, interventions need to be comprehensive, adapted to local contexts and rely on enabling environments. Although structural, interventions in our review mostly relied on individual behaviour change mechanisms to reduce HIV risk, whereas social and economic environments need to change to address drivers of HIV vulnerability. The Asset study described a context of overwhelming unemployment, sexual harassment while job seeking, and young women lacking professional networks [47]. Zimbabwe's collapsing economy drove girls to risky livelihoods [43], and business kits in SCIP were insufficiently adapted to local context [41]. IGA requires relatively inelastic demand. Vulnerable people prefer steady income flows as they value income most for its capacity to absorb shocks [62]. This requires evaluation of, and interaction with, local markets. In our review, only ELA‐Uganda described demand‐driven IGA with local entrepreneurs delivering training adapted to local markets. It led to high self‐employment and big reductions in sexual risk behaviour, sustained two years after programme end [38].

Vulnerable young women need linking interventions to facilitate their transition into productive livelihoods. The lack of literature on employment support, notably work‐integrated learning and job matching, suggests a lack of ‘linking social capital’, the deliberate connecting of young women with other networks [63]. Interventions could forge private sector links through apprenticeships and coaching like projects did in Latin America [64, 65], Liberia [59] or Uganda [66].

More generally, interventions may require more time and work with other population groups to change gender norms. WINGS added a gender component for men, but the one‐day workshop was insufficient to change gender norms [52]. Interventions could look at how community mobilization efforts in Botswana, South Africa and Uganda changed gender norms through engagement with other population groups [67, 68, 69, 70, 71]. Projects in our review were delivered by NGOs and lasted on average 2.8 years, which might be too short to detect significant improvements in socio‐economic and HIV‐related outcomes, let alone change gender norms. Livelihood and employability interventions may require government involvement and ownership to support a more prolonged, intersectoral approach to HIV‐sensitive social protection and achieve more than the mostly modest outcomes we reported.

Third, the review highlights the pivotal role of life skills. Rarely described in detail although offered by all, few projects aimed to measure life skills outcomes. Life skills training improved self‐efficacy, self‐esteem, sexual negotiation [48, 54], HIV testing [54] and reduced sexual risk behaviours [48, 49]. Self‐confidence and future aspirations facilitated investing in IGA and productive assets [37, 38, 53]. Communication skills may have de‐escalated IPV [45, 46, 52]. SS&CF demonstrated that higher order life skills like critical thinking and dialogue can lead to economic empowerment without any material or financial support [45, 46]. Even when increased income was not associated with reduced sexual risk behaviours, increased self‐efficacy was [48]. Enhanced capabilities can sustain outcomes beyond interventions. Improved self‐efficacy and self‐esteem continued to reduce sexual risk behaviours two years after programmes ended [37, 38, 49], despite not sustaining increased earnings [37, 38].

### 4.1 Updated conceptual framework for HIV‐sensitive social protection

**Figure 3:**
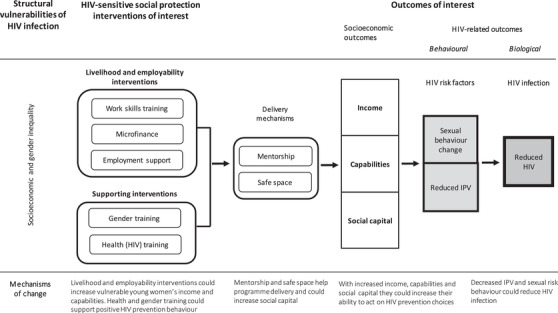
Updated conceptual framework HIV‐sensitive social protection. The rounded rectangles are intervention components. The arrows represent causal effect. The squares are intended outcomes with more distal outcomes darker.

We updated the conceptual framework with findings of this review (Figure 3). For livelihood and employability interventions, we included workforce training, microfinance and employment support. The lack of research on the latter indicates a research gap. As nearly all projects offered additional health and gender training, these have been added as supporting intervention components. We added mentorship and safe space as delivery components along causal pathways to intended outcomes. In addition to income and capabilities, we have added social capital as socio‐economic outcome. Improved income, capabilities and social capital may contribute to reduced IPV and sexual risk behaviour and, ultimately, reduced HIV infection among vulnerable young women.

To our knowledge, this is the first systematic review on HIV‐sensitive social protection interventions for unemployed and out‐of‐school young women reporting both socio‐economic and HIV‐related outcomes. Our use of multiple databases, specialized librarian, two reviewers for quality assessment, detailed data extraction and conceptual grounding contribute to the strengths of this review.

As with any comprehensive intervention with multiple outcomes, it was challenging to attribute specific results to different components. Lack of biomarkers in included studies was another limitation. Including quantitative, qualitative and mixed methods studies provided complementary information that improved understanding of phenomena under study. The narrative synthesis method helped draw out transferable lessons for both impact and change mechanisms.

We recognize a potential selection bias due to independent screening of a proportion of abstracts, titles and full‐text papers by the second reviewer. Other reviews took a similar approach [72, 73] and our selection criteria were clear, reflected by a satisfactory kappa statistic [32].

## 5 CONCLUSIONS

Given intersecting structural drivers of HIV vulnerability, HIV‐sensitive social protection interventions need to be comprehensive and designed around young women's needs, interests and socio‐economic vulnerability. They need to be sensitive to local implementation contexts, to leverage local demand and resources. Microgrants, savings and skills development seem to contribute positive socio‐economic and HIV‐related outcomes, of which life skills are most likely sustained. Microcredit may not be appropriate for unemployed and out‐of‐school girls and young women. The potential of leveraging employment support for HIV‐sensitive socio‐economic programming requires further research. Young women may need psychosocial and professional support to achieve and sustain socio‐economic outcomes from livelihood interventions. This could be instrumentalized in design and delivery through mentorship, safe space and the establishing of linking social capital. To also achieve HIV‐related outcomes, interventions may benefit from government involvement, longer implementation durations and simultaneously work towards an enabling environment in support of more gender‐equal norms.

## COMPETING INTERESTS

The authors declare that they have no competing interests.

## AUTHOR CONTRIBUTIONS

R.W. conceptualized the study; collected, analysed and interpreted the data; appraised quality of included papers; wrote first and subsequent drafts of the paper and revised it after submission. D.L. collected data; reviewed analysis and interpretation; appraised quality of included papers; critically reviewed paper. I.V. and Q.N.H. provided methodological supervision and critically reviewed the paper. A.C., M.J. and N.A. critically reviewed the paper. All authors approved the final version of the manuscript.

## FUNDING

R.W. is supported by CIHR Vanier Canada Graduate Scholarship; Q.N.H. was supported by a FRQS postdoctoral fellowship. The authors thank the Quebec Population Health Research Network (QPHRN) for its contribution to the financing of this publication.

## DISCLAIMER

Funding agencies had no role in the study design, data collection and analysis.

## Supporting information

SUPPORTING INFORMATION

## References

[1] Communities at the Centre. Geneva: UNAIDS ; 2019.

[2] Austin KF, Choi MM, Berndt V. Trading sex for security: unemployment and the unequal HIV burden among young women in developing nations. Int Sociol. 2017;32:343–68.

[3] Stoner MC, Pettifor A, Edwards JK, Aiello AE, Halpern C, Julien A, et al. The effect of school attendance and school dropout on incident HIV and HSV‐2 among young women in rural South Africa enrolled in HPTN 068. AIDS. 2017;31:2127. 28692544 10.1097/QAD.0000000000001584 PMC5599334

[4] Cockcroft A, Kunda JL, Kgakole L, Masisi M, Laetsang D, Ho‐Foster A, et al. Community views of inter‐generational sex: findings from focus groups in Botswana, Namibia and Swaziland. Psychol Health Med. 2010;15:507–14. 20835961 10.1080/13548506.2010.487314

[5] Strive Research Consortium. Addressing the structural drivers of HIV: a strive synthesis. UK: London School of Hygiene & Tropical Medicine; 2019.

[6] Weiser SD, Leiter K, Bangsberg DR, Butler LM, Percy‐de Korte F, Hlanze Z, et al. Food insufficiency is associated with high‐risk sexual behavior among women in Botswana and Swaziland. PLoS Med. 2007;4:e260. 10.1371/journal.pmed.0040260 PMC2039764 17958460

[7] Kim J, Pronyk P, Barnett T, Watts C. Exploring the role of economic empowerment in HIV prevention. AIDS. 2008;22:S57–71. 10.1097/01.aids.0000341777.78876.40 19033756

[8] Jewkes RK, Dunkle K, Nduna M, Shai N. Intimate partner violence, relationship power inequity, and incidence of HIV infection in young women in South Africa: a cohort study. Lancet. 2010;376:41–8. 20557928 10.1016/S0140-6736(10)60548-X

[9] Bärnighausen T, Hosegood V, Timaeus IM, Newell M‐L. The socio‐economic determinants of HIV incidence: evidence from a longitudinal, population‐based study in rural South Africa. AIDS. 2007;21:S29. 10.1097/01.aids.0000300533.59483.95 PMC2847257 18040162

[10] De Neve J‐W, Fink G, Subramanian S, Moyo S, Bor J. Length of secondary schooling and risk of HIV infection in Botswana: evidence from a natural experiment. Lancet Glob Health. 2015;3:e470–7. 26134875 10.1016/S2214-109X(15)00087-X PMC4676715

[11] Jukes M, Simmons S, Bundy D. Education and vulnerability: the role of schools in protecting young women and girls from HIV in southern Africa. AIDS. 2008;22:S41–56. 10.1097/01.aids.0000341776.71253.04 19033754

[12] Gupta GR, Parkhurst JO, Ogden JA, Aggleton P, Mahal A. Structural approaches to HIV prevention. Lancet. 2008;372:764–75. 18687460 10.1016/S0140-6736(08)60887-9

[13] Hankins CA, de Zalduondo BO. Combination prevention: a deeper understanding of effective HIV prevention. AIDS. 2010;24:S70–80. 21042055 10.1097/01.aids.0000390709.04255.fd

[14] Combination HIV Prevention: Tailoring and Coordinating Biomedial, Behavioral and Structural Strategies to Reduce New HIV Infections. Geneva: UNAIDS; 2010.

[15] Sivasankaran A. Work and Women's Marriage, Fertility, and Empowerment: Evidence from Textile Mill Employment in India. Job Market Paper. Cambridge, MA: Harvard University; 2014.

[16] Bloom DE, Canning D, Fink G, Finlay JE. Fertility, female labor force participation, and the demographic dividend. J Econ Growth. 2009;14:79–101.

[17] Devereux S, Sabates‐Wheeler R. Transformative Social Protection. Report No.: 1 85864 844 0. Sussex, UK: Institute of Development Studies; 2004.

[18] HIV and Social Protection. Contract No.: JC2568. Geneva: UNAIDS ; 2014.

[19] United Nations General Assembly . Political Declaration on HIV and AIDS: On the Fast Track to Accelerating the Fight against HIV and to Ending the AIDS Epidemic by 2030. New York: United Nations; 2016.

[20] Fast‐Track Commitments to end AIDS by 2030. Geneva: UNAIDS ; 2016.

[21] Social Protection: a Fast‐Track commitment to end AIDS. Report No.: JC2922. Geneva: UNAIDS ; 2018.

[22] Gibbs A, Willan S, Misselhorn A, Mangoma J. Combined structural interventions for gender equality and livelihood security: a critical review of the evidence from southern and eastern Africa and the implications for young people. J Int AIDS Soc. 2012;15:1–10. 10.7448/IAS.15.3.17362 PMC3499786 22713350

[23] Gibbs A, Jacobson J, Kerr Wilson A. A global comprehensive review of economic interventions to prevent intimate partner violence and HIV risk behaviours. Glob Health Action. 2017;10:1290427. 28467193 10.1080/16549716.2017.1290427 PMC5645712

[24] Kennedy CE, Fonner VA, O'Reilly KR, Sweat MD. A systematic review of income generation interventions, including microfinance and vocational skills training, for HIV prevention. AIDS Care. 2014;26:659–73. 24107189 10.1080/09540121.2013.845287 PMC3943565

[25] Cui RR, Lee R, Thirumurthy H, Muessig KE, Tucker JD. Microenterprise development interventions for sexual risk reduction: a systematic review. AIDS Behav. 2013;17:2864–77. 23963497 10.1007/s10461-013-0582-1 PMC3877769

[26] Swann M. Economic strengthening for HIV prevention and risk reduction: a review of the evidence. AIDS Care. 2018;30:37–84. 29985055 10.1080/09540121.2018.1476665

[27] Popay J, Roberts H, Sowden A, Petticrew M, Arai L, Rodgers M, et al. Guidance on the Conduct of Narrative Synthesis in Systematic Reviews. A Product from the ESRC Methods Programme. Lancaster: Lancaster University; 2006:1018.4643.

[28] Cluver L, Boyes M, Orkin M, Pantelic M, Molwena T, Sherr L. Child‐focused state cash transfers and adolescent risk of HIV infection in South Africa: a propensity‐score‐matched case‐control study. Lancet Glob Health. 2013;1:e362–70. 25104601 10.1016/S2214-109X(13)70115-3

[29] Pronyk PM, Hargreaves JR, Kim JC, Morison LA, Phetla G, Watts C, et al. Effect of a structural intervention for the prevention of intimate‐partner violence and HIV in rural South Africa: a cluster randomised trial. Lancet. 2006;368:1973–83. 17141704 10.1016/S0140-6736(06)69744-4

[30] Booth A, Sutton A, Papaioannou D. Systematic approaches to a successful literature review. Sage; 2016.

[31] Gough D, Oliver S, Thomas J. An introduction to systematic reviews. Sage; 2017.

[32] McHugh ML. Interrater reliability: the kappa statistic. Biochem Med. 2012;22:276–82. PMC3900052 23092060

[33] Hong QN, Pluye P, Bujold M, Wassef M. Convergent and sequential synthesis designs: implications for conducting and reporting systematic reviews of qualitative and quantitative evidence. Syst Rev. 2017;6:61. 28335799 10.1186/s13643-017-0454-2 PMC5364694

[34] Moher D, Liberati A, Tetzlaff J, Altman DG, Group P. Preferred reporting items for systematic reviews and meta‐analyses: the PRISMA statement. PLoS Med. 2009;6:e1000097. 19621072 10.1371/journal.pmed.1000097 PMC2707599

[35] Arai L, Britten N, Popay J, Roberts H, Petticrew M, Rodgers M, et al. Testing methodological developments in the conduct of narrative synthesis: a demonstration review of research on the implementation of smoke alarm interventions. Evid Policy. 2007;3:361–83.

[36] Hong QN, Fàbregues S, Bartlett G, Boardman F, Cargo M, Dagenais P, et al. The Mixed Methods Appraisal Tool (MMAT) version 2018 for information professionals and researchers. Educ Inf. 2018;34:285–91.

[37] Bandiera O, Buehren N, Burgess R, Goldstein M, Gulesci S, Rasul I, et al. Women's Economic Empowerment in Action: Evidence from a Randomized Control Trial in Africa. Contract No.: 187. Geneva: Department of International Labour Organization; 2015.

[38] Bandiera O, Buehren N, Burgess R, Goldstein M, Gulesci S, Rasul I, et al. Women's Empowerment in Action: Evidence from a Randomized Control Trial in Africa. eLibrary: World Bank Group; 2018.

[39] Buehren N, Goldstein M, Gulesci S, Sulaiman M, Yam V. Evaluation of an Adolescent Development Program for Girls in Tanzania Report No.: 7961. e‐Library: The World Bank Group; 2017.

[40] Burke HM, Field S, González‐Calvo L, Eichleay MA, Moon TD. Quasi‐experimental evaluation using confirmatory procedures: a case study of an economic and social empowerment intervention to reduce girls’ vulnerability to HIV in rural Mozambique. Eval Program Plann. 2019;77:101721. 31606720 10.1016/j.evalprogplan.2019.101721

[41] Burke HM, Packer C, González‐Calvo L, Ridgeway K, Lenzi R, Green AF, et al. A longitudinal qualitative evaluation of an economic and social empowerment intervention to reduce girls’ vulnerability to HIV in rural Mozambique. Eval Program Plann. 2019;77:101682. 31369827 10.1016/j.evalprogplan.2019.101682

[42] Dunbar MS, Dufour M‐SK, Lambdin B, Mudekunye‐Mahaka I, Nhamo D, Padian NS. The SHAZ! project: results from a pilot randomized trial of a structural intervention to prevent HIV among adolescent women in Zimbabwe. PLoS One. 2014;9:e113621. 25415455 10.1371/journal.pone.0113621 PMC4240618

[43] Dunbar MS, Maternowska MC, Kang M‐SJ, Laver SM, Mudekunye‐Mahaka I, Padian NS. Findings from SHAZ!: a feasibility study of a microcredit and life‐skills HIV prevention intervention to reduce risk among adolescent female orphans in Zimbabwe. J Prev Interv Community. 2010;38:147–61. 20391061 10.1080/10852351003640849 PMC4578719

[44] Dunbar MS, Mudekunye‐Mahaka I. Empowering adolescent girls and women for improved sexual health in Zimbabwe. In: Kurebwa J, Dodo O, editors. Participation of young people in governance processes in Africa. IGI Global Publisher Online Bookstore; 2017.

[45] Gibbs A, Washington L, Abdelatif N, Chirwa E, Willan S, Shai N, et al. Stepping Stones and Creating Futures intervention to prevent intimate partner violence among young people: cluster randomized controlled trial. J Adolesc Health. 2020;66:323–35. 31784410 10.1016/j.jadohealth.2019.10.004

[46] Jewkes R, Gibbs A, Jama‐Shai N, Willan S, Misselhorn A, Mushinga M, et al. Stepping Stones and Creating Futures intervention: shortened interrupted time series evaluation of a behavioural and structural health promotion and violence prevention intervention for young people in informal settlements in Durban, South Africa. BMC Public Health. 2014;14:1325. 25544716 10.1186/1471-2458-14-1325 PMC4320600

[47] Austrian K, Anderson AD. Barriers and facilitators to health behaviour change and economic activity among slum‐dwelling adolescent girls and young women in Nairobi, Kenya: the role of social, health and economic assets. Sex Educ. 2015;15:64–77.

[48] Goodman ML, Selwyn BJ, Morgan RO, Lloyd LE, Mwongera M, Gitari S, et al. Sexual behavior among young carers in the context of a Kenyan empowerment program combining cash‐transfer, psychosocial support, and entrepreneurship. J Sex Res. 2016;53:331–45. 26421980 10.1080/00224499.2015.1035429

[49] Austrian K, Soler‐Hampejsek E, Behrman JR, Digitale J, Hachonda NJ, Bweupe M, et al. The impact of the Adolescent Girls Empowerment Program (AGEP) on short and long term social, economic, education and fertility outcomes: a cluster randomized controlled trial in Zambia. BMC Public Health. 2020;20:1–15. 32183783 10.1186/s12889-020-08468-0 PMC7079524

[50] Erulkar A, Bruce J, Dondo A, Sebstad J, Matheka JK, Khan AB, et al. Tap and Reposition Youth (TRY): Providing Social Support, Savings, and Microcredit Opportunities for Young Women in Areas with High HIV Prevalence. Contract No.: 23. New York: Population Council; 2006.

[51] Erulkar AS. Evaluation of a Savings and Micro‐Credit Program for Vulnerable Youth Women in Nairobi. New York: Population Council; 2005. p. 34.

[52] Green EP, Blattman C, Jamison J, Annan J. Women's entrepreneurship and intimate partner violence: a cluster randomized trial of microenterprise assistance and partner participation in post‐conflict Uganda (SSM‐D‐14‐01580R1). Soc Sci Med. 2015;133:177–88. 25875324 10.1016/j.socscimed.2015.03.042

[53] Pettifor A, Wamoyi J, Balvanz P, Gichane MW, Maman S. Cash plus: exploring the mechanisms through which a cash transfer plus financial education programme in Tanzania reduced HIV risk for adolescent girls and young women. J Int AIDS Soc. 2019;22:e25316. 31328425 10.1002/jia2.25316 PMC6643075

[54] Pronyk PM, Kim JC, Abramsky T, Phetla G, Hargreaves JR, Morison LA, et al. A combined microfinance and training intervention can reduce HIV risk behaviour in young female participants. AIDS. 2008;22:1659–65. 18670227 10.1097/QAD.0b013e328307a040

[55] Kim JC, Watts CH, Hargreaves JR, Ndhlovu LX, Phetla G, Morison LA, et al. Understanding the impact of a microfinance‐based intervention on women's empowerment and the reduction of intimate partner violence in South Africa. Am J Public Health. 2007;97:1794–802. 17761566 10.2105/AJPH.2006.095521 PMC1994170

[56] Brudevold‐Newman A, Honorati M, Jakiela P, Ozier O. A Firm of One's Own: Experimental Evidence on Credit Constraints and Occupational Choice. The World Bank; 2017.

[57] Larson BA, Wambua N, Masila J, Wangai S, Rohr J, Brooks M, et al. Exploring impacts of multi‐year, community‐based care programs for orphans and vulnerable children: a case study from Kenya. AIDS Care. 2013;25:S40–5. 23745629 10.1080/09540121.2012.729807 PMC4003573

[58] Hulme D. Is microdebt good for poor people? A note on the dark side of microfinance. Small Enterp Dev. 2000;11:26–8.

[59] Adoho F, Chakravarty S, Korkoyah DT, Lundberg M, Tasneem A. The impact of an adolescent girls employment program: the EPAG project in Liberia. The World Bank; 2014.

[60] Pronyk PM, Harpham T, Busza J, Phetla G, Morison LA, Hargreaves JR, et al. Can social capital be intentionally generated? A randomized trial from rural South Africa. Soc Sci Med. 2008;67:1559–70. 18771833 10.1016/j.socscimed.2008.07.022

[61] Campbell C, Williams B, Gilgen D. Is social capital a useful conceptual tool for exploring community level influences on HIV infection? An exploratory case study from South Africa. AIDS Care. 2002;14:41–54. 11798404 10.1080/09540120220097928

[62] Mutenje MJ, Nyakudya IW, Katsinde C, Chikuvire TJ. Sustainable income‐generating projects for HIV‐affected households in Zimbabwe: evidence from two high‐density suburbs. Afr J AIDS Res. 2007;6:9–15. 25875340 10.2989/16085900709490394

[63] Szreter S, Woolcock M. Health by association? Social capital, social theory, and the political economy of public health. Int J Epidemiol. 2004;33:650–67. 15282219 10.1093/ije/dyh013

[64] Attanasio O, Kugler A, Meghir C. Training Disadvantaged Youth in Latin America: Evidence from a Randomized Trial. Report No.: 0898‐2937; Contract No.: 13931. National Bureau of Economic Research; 2008.

[65] Díaz JJ, Jaramillo M. An evaluation of the Peruvian ‘youth labor training program’–Projoven. Working Paper WP‐10/6. Washington, DC: Office of Evaluation and Oversight, Inter American Development Bank; 2006.

[66] Bukuluki PM, Kamya S, Kasirye R, Nabulya A. Facilitating the transition of adolescents and emerging adults from care into employment in Kampala, Uganda: a case study of Uganda youth development link. Emerg Adulthood. 2020;8:35–44.

[67] Abramsky T, Devries K, Kiss L, Nakuti J, Kyegombe N, Starmann E, et al. Findings from the SASA! Study: a cluster randomized controlled trial to assess the impact of a community mobilization intervention to prevent violence against women and reduce HIV risk in Kampala, Uganda. BMC Med. 2014;12:122. 25248996 10.1186/s12916-014-0122-5 PMC4243194

[68] Cockcroft A, Kgakole L, Marokoane N, Andersson N. A role for traditional doctors in health promotion: experience from a trial of HIV prevention in Botswana. Glob Health Promot. 2020;27:114–6. 10.1177/1757975918785563 30284942

[69] Cockcroft A, Marokoane N, Kgakole L, Mhati P, Tswetla N, Sebilo I, et al. Acceptability and challenges of introducing an educational audio‐drama about gender violence and HIV prevention into schools in Botswana: an implementation review. AIDS Care. 2019;31:1397–402. 30909721 10.1080/09540121.2019.1595521

[70] Pettifor A, Lippman SA, Gottert A, Suchindran CM, Selin A, Peacock D, et al. Community mobilization to modify harmful gender norms and reduce HIV risk: results from a community cluster randomized trial in South Africa. J Int AIDS Soc. 2018;21:e25134. 29972287 10.1002/jia2.25134 PMC6058206

[71] Wagman JA, Gray RH, Campbell JC, Thoma M, Ndyanabo A, Ssekasanvu J, et al. Effectiveness of an integrated intimate partner violence and HIV prevention intervention in Rakai, Uganda: analysis of an intervention in an existing cluster randomised cohort. Lancet Glob Health. 2015;3:e23–33. 25539966 10.1016/S2214-109X(14)70344-4 PMC4370228

[72] Mohsin‐Shaikh S, Furniss D, Blandford A, McLeod M, Ma T, Beykloo MY, et al. The impact of electronic prescribing systems on healthcare professionals’ working practices in the hospital setting: a systematic review and narrative synthesis. BMC Health Serv Res. 2019;19:742. 31640689 10.1186/s12913-019-4554-7 PMC6806498

[73] O'Brien N, Hong QN, Law S, Massoud S, Carter A, Kaida A, et al. Health system features that enhance access to comprehensive primary care for women living with HIV in high‐income settings: a systematic mixed studies review. AIDS Patient Care STDS. 2018;32:129–48. 29630850 10.1089/apc.2017.0305

