# Supplementary material for: HIV‐sensitive social protection for vulnerable young women in East and Southern Africa: a systematic review
Source: J Int AIDS Soc. 2021 Sep 2;24(9):e25787. doi: 10.1002/jia2.25787 (PMC8412122; doi:10.1002/jia2.25787)
Supplement: Supplementary file 1 — SUPPORTING INFORMATION [file JIA2-24-e25787-s001.docx]

**Additional File 1. Search string**

**HIV-sensitive social protection for vulnerable young women in East and Southern Africa: a systematic mixed studies review**

**Database search 28/10/2019**

**Ovid MEDLINE:**

(financing, government/ or public assistance/ or financial support/ or training support/ or vocational guidance/ or ("social protection" OR "economic empowerment" OR apprenticeship* OR internship* or ((develop* OR build* OR technical OR financial OR negotiation OR soft) adj1 skill*) OR ((job OR skill* OR vocational OR entrepreneur*) adj3 (train* OR educat*)) OR "life skill*" OR "communication skill*" OR "income generation" OR "cash incentive*" OR "in kind" OR lotter* OR "starting capital" OR "economic incentiv*" OR "financial* incentiv*" OR "micro enterprise*" OR microenterprise* OR "micro credit*" OR microcredit* OR microfinance* OR "micro finance*" OR loan* OR "school fee*" OR "tuition payment*" OR "bursar*" OR "school grant*" OR "school facilitation" OR "education* facilitation" OR "youth training" OR "workforce education" OR "workforce readiness" OR "economic strengthening" OR "food for work" OR "cash for work" OR (transfer* adj3 (cash OR asset* OR income)) OR livelihood* OR voucher* OR allowance* OR ((career or employment or job or vocation*) adj3 (counsel?ing or guidance or placement* or support))).mp.)

AND

(exp sexual behavior/ or condoms/ or exp hiv infections/ or exp herpes simplex/ or intimate partner violence/ or student dropouts/ or employment/ or unemployment/ or self concept/ or self efficacy/ or risk-taking/ or exp educational status/ or (sex* or abstinence OR "HIV" or "risk taking" or herpes or HSV* or condom* or ((concurren* or new or number or multiple) adj3 partner*) or "intimate partner violence" OR dropout* OR drop out* OR "out of school" OR "return to school" OR "school attendance" OR employment OR unemploy* OR "self confidence" OR wellbeing OR "well being" OR "self- esteem" or (education* adj1 (status OR attain* or achiev*))).mp.)

AND

(Africa, Eastern/ or Africa, Southern/ or Africa South of the Sahara/ or (Africa OR Botswana OR Eswatini OR Kenya OR Lesotho OR Malawi OR Mozambique OR Namibia OR Swaziland OR Uganda OR Tanzania OR Zambia OR Zimbabwe).mp.)

AND

(((adolescent/ or young adult/) and female/) or (((young OR vulnerable OR marginali#ed) AND wom#n) OR girl* OR adolescen*).ti,ab,kf.)

AND

(English or French).lg.

**SCOPUS:**

TITLE-ABS-KEY ( "social protection" OR "economic empowerment" OR apprenticeship* OR internship* OR ( ( develop* OR build* OR technical OR financial OR negotiation OR soft ) W/1 skill* ) OR ( ( job OR skill* OR vocational OR entrepreneur* ) W/3 ( train* OR educat* ) ) OR "life skill*" OR "communication skill*" OR "income generation" OR "income-generation" OR "cash incentive*" OR "in kind" OR lotter* OR "starting capital" OR "economic incentiv*" OR "financial* incentiv*" OR "micro enterprise*" OR microenterprise* OR "micro-enterprise*" OR "micro credit*" OR microcredit* OR "micro-credit" OR microfinanc* OR "micro financ*" OR "micro-financ*" OR loan* OR saving* OR "school fee*" OR "tuition payment*" OR bursar* OR "school grant*" OR "school facilitation" OR "education* facilitation" OR "youth training" OR "workforce education" OR "workforce readiness" OR "economic strengthening" OR "food for work" OR "food-for-work" OR "cash for work" OR "cash-for-work" OR ( transfer* W/3 ( cash OR asset* OR income ) ) OR livelihood* OR voucher* OR allowance* OR "employment support" OR ( ( career OR employment OR job OR vocation* ) W/3 ( counsel?ing OR guidance OR placement* OR support ) ) ) AND TITLE-ABS-KEY ( sex* OR abstinence OR "HIV" OR "risk taking" OR herpes OR hsv* OR condom* OR ( ( concurren* OR new OR number OR multiple ) W/3 partner* ) OR "intimate partner violence" OR dropout* OR "drop out*" OR "out of school" OR "return to school" OR "school attendance" OR employment OR unemploy* OR "self confidence" OR "self-confidence" OR wellbeing OR "well being" OR "well-being" OR "self esteem" OR "self-esteem" OR ( education* W/1 ( status OR attain* OR achiev* ) ) )

AND TITLE-ABS-KEY ( africa OR botswana OR eswatini OR kenya OR lesotho OR malawi OR mozambique OR namibia OR swaziland OR uganda OR tanzania OR zambia OR zimbabwe )

AND TITLE-ABS-KEY ( ( ( young OR vulnerable OR marginali?ed OR unemploy* ) AND wom?n ) OR girl* OR adolescen* )

AND LANGUAGE ( english OR french ) AND ( LIMIT-TO ( LANGUAGE , "English" ) OR LIMIT-TO ( LANGUAGE , "French" ) )

AND ( LIMIT-TO ( PUBYEAR , 2019 ) OR LIMIT-TO ( PUBYEAR , 2018 ) OR LIMIT-TO ( PUBYEAR , 2017 ) OR LIMIT-TO ( PUBYEAR , 2016 ) OR LIMIT-TO ( PUBYEAR , 2015 ) OR LIMIT-TO ( PUBYEAR , 2014 ) OR LIMIT-TO ( PUBYEAR , 2013 ) OR LIMIT-TO ( PUBYEAR , 2012 ) OR LIMIT-TO ( PUBYEAR , 2011 ) OR LIMIT-TO ( PUBYEAR , 2010 ) OR LIMIT-TO ( PUBYEAR , 2009 ) OR LIMIT-TO ( PUBYEAR , 2008 ) OR LIMIT-TO ( PUBYEAR , 2007 ) OR LIMIT-TO ( PUBYEAR , 2006 ) OR LIMIT-TO ( PUBYEAR , 2005 ) ) AND ( EXCLUDE ( DOCTYPE , "re" ) OR EXCLUDE ( DOCTYPE , "cp" ) OR EXCLUDE ( DOCTYPE , "bk" ) OR EXCLUDE ( DOCTYPE , "ed" ) OR EXCLUDE ( DOCTYPE , "le" ) OR EXCLUDE ( DOCTYPE , "no" ) )

**Web of Science Core Collection:**

TS=("social protection" OR "economic empowerment" OR apprenticeship* OR internship* OR ((develop* OR build* OR technical OR financial OR negotiation OR soft) NEAR/1 skill*) OR ((job OR skill* OR vocational OR entrepreneur*) NEAR/3 (train* OR educat*)) OR "life skill*" OR "communication skill*" OR "income generation" OR "cash incentive*" OR "in kind" OR lotter* OR “starting capital” OR "economic incentiv*" OR "financial* incentiv*" OR "micro enterprise*" OR microenterprise* OR "micro credit*" OR microcredit* OR microfinanc* OR "micro financ*" OR loan* OR "school fee*" OR "tuition payment*" OR bursar* OR "school grant*" OR "school facilitation" OR "education* facilitation" OR "youth training" OR "workforce education" OR "workforce readiness" OR "economic strengthening" OR "food for work" OR "cash for work" OR (transfer* NEAR/3 (cash OR asset* OR income)) OR livelihood* OR voucher* OR allowance* OR “employment support” OR ((career OR employment OR job OR vocation*) NEAR/3 (counsel?ing OR guidance OR placement* OR support))) AND TS=(sex* OR abstinence OR "HIV" OR "risk taking" OR herpes OR HSV* OR condom* OR ((concurren* OR new OR number OR multiple) NEAR/3 partner*) OR “intimate partner violence” OR dropout* OR "drop out*" OR "out of school" OR "return to school" OR "school attendance" OR employment OR unemploy* OR “self confidence” OR wellbeing OR “well being” OR "self esteem" OR (education* NEAR/1 (status OR attain* OR achiev*))) AND TS=(Africa OR Botswana OR Eswatini OR Kenya OR Lesotho OR Malawi OR Mozambique OR Namibia OR Swaziland OR Uganda OR Tanzania OR Zambia OR Zimbabwe) AND TS= (((young OR vulnerable OR marginali?ed) AND wom?n) OR girl* OR adolescen*)

Limits: English or French

**Additional file 2. Detailed implementation data extraction sheets per project**

1. **AGEP**

| **Programme/Project Name** | The Adolescent Girls Empowerment Program (**AGEP**) |
| --- | --- |
| **Paper(s)** | Austrian K, Soler-Hampejsek E, Behrman JR, Digitale J, Hachonda NJ, Bweupe M, et al. The impact of the Adolescent Girls Empowerment Program (AGEP) on short and long term social, economic, education and fertility outcomes: a cluster randomized controlled trial in Zambia. BMC public health. 2020;20(1):1-15 |
| **Implementing Partners** | Population Council, Young Women’s Christian Association, Making Cents International, the National Savings and Credit Bank of Zambia, Ministry of health, Government of the Republic of Zambia. Several Nairobi slum vocational training programmes |
| **WHAT?**  **Stated aim**  **Type of HIV-sensitive social protection** | Aim: to improve social, health and economic assets in order to work towards improving longer term outcomes like sexual behaviour change, early marriage, pregnancy and education.  Comparison effect three different interventions (1 core + 2 add-ons)  Livelihood or employability aspect: life skills, financial education, ado friendly savings account  HIV-link: Sexual and Reproductive Health (SRH)/HIV knowledge, sexual behaviour, early marriage, pregnancy, gender equity norms, acceptability sexual and gender-based violence (SGBV) |
| **Design** | Cluster randomized controlled trial (CRCT) with longitudinal observations |
| **WHEN?** | Baseline July 2013-Feb 2014  Endline (July 2015-Jan 2016 = 2 years)  Follow-up: 2 years after programme end (July-Dec 2017 = 4 years). |
| **WHO?**  **Sample description Inclusion criteria** | Never-married, out of school adolescent girls and young women (AGYW); 10-14 & 15-23 years old; n= 4661 (3515 intervention; 1146 control);  clusters: 120 intervention; 40 control  4 Groups: (1) core intervention; (2) core + health; (3) core + health + savings; (4) control  Aim: 40 clusters per study arm; 20 participants per cluster; 10 per age cohort (n=3200)  From a list of 2000 most vulnerable AGYW, random individual sampling (over sampling 15-23-year-old). |
| **WHERE?**  **Country & Context** | **Zambia** –10 sites: 5 rural; 5 urban; provinces purposively selected; within this stratification, sites randomly selected. |
| **HOW?**  **Description HIV-sensitive social protection intervention** | **1**. Core component: weekly group meetings (safe spaces) with female mentor; segmented by age and marital status; curriculum: SRH, HIV, life skills, financial education  **2**. health vouchers to access free SRH/general wellness services  **3**. savings account; adolescent-friendly: low fees/opening balance, allowing transactions minors (partnership with National Savings and Credit Bank Zambia). |
| **Outcomes of interest** | Short-term/mediators: (1) **social** assets (self-efficacy, safe space, attitudes gender/SGBV; (2) **economic** assets (financial literacy, savings behaviour); (3) **health** assets (knowledge HIV/SRH); (4) sexual behaviour change (condom/transactional sex) for AGYW 15-23 years.  Longer-term: educational attainment (completion primary school=grade 7/grade 9)  Fertility (ever had sex, been pregnant/married, given birth) + age (sexual debut, first birth, marriage). |
| **Outcomes** | Sustained change on SRH knowledge, self-efficacy and savings, but intervention did not lead to a combined set of social, health and economic assets. Significant reduction of transactional sex (sustained 2 years after programme end). Short term changes did not lead to long term impacts on education or fertility.  Few meaningful differences between subgroups: in older AGYW higher self-efficacy, non-acceptability IPV (marginally significant at p<0.1); urban girls more likely to have initiated sex; most vulnerable girls more likely married/pregnant/have given birth. |
| **Comments** | Low participation: 25% did not participate at all; 30% participated in half or more sessions; analysis with treatment on the treated (TOT) also failed to show meaningful change, except for those who participated in 52 sessions: TOT estimates were 3 times as large.  Intervention might not have been meaningful enough for girls and their families. Perhaps household poverty needs to be addressed to impact education and fertility, for example cash transfers.  Need to look at social ecology: positive changes pertain to factors over which girls may have some control; lack of impact may indicate choice disability. Only targeting AGYW in a wider context of high SGBV prevalence/acceptance makes it difficult to change norms. Beliefs mentors could have played a role.  Conclusion: to impact longer term education/fertility outcomes, interventions targeting only girls may be insufficient. |

1. **Asset**

| **Programme/Project Name** | Barriers & Facilitators health behaviour change & economic activity AGYW Nairobi (**Asset**) |
| --- | --- |
| **Paper(s)** | Austrian K, Anderson AD. Barriers and facilitators to health behaviour change and economic activity among slum-dwelling adolescent girls and young women in Nairobi, Kenya: The role of social, health and economic assets. Sex Education. 2015;15(1):64-77. |
| **Implementing Partners** | Binti Pamoja Centre (Binti)  Several Nairobi slum vocational training programmes |
| **WHAT?**  **Stated aim**  **Type of HIV-sensitive social protection** | *(1) What factors facilitate and/or hinder the translation of health knowledge into behaviour change for young women living in urban slum areas of Nairobi?*  *(2) How do different assets work together to prepare and help facilitate a young woman’s transition into economic activity in urban slum areas of Nairobi?*  To explore perceptions of AGYW regarding multidimensional barriers and facilitators to health behaviour change and transition to economic activity within the context of social, health and economic assets; to assess how programmes can leverage these different sets of assets to improve health and economic outcomes  Livelihood or employability aspect: business training, vocational training, income generation  HIV-link: safe sex, teenage pregnancy, abortion, early marriage |
| **Design** | Qualitative (QL): in-depth interviews (IDI) & focus group discussions (FGD) |
| **WHEN?** | Feb-May 2010 |
| **WHO?**  **Sample description Inclusion criteria** | 128 young women, 18-25-year-old.  63 IDI (24 Binti; 19 vocational training; 20 no programme participation)  11 FGD with 65 participants (2 Binti; 5 vocational training; 4 no programme participation) |
| **WHERE?**  **Country & Context** | **Kenya** –Kibera and various other slums in Nairobi |
| **HOW?**  **Description HIV-sensitive social protection intervention** | Comparison of 3 groups of young women with different sets of assets.  **Group 1**: Binti Pamoja Centre in Kibera: youth development programme: SRH, HIV, financial education, leadership & communications skills; graduates receive stipends to run additional girl groups in their community. They have very strong social and human assets; medium/high economic assets (stipends + savings behaviour).  **Group 2**: young women from 5 vocational training programmes –medium social assets, medium/high human assets (vocational skill) and medium/low economic assets (irregular income earning opportunities).  **Group 3**: economically active young women (some income generation activity (IGA)) but without having participated in either intervention 1 or 2. They have low social assets (but strong work ethic), low human assets (little health knowledge, formal education, financial literacy); medium/low economic assets because income sources are small and irregular. |
| **Outcomes of interest** | Barriers & facilitators of: (1) health behaviour change; (2) engaging in economic activity |
| **Outcomes** | **Health behaviour change**: major factors were economic factors and social influences.  *Barriers*: Unprotected sex was a calculated decision because YW needed men’s financial support. Parents expect girls to either contribute financially or move out, hence early marriage to obtain bride price or so that girl becomes financial responsibility husband and his family. Vocational training girls were least likely to deflect peer pressure to appear to be taken care of by boyfriend/sugar daddy. Binti alumni had strong social support; both Binti and IGA girls had high self-esteem.  *Facilitators*: (1) SRH & HIV information –although Binti girls had information, socioeconomic barriers prevented them from acting on them. (2) Binti girls had the obligation to be positive role models and access to (3) girls’ spaces. (4) Self-esteem for those with training. (5) Earning money to be able to contribute and independent from men.  **Transition into economic activity**: A combination of social, human and economic assets are likely necessary to overcome challenges and transit into economic activity.  *Barriers*: all training/skills development do not change (1) context of overwhelming unemployment; (2) sexual harassment while job seeking (bosses expect sexual favours); (3) lack of capital (seed grant/loan) for IGA; (4) lack of professional networks/work experience/social connections.  *Facilitators*: survival is main motivating factor to earn money; having to provide for children. (1) social assets –financial independence begets respect among family, boyfriends, community; (3) human assets –only Binti girls received financial education, which helped them (a) save; (b) plan & spend responsibly; (c) be more creative about IGA. (4) economic assets –mainly saving (Binti & IGA girls); vocational training girls desired to save but made too little/had too many expenses (especially young women with kids). |
| **Comments** | Economic need is a key barrier to translating health knowledge into positive behaviour. Binti girls had most positive health outcomes re unwanted sex, pregnancy & education. Social assets helped to find employment and mitigate pressure leading to risky sex; human assets protected avoiding health risks and help with IGA. Economic assets gave negotiation power and economic grounding to build on. Assets interacted and reinforced one another, potentially strengthening effects when girls possess all three.  Importance of sequencing: age-appropriate economic strengthening. Soft skills (self-esteem, communications) and basic financial education (money management & savings) need to be introduced early/mid adolescence for girls to deal with financial responsibilities that increase around 15 years (personal needs & contribution to household), becoming even more prevalent from 18 years. Then, focus should be on economic empowerment to bolster human assets (vocational/employability training) and economic assets (work-integrated learning, mentors and business capital). Also important is to help build girls’ social assets. |

1. **ELA-Uganda**

| **Programme/Project Name** | Empowerment and Livelihood for Adolescents (**ELA**) (2-year programme) -Uganda |
| --- | --- |
| **Paper(s)** | **Bandiera 2015 (2-year project)**  Bandiera O, Buehren N, Burgess R, Goldstein M, Gulesci S, Rasul I, et al. Women's economic empowerment in action: Evidence from a randomized control trial in Africa. Geneva: ILO, Department EP; 2015. Contract No.: 187.  **Bandiera 2018 (4-year follow-up)**  Bandiera O, Buehren N, Burgess R, Goldstein M, Gulesci S, Rasul I, et al. Women’s empowerment in action: evidence from a randomized control trial in Africa. eLibrary: World Bank; 2018. |
| **Implementing Partners** | Bangladesh Rural Advancement Committee (BRAC) -microfinance  International Labour Organization (ILO) |
| **WHAT?**  **Stated aim**  **Type of HIV-sensitive social protection** | ELA is a two-pronged intervention offering vocational and life skills training to economically and socially empower girls and enhance control over their bodies.  **2015**: To evaluate impacts of ELA programme (IGA and life skills training) on economic, social and sexual health lives of 14-20-year-old adolescent girls at 2 years follow-up.  **2018**: same at 4 years follow-up to assess sustained impact  Livelihood or employability aspect: IGA training, life skills (SRH/marriage/gender/legal rights/management skills).  HIV-link: teenage pregnancy, early marriage/cohabitation; expected marriage/fertility, GBV, sexual behaviour change (condoms). |
| **Design** | CRCT |
| **WHEN?** | 2-year intervention (2008-2010) + additional 2 years without training but with continued safe space (2010-2012)  Baseline March-June 2008; set up clubs June-Sept 2008; follow-up survey March-June 2010 (considered as midline in Bandiera 2018); follow-up survey March-June 2010 (endline) |
| **WHO?**  **Sample description Inclusion criteria** | 14-20-year-old female AG (baseline: mean age 16 years)  Inclusion: only gender & age to also get out-of-school girls  Baseline: n=5,966 (3963 intervention; 2002 control); 2015 follow-up n=4,888 (82% tracking rate); 2018 follow-up n=2002 tracking rate 65%  100 communities (50:50) with around 40 girls/community |
| **WHERE?**  **Country & Context** | **Uganda** – 5 BRAC branches in (peri) urban regions of Kampala & Mukono; 5 branches in rural regions around Iganga & Jinja. 15 communities per branch randomly assigned to intervention (n=10) and control (n=5). Total intervention communities (n= 100) and total control (n=50). |
| **HOW?**  **Description HIV-sensitive social protection intervention** | Two-pronged intervention with vocational training and life skills for SRH/HIV knowledge. Intervention delivered in development clubs rather than in schools to target out-of-school girls.  BRAC has 1200 clubs in Uganda reaching 50,000 girls. Club participation is voluntary and unrelated to other BRAC activities. Clubs have a fixed place and are open 5 afternoons/week. Activities (reading, staging plays, singing, dancing, playing games) are led by female mentors (from community, slightly older).  Intense and extended training period (2 years) covered general business skills + technical knowledge + sector-specific content & was combined with life skills and offered in a safe space for girls.  Local entrepreneurs, hired professionals, BRAC staff offered vocational training & financial literacy (budgeting, financial services, accounting). ELA takes local business environment and girls’ educational levels into account to create a demand-driven IGA with focus on self-employment like hairdressing, tailoring, agriculture, poultry-rearing, small trades.  Life skills: SRH topics (menstruation, pregnancy, STI, HIV, family planning, rape); management skills -or higher-order life skills- (negotiation, conflict resolution, leadership); gender issues (bride price, child marriage, GBV).  After 2 years, girls can continue attending clubs as safe space but do not receive further training.  Buehren 2017 reports that livelihood training package in Uganda also included productive asset transfers worth USD30 (seeds, tools, chicks). This was not reported in Bandiera 2015/18. |
| **Outcomes of interest** | 1. Economic empowerment (engaged in IGA, (earnings from) self/wage employment, entrepreneurial skills/) 2. Control over body (SRH/HIV knowledge, condom use, unwilling sex) 3. Aspirations (fertility, ideal age for marriage/childbearing (self and children)) |
| **Outcomes** | **Baseline:** 6% girls are self-employed; 30% out-of-school; 60% worry about not finding a job when an adult; 17% report having had unwilling sex in past year. They have limited SRH/HIV knowledge and 52% report always using a condom. Girls hold traditional views on gender roles; they feel girls should marry at age 24, 10% already have a child and 10% are already married or cohabiting.  **Bandiera 2015:** - reports ITT and TOT. Significant results for intervention girls:  *ECONOMIC EMPOWERMENT*  - Gender empowerment index higher: ITT: 9% (2.86 percentage points); TOT: 58% (18.3 percentage points)  - Self-reported entrepreneurial skills: ITT 8% (5.63 percentage points); TOT 50% (35.6 percentage points)  - Engagement in IGA: ITT: 72% (= 6.8 percentage points); TOT 43.4 percentage points, mostly due to self-employment; at endline intervention girls were twice as likely to be self-employed compared to control girls (ITT) and 6x for TOT (wage employment not significant)  - Earnings: increase of extensive (62% --the number of girls) and intensive margins (3x baseline –how much); impact earnings larger than hours of work = marginal product of labour  - Vocational training did not have adverse effect on school attendance; even a higher proportion of dropouts considered returning to school.  *WELFARE*  - Not worrying about getting a job when adult: ITT 18% (7.2 percentage points); TOT 45.3 percentage points.  - Increased monthly consumption expenditures (ITT 41%; TOT: > double baseline)  *CONTROL OVER BODY*  - Teen pregnancy falls by 26% (ITT, 2.7 percentage points = eliminates natural increase over 2 years)  - Delayed marriage/cohabitation 58% (ITT 6.9 percentage points = nearly eliminates natural increase over 2 years)  - Girls reporting sex against their will drops with 44% (6 percentage points); TOT: 24.6 percentage points, i.e., near elimination.  - Preferred ages of marriage and childbearing both move significantly forward (ATE nearly 5 & 4 years respectively. Aspirations for their kids are similarly reduced.  - Desire to have less kids =43% (1.77 percentage points): would close fertility gap with developed countries  Compared with baseline:  Increased pregnancy/HIV knowledge & 28% increase self-reported condom use.  **Bandiera 2018:** 4-year follow up (2-year results are midline results) found significant results:  *ECONOMIC EMPOWERMENT*  - Self-reported entrepreneurial skills sustained: ITT 3% increase  - Engagement in IGA: ITT: 48% (= 4.9 percentage points); self-employment rates remain 50% (vs twice at midline) higher than in control (9.5 percentage points): death rate IGA from first year to endline = 15%.  - Expenditure (proxy for income) high at midline (39%) not sustained at endline.  - Economic empowerment index significant and robust at 5% (vs. 1% level midline)  - ELA does not negatively impact formal education at endline  *CONTROL OVER BODY*  - Fertility (having a child) 34% lower (3.8 percentage points) vs 24% drop midline  - Delayed marriage/cohabitation 62% (ITT percentage points) vs. 53% midline  - 5.3 percentage points (vs. 6.1 percentage points at midline) lower unwilling sex = nearly 30% reduction incidence  - Increased condom use by midline (13 percentage points) dies out by endline  - Control over body single index increased and are larger than econ empowerment index.  ASPIRATIONS  - Gender empowerment index higher at midline; no statistical difference by endline  - Fertility expectations & aspirations for their kids died out but shifts in ideal age for marriage and childbearing endured: marriage: 25% (0.77) at midline & 8% (0.23) by endline; ideal age for their own wedding almost a year later (compared to control); ITT childbearing: 20% midline and 9% endline.  SPILLOVER EFFECTS  Despite participation rates of only 20%, all 3 dimensions of female empowerment (ITT) are economically and statistically significant (stronger for control over body and weaker for economic empowerment).  No girl loses from ELA but gains from participation vary considerably (estimated with marginal treatment effects (MTE)).  ATT: larger than ITT impacts: average gains participants are more than double than for non-participants.  Spillover effects re index econ empowerment: 8%; 27% control over body index & 43% for aspirations index.  *MEDIATORS*  Life skills important mediator for all dimensions of empowerment at midline; only for economic empowerment by endline. Large fraction baseline ITT remains unexplained by mediators, likely explained by safe space for girls and role models mentors.  **Process data**: Club participation rate is 21% among intervention girls vs 4.7% among controls but latter become negligible at 18 months. No significant differences between (non)participants. Those who do participate did so intensely (once or twice/week for 2-year duration). At 2-yrs, 84.7% took part in life skills training; 52.7% in vocational skills training; 50.9% took both, so only 1% did only vocational part. Authors deduce that majority participants saw 2 parts as complementary. Afterwards no drop off to end line (girls must have appreciated safe space).  After 2 years, half of 100 intervention communities were supposed to offer microfinance to older girls but very few offers were made; terms of microfinance were not so different from what was already available resulting in near zero uptake. |
| **Comments** | Results are robust across rural/urban; rich/poor; young and old girls & not driven by anticipation MFI (offered year after 2-y evaluation).  **Bandiera 2015:** authors claim that it is the confluence of offering two complementary programmes during adolescence (when employment choices are still fluid) seem critical to success. They refer to literature to compare their results with stand-alone programmes that showed limited impacts.  Near elimination of unwilling sex likely results from (1) life skills sessions (negotiation, rape and legal rights) that sensitized girls + allowed them to take preventive measures; (2) clubs offering safe place, esp. after school when parents may not be back from work; (3) economic empowerment likely reinforces girls’ control over their bodies.  On top of large economic, social and body control impacts, the intervention is about changing norms (aspirational outcomes for marriage and childbearing ages) for the next generation.  **Bandiera 2018:** interventions for adolescent girls may have higher returns than later-timed interventions. Multi-faceted interventions recognize multidimensional nature of women’s empowerment. Authors frame vocational component as ‘hard’ skill, recognizing external constraints; life skills to make informed choices about sex, reproduction and marriage as ‘soft’ skills to address internal constraints (knowledge, self-confidence, aspirations); and clubs as safe spaces for socialization free from male pressures.  Future work: need to further unpack black box (relative importance of each component, effect older mentor; complementarities; interaction with men (impact earned income, bargaining power, autonomy, changed attitudes men; matching with better quality men –virtuous circle that widens women’s economic opportunities and supports economic development). |
| **Cost** | 2015: cost ELA Y1: US$365,690; Y2: $232,240  This intervention was cost effective: Y2 or Y4 cost/girl $17.9 (ITT) vs. higher ITT labour market impact (girls made $32.8 (2015); 2018: $50)  $17.9 is <1% of annual income/HH.  Considering 21% take up rate, TOT cost is $85/girl. This compares favourably with other programmes (Blattman 2011: $374 & return on capital 35%; Latin America *Jovenes* $600-2000/participant)  Gains made in delayed marriage/fertility, improved HIV & pregnancy knowledge, self-reported condom use, and dramatic reduction unwilling sex are difficult to cost, and gains will be over life cycle, but early marriage, children & GBV interrupt human capital accumulation and permanently affect lifetime earnings adversely. |

1. **ELA-Tanzania**

| **Programme/Project Name** | Empowerment and Livelihood for Adolescents (**ELA**) (2-year programme) -Tanzania |
| --- | --- |
| **Paper(s)** | Buehren N, Goldstein M, Gulesci S, Sulaiman M, Yam V. Evaluation of an adolescent development programme for girls in Tanzania. e-Library: The World Bank Group; 2017. Report No.: 7961. |
| **Implementing Partners** | BRAC (microfinance –(MFI)) |
| **WHAT?**  **Stated aim**  **Type of HIV-sensitive social protection** | To evaluate replication of ELA intervention in Tanzania.  ELA offers two-pronged intervention offering vocational & life skills training to economically and socially empower girls & enhance control over their bodies.  Livelihood or employability aspect: vocational training, life skills (to make informed decisions about SRH/marriage), microcredit + business and financial literacy training.  HIV-behaviours: (empowerment), teenage pregnancy, early marriage/cohabitation; expected marriage/fertility, GBV, sexual behaviour change (condoms). |
| **Design** | CRCT |
| **WHEN?** | 2-year intervention (2009-2011)  Baseline Jan-July 2009; set up clubs July-Dec 2009; microfinance rollout early 2010; follow-up survey June-Nov 2011 |
| **WHO?**  **Sample description Inclusion criteria** | 13-19-year-old female AGYW (baseline: mean age not reported; participation not restricted to this age bracket)  Baseline: n=5,454 (3963 Rx; 2002 control); follow-up: 3,179 (42% attrition rate –baseline schoolgirls and mothers less likely to attrite; nonetheless no selection bias)  100 intervention communities A: Club only; B: Club + MFI); 50 control; 30-40 girls/community |
| **WHERE?**  **Country & Context** | **Tanzania** – 10 BRAC branches in mix rural/urban regions of Iringa & Dodoma. 15 communities/branch = 150 communities; randomly assigned to 100 intervention and 50 control communities. |
| **HOW?**  **Description HIV-sensitive social protection intervention** | Multi-pronged intervention with livelihood component: (1) vocational training; (2) life skills for SRH/HIV knowledge; (3) safe space (development clubs to target out-of-school girls); (4) community participation. & (5) microcredit -including business and financial literacy training)  (1) Local entrepreneurs, hired professionals, BRAC staff offer livelihood (vocational) training: IGA training suitable for local context: small-scale agriculture, poultry-/livestock-rearing, hairdressing, tailoring, small trades. Sometimes financial education.  (2) Female mentors (from community, few years older) facilitate general club activities and conduct life skills training: SRH topics (menstruation, pregnancy, STI, HIV, family planning).  (3) Safe space: adolescent development centres (clubs) with books and games.  Easy to reach, fixed place; open 5 afternoons/week. Activities (reading, games, staging plays, singing, dancing, socializing); voluntary participation leveraged to conduct skill training.  (4) Community participation to create an enabling environment and community ownership and support: periodic meetings with parents and village elders to sensitize them about ado girl issues.  (5) Microfinance: financial means provided to older adolescent to engage in self-employment activities. Financial literacy + individual business support (planning/management) part of loan process. Loans are smaller than regular microcredit, but process is similar (10% deposit, 25% interest rate, repayment frequency -40 equal instalments) |
| **Outcomes of interest** | 1. Uptake intervention 2. Economic empowerment ((planning to become) engaged in IGA, daily/labour income, financial skills/participation (loans, savings)) 3. Social outcomes (education, HIV knowledge, ever had sex or a sexually transmitted disease (STD), condom use, unwilling sex, fertility, desired age marriage/childbearing for self and children, perceived control, talk with friends about business/social issues) |
| **Outcomes** | **(1) Uptake** ELA: >50% girls in each group knew about ELA; Club+MFI more likely to know (64%) but not statistically different. Take up rates: 19% Club+MFI; 13% Club only; 7% control (vs. Uganda 21% without MFI). MFI increased take up with 6 %p but only 4% in Club+MFI ever participated in MFI component. MFI increased overall interest in programme, especially among girls who were not interested at baseline. Among those interested at baseline, interest was very high (9/10).  **(2) Economic empowerment**: none of components showed significant impact (ITT, ATE) except for financial market participation: Club+MFI increased savings, especially informal savings (*Upatu*, or rotating savings/credit schemes) by 2.8%p (from 2% baseline). No impacts on borrowing despite main product MFI being credit. Savings were rather a spillover effect of participants’ social network.  **(3) Social outcomes**: None significant except for gender roles in Club + MFI (ITT) and at follow-up, talk about business with friends (correlations social network & informal savings).  **Qualitative process evaluation** (Buehren 2017 summarizes Yam 2013): resource constraints underlie replication problems: lack of funding led to asking communities for donation of space and not replenishing club materials (instruments, books, games etc.). Donated spaces restricted club activities and were often insecure.  Inadequate training of new mentors, long waiting times for replacement, less frequent monitoring and supervision visits: area manager in Tanzania supported 10 branches vs 5 in Uganda. Uganda had MasterCard Foundation funding, allowing for quick scale up; economies of scale; more activities like inter-club competitions.  Different priorities among target population: Ugandan girls were more interested in the livelihood component whereas Tanzanian girls would have preferred supplementary tutoring to support education. Tanzania’s higher school enrolment rate 80% (vs 70% in Uganda) might explain this. |
| **Comments** | Lack of implementation fidelity is key driver of contrasting results. Layering financial services on non-financial training programmes influenced greater participation, higher savings and is plausible avenue for youth-inclusive financial services.  General programme design was not implemented in Tanzania. They had problems selecting implementation sites. They started with 10 urban BRAC branches and listed 200 communities, but lack of interest & limited cooperation communities forced redeployment of teams to 10 less urban branches (Dodoma & Iringa). Subsequently, there were financial, human resources and time constraints. They had to restart surveys.  Livelihood support: in Uganda girls received $30 worth of in-kind support (seeds, tools, chicks) but in Tanzania this was not offered. Continuation ELA Tanzania focuses on education.  Unlike ELA Uganda, where business training and financial literacy were offered as life skills training (87% uptake), ELA Uganda seems to have made this part of the microcredit component. Microcredit suffered low uptake of 4%, so does this mean that business and financial literacy training also suffered same low uptake? |

1. **IMAGE**

| **Programme/Project Name** | Intervention with Microfinance for AIDS and Gender Equity (**IMAGE**) |
| --- | --- |
| **Paper(s)** | Pronyk PM, Kim JC, Abramsky T, Phetla G, Hargreaves JR, Morison LA, et al. A combined microfinance and training intervention can reduce HIV risk behaviour in young female participants. Aids. 2008;22(13):1659-65. |
| **Implementing Partners** | Small Enterprise Foundation (microcredit)  South African domestic violence NGO (sisters for life) |
| **WHAT?**  **Stated aim**  **Type of HIV-sensitive social protection** | To assess whether IMAGE influenced HIV risk among young women who were direct participants.  IMAGE aims to create an enabling environment for behaviour change by addressing poverty and gender inequality as key structural factors underlying HIV risk.  Livelihood or employability aspect: microcredit, business training, mentoring (for which outcomes are not measured in this study); Livelihood or employability component measured: communications skills  HIV-link: HIV knowledge, communications about sex, safe sex, HIV infection (14-35 years) |
| **Design** | Mixed Methods: CRCT - This is a sub-study of larger IMAGE trial with young women: 2 surveys (baseline + 2-year follow-up) + qualitative interviews |
| **WHEN?** | 3-year intervention: full implementation from 2001-2004.  Pilot 1998-2000 (not part of this study –described in Pronyk 2005)  Baseline early 2002; endline from 2003 through to April 2005 |
| **WHO?**  **Sample description Inclusion criteria** | 14-35 years old (mean age 29 years)   1. Poorest households   Matched pairs: 262 at baseline; 220 at endline |
| **WHERE?**  **Country & Context** | **South Africa** - Limpopo Province  Rural area with villages between 2-20 km from main trading centre. Major sources of income: government grants, local public sector employment, migrant remittances. Subsistence agriculture is not viable for most people. |
| **HOW?**  **Description HIV-sensitive social protection intervention** | Group-based micro-credit model (Grameen-model):  **Process**: Participatory wealth ranking identified women age 18 and older from the poorest families in a village. Five women form a group and serve as each other’s guarantors. New credit can be obtained once all have repaid their respective loans. Loan centres (n=40; 8x5) organize biweekly meetings to repay loans, apply for new loans, and discuss business plans.  Participatory curriculum (Sisters-for-Life) is integrated in loan centre meetings for group-based learning, to foster solidarity and collective action.  Phase 1: ten 1-hour training sessions (gender roles, cultural beliefs, relationships, communication, domestic violence, HIV infection) to strengthen critical thinking, communication skills, leadership; foster solidarity and action for HIV prevention.  Phase 2: Peer-selected natural leaders received 1 additional week training to identify priority issues (HIV and IPV) and instigate wider community mobilization by engaging youths and men in intervention communities. |
| **Outcomes of interest** | (1) HIV knowledge and communications in home (12 m) and feeling comfortable discussing sex at home;  (2) Access to voluntary counselling and testing (VCT); having gone for testing  (3) sexual risk behaviour (last 12 months): multiple partners; unprotected sex with last non-spousal partner |
| **Outcomes** | **MFI**: no results reported in this paper  **HIV**: Only significant for:  - Increased communications about sex 12 months aRR 1.46 (1.01–2.12);  - Having gone for testing increased: aRR 1.64 (1.06–2.56)  - Unprotected sex with last non-spousal partner reduced: aRR 0.76 (0.60–0.96);  HIV incidence was too low (n=8) to examine impact of the intervention.  **QL** At first, discussing HIV (testing) and sex was taboo. As women internalized risk they overcame this fear and became more confident negotiating safe sex. It was easier to discuss this with children in the household than with partners. They felt fear re VCT (social stigma & emotional distress with positive result). |
| **Comments** | Authors conclude that the intervention could have influenced young women’s sexual behaviour, the results indicating a potential for synergy as the literature indicates that increased testing has protective effects on sexual risk behaviour & increased communications can facilitate behaviour change.  Unlike Pronyk 2006 -the Lancet paper that reports on IPV and HIV incidence, this 2008 paper looks at the outcome indicators used for young women (14-35 years) who participated in the full trial. The young women (14-35 years) in the 2006 paper were household members of women who participated in MFI: HIV knowledge, sex communications, VCT & sexual behaviour change. |

1. **SCIP**

| **Programme/Project Name** | Women First (part of Strengthening Communities through Integrated Programming (**SCIP**)) 18-months study |
| --- | --- |
| **Paper(s)** | **Burke 2019** (quantitative (QN) study)  Burke HM, Field S, González-Calvo L, Eichleay MA, Moon TD. Quasi-experimental evaluation using confirmatory procedures: A case study of an economic and social empowerment intervention to reduce girls’ vulnerability to HIV in rural Mozambique. Evaluation and programme planning. 2019;77:101721.  **Burke 2019** (qualitative (QL) study)  Burke HM, Packer C, González-Calvo L, Ridgeway K, Lenzi R, Green AF, et al. A longitudinal qualitative evaluation of an economic and social empowerment intervention to reduce girls’ vulnerability to HIV in rural Mozambique. Evaluation and programme planning. 2019;77:101682. |
| **Implementing Partners** | World Vision - USAID |
| **WHAT?**  **Stated aim**  **Type of HIV-sensitive social protection** | To assess impact of a combined economic and social empowerment intervention on adolescent girls’ HIV vulnerability (girls’ sexual behaviour, school attendance, and community norms) and explore perceived pathways/processes (implementation and sustainability).  Livelihood or employability aspect: business training, kits for door-to-door selling, (savings, loans)  HIV-link: self-assessed HIV-risk (including HIV test), intimate partner violence (IPV) |
| **Design** | Mixed methods  **QN**: clustered non-equivalent two-stage cohort trial; multilevel exact matching & difference-in-difference (DID) estimation; confirmatory study.  **QL**: in-depth interviews (IDI) & focus group discussions (FGD) |
| **WHEN?** | Women First was implemented with varying starting dates from 2010- Sep 2015 (5 years)  Baseline Aug-Oct 2015; endline March-May 2017  **QN**: 2 rounds at 6-months intervals (R1 April-May 2015; R2 Oct-Dec 2015)  **QL**: 2 rounds: 1: Aug-Oct 2015; 2: Aug-Oct 2016 (mostly with same participants from round 1) |
| **WHO?**  **Sample description Inclusion criteria** | 13-19-year-old vulnerable girls (13-17 at intervention start; QN: mean age 15.5; QL: mean age 16 years): having lost at least one parent, living in child-headed household, engaged in transactional sex or other HIV risk behaviours. Lack of knowledge regarding HIV transmission, GBV and unwilling to report GBV/lack of access to HIV prevention services.  **QN**: Round 1: n-=885: 266 Rx girls – 619 comparison (10 girls randomly sampled from 65 comparison communities); Round 2: n= 774 (Rx 232; comparison 542; retention 87%); during analysis 59/233 and 266/589 girls were dropped, lacking exact match.  **QL**: in 12 intervention districts & 2 communities per district:  IDIs with 49 girls, 36 influential males, 24 heads of households; 12 FGD with 6-11 community members: government, local leaders, Women First staff. |
| **WHERE?**  **Country & Context** | **Mozambique** – 22 communities in 6 districts of Zambézia Province: Gurué, Alto Molocuè, Ilê, Mopeia, Mocuba, Morrumbala. |
| **HOW?**  **Description HIV-sensitive social protection intervention** | 2-pronged empowerment intervention: economic + social  **Economic intervention**: business education and increasingly valuable business kits. Girls had to sell kits and repay them. After third kit was sold, girls graduated and were eligible to receive a bicycle. Kits contained soap, baking ingredients (flour, sugar, oil); sometimes also batteries, rice biscuits, spaghetti.  Some communities provided group saving options and linked business capital (loans) through accumulated savings and credit associations, introduced to women by SCIP in Women First communities and later made available to girls.  **Social intervention**: locally tailored GBV curriculum to reduce girls’ vulnerability to HIV and to encourage them to stay in/return to school. Facilitator–led education sessions: gender norms, how to communicate with adults/partners, pregnancy, HIV prevention, preventing unwanted sexual advances, planning goals, assessing values, money, gifts.  Community leaders, together with adult Women First participants & SCIP staff invited girls. Hardly any girl refused to participate; there was more interest than available places. |
| **Outcomes of interest** | **QN**   1. Self-reported 6-month incidence of intergenerational & transactional sex 2. Knowledge HIV/GBV 3. Willingness to report/seek medical help for GBV 4. School attendance   **QL**  Perception of intervention; experience earning money; spending; how it affected relationships with others; community involvement; ideas about dating, premarital sex, marriage, ideal partner age, condom use, future goals.  Round 2 asked explicitly about pathways/processes of the intervention and HIV risk among girls; community norms, implementation/sustainability. |
| **Outcomes** | **Quantitative results:**  No evidence of impact intervention on girls’ GBV knowledge, school attendance. For five other outcomes no accurate measures could be obtained, so impact was not assessed. Incidence for transactional sex (7%) and intergenerational sex (1%) was lower than expected. It was impossible to distinguish actual and reported sexual behaviour as there was no valid measure. Age could not reliably be established. Effective sample size after stratification/matching was too small and time period might have been too short to detect changes.  **Qualitative findings**:  Only one influential male was a boyfriend; most influential males did not live with girls. Most head of households were female. In round 2, several girls had married and had kids.  **Business**: Round 1: ¾ girls earned money and were satisfied with the amount. They spent it on clothes, business products, school items, savings, and household items. Round 2: more than half reported earning money past year; ¼ still at time interview (selling products or working on farm/sell produce). FGD: very few girls continued doing business because they were unable to sell products due to the lack of diversity of products, profits being too low, repayments too high, owing money to the intervention, high travel costs, high costs of unsubsidized products, business component was too short. Additional barriers were drought and hunger.  **Sexual behaviour**: attitudes towards girls’ (premarital) sexual activity were negative and stigmatizing; few mentioned learning about negative health impacts (STI, HIV, unintended pregnancy). Round 1: around 1/3 and 11/12 respondents mentioned that the intervention contributed to reduction of transactional sex but in Round 2 many re-engaged out of financial need or got married due to financial pressure and despite ideal marrying age being 18.  A few respondents credited intervention with reducing early marriage and pregnancy.  **GBV & intergenerational sex**: most girls seemed to have superficial knowledge about GBV at both rounds; ¼ (Round 1) and ½ (Round 2) learned about reporting/seeking health care. GBV/HIV awareness among community members was high, but there was limited evidence that it translated into practices. There was no evidence of spill over effects, except for reports of adult men fearing punishment for sex with minors (crime). Both girls and other respondents say that GBV reduced in community.  **School attendance**: half of the girls mentioned being in school at Round 1; 1/3 in Round 2; 1/3 report having dropped out since the end of the intervention. Girls’ educational aspirations had increased. With earnings they were able to buy fees/uniforms/ school items, also for siblings. Some parents also reported improved attitudes towards school.  **Sustainability**: school was sustained for some; girls’ respectful behaviour and reduced promiscuity seemed to be sustained.  **Pathways**: financial resources empowered girls to refrain from high-risk behaviours; intervention changed household and community social norms re how they perceived and treated girls.  Decreases in stigmatized behaviours were highly valued by social environment but once the economic component was not sustained, girls had to resort back to high-risk behaviours.  **Recommendations**: diversify products, strengthen links suppliers-girls for their business network after end intervention, implement for longer duration, strengthen savings component; add negotiation & resilience skills. GBV component was too superficial: need for critical reflection and include men. |
| **Comments** | QN study had several programmatic/logistical challenges: authors were asked to conduct a prospective study after intervention had already started; no random sampling Rx communities; small sample size; lack fidelity programme implementation (varying start dates, implementation programme components). Selection criteria had not been formalized so comparison girls were non-equivalent.  From description intervention it is not clear what the length of GBV/business training was; description business component is not reported. |

1. **SHAZ!**

| **Programme/Project Name** | Shaping the Health of Adolescents in Zimbabwe (**SHAZ!**) (2-year programme) |
| --- | --- |
| **Paper(s)** | **Dunbar 2010** (feasibility study –micro-credit)  Dunbar MS, Maternowska MC, Kang M-SJ, Laver SM, Mudekunye-Mahaka I, Padian NS. Findings from SHAZ!: a feasibility study of a microcredit and life-skills HIV prevention intervention to reduce risk among adolescent female orphans in Zimbabwe. Journal of prevention & intervention in the community. 2010;38(2):147-61.  **Dunbar 2014** (SHAZ! II pilot study –micro-grants + vocational training)  Dunbar MS, Dufour M-SK, Lambdin B, Mudekunye-Mahaka I, Nhamo D, Padian NS. The SHAZ! project: results from a pilot randomized trial of a structural intervention to prevent HIV among adolescent women in Zimbabwe. PloS one. 2014;9(11).  **Dunbar 2017** (case study SHAZ! II)  unbar MS, Mudekunye-Mahaka I. Empowering Adolescent Girls and Women for Improved Sexual Health in Zimbabwe. In: Kurebwa J, Dodo O, editors. Participation of Young People in Governance Processes in Africa: IGI Global Publisher Online Bookstore; 2017. |
| **Implementing Partners** | Red Cross (SRH trainings; home-based care)  Local NGOs  Zambuko Trust (MFI) |
| **WHAT?**  **Stated aim**  **Type of HIV-sensitive social protection** | SHAZ! addresses gendered poverty and agency; fosters women’s empowerment by which AGYW gain resources and capabilities to effect one’s own life, including making sexual health and wellbeing decisions (Kabeer 1999)  **Dunbar 2010**: To test the feasibility of a combined microcredit and life-skills HIV prevention intervention among 50 adolescent female orphans in (peri-) urban Zimbabwe: (1) identify methods for recruitment/retention; (2) examine appropriateness microcredit intervention; (3) refine intervention components and evaluation tools.  **Dunbar 2014**: To assess feasibility/efficacy of SHAZ! intervention: (1) feasibility recruitment/retention adherent female orphans; (2) changes in sexual behaviour and structural risk factors; (3) trends HIV/HSV2 incidence & unintended pregnancy.  **Dunbar 2017**: qual paper to complement 2014 paper.  Livelihood or employability aspect: life skills, vocational skills, MFI & mentorship.  HIV-link: (empowerment), unintended pregnancy, sexual behaviour change + HIV biomarkers (HIV & HSV-2 incidence) |
| **Design** | Dunbar 2010: mixed methods (6 months pre/post + interviews, FGD & process data) Dunbar 2014: RCT (pilot –Phase II RCT)  Dunbar 2017: case study |
| **WHEN?** | Formative work (2000): HIV risk & vulnerability adolescent girls, NB maternal orphans  Baseline early 2002; endline from 2003 through to April 2005  **Dunbar 2010**: 6-month pilot in 2004  **Dunbar 2014 & 2017**: 2-year intervention (2006-08); enrolment Feb-August 2006; last interview Dec 2008; data collection at baseline & every 6 months for 24 months |
| **WHO?**  **Sample description Inclusion criteria** | 16-19-year-old female orphans (2010: mean age 17.5 years; 2014: 18 y)  Uninfected, out of school, not pregnant  **Dunbar 2010**: QN survey n=50 (49 with complete data; descriptive stats) (Kang 2007); QL: 13 semi-structured interviews with loan recipients (factors business start-up/management & loan repayment); monitoring reports mentors; FGD post-intervention to improve life skills training.  **Dunbar 2014**  Convenience sample n=315 (158 Rx vs 157 control); 60 discontinued; 36 lost-to-FU |
| **WHERE?**  **Country & Context** | **Zimbabwe** – Chitungwiza & Epworth: (peri-)urban communities near Harare |
| **HOW?**  **Description HIV-sensitive social protection intervention** | Combined life skills and SRH/HIV services for all; livelihood package for intervention participants only.  **Dunbar 2010**: feasibility study offering life skills and microcredit to identify methods to recruit/retain participants; examine appropriateness microcredit for target population; refine intervention/evaluation.  Life skills curriculum based on *Talk Time* (basic HIV info) & *Stepping Stones* (HIV, gender, communications & relationship skills) to strengthen girls’ capacity to confront social/cultural forces that contribute to HIV risk.  Livelihood (IGA training): (1) 5-day business training (goal setting, business plans, cash flow management); (2) 4-day skill building workshops (candle/soap making & tie dye); (3) mentoring: matching of successful businesswomen with younger and less educated participants. Mentors were identified through community outreach.  Microcredit loans: group lending with weekly repayment meetings and new loans contingent upon full repayment. Grant size: US$51-87. Due to girls lacking bank accounts MFI also functioned as an informal bank. Interest rates were 30% (vs 50-60% commercial lending) to be repaid within 3-9 months.  **Dunbar 2014**: pilot study assessing feasibility to recruit/retain participants; examine changes in behavioural and structural risk factors; trends in HIV/HSV-2 incidence. Refocused on economic opportunities without repayment, training and support.  (1) Access to health services (SRH, HIV –screening and treatment at every study visit, free condoms, contraception pills or injection upon request, HIV referral, payment of CD4 tests)  (2) Life skills-based HIV education (see 2010: 14 modules over 4-6 weeks; 25/group) & home-based care (6 weeks, by the Red Cross)  (3) Economic livelihoods with cognitive, material and social support:  Livelihoods: basic financial literacy + support for nationally accredited vocational training (average 6-months with practical & theoretical components) of their choice. Once passed, they developed a business plan and received a micro grant (value US$100 in the form of supplies, capital equipment or further training)  Integrated social support: guidance counselling & self-selected adult mentors  Courses: hairdressing; tailoring; receptionist/secretarial professions, nurse-aide training |
| **Outcomes of interest** | (1) Economic factors: Income (MFI elements); food insecurity (2014 only)  (2) Social factors (relationship power, SGBV, social support received)  (3) Sexual risk behaviour & SRH/HIV Knowledge  (4) Biomarkers (HIV, HSV-2); pregnancy (unintended) |
| **Outcomes** | **Dunbar 2010:** significant results:  **Quantitative results:** Increases in income (44% vs 6% -could be caused by loan), HIV knowledge (38% vs 16%), relationship power in non-sexual romantic relationships. No significant changes in future plans/aspirations, condom use, sexual activity.  **Qualitative findings**: facilitators: previous business experience/capital, family support, alternative source of additional finance. Barriers: overall macroeconomic environment; girls’ vulnerability. 10/14 went into buying & selling: hazardous, transport costs, theft, confiscation goods, outstanding credit, threats personal safety (intimidation by police/men).  **Process data**: 98% overall satisfaction with SHAZ!-I; high and consistent participation in training: 80% attendance, 80% business plans. Perceived usefulness: life skills 98%, business training 97%, mentorship 60%. At 6 months, repayment was much lower than expected: 20% (only 6% had repaid in full). Lack of trust between mentors and participants: exploitation vs laziness; mentors lacking time; mentees lacking money for transport.  **Dunbar 2014:**  **Results:** significant results in reduced food insecurity, having own income, less transactional sex, condom use; marginally significant results for less SGBV, fewer unintended pregnancies (40%). No statistically significant changes for contraceptive use, HIV and HSV-2. Overall incidence was high for HIV (2.3/100), HSV-2 (4.7/100), and unintended pregnancy (10.8/100); HSV-2 incidence was higher among intervention girls.  Social support, relationship power & sexual activity was the same across study arms.  **Process data**: No statistical difference in retention at 24 months between intervention and control girls (84 vs 78%) or training completion (82% vs 73%), although there was a trend towards higher completion among intervention girls.  Among intervention girls: 124 (70%) started vocational training; 100 passed (63%); 92 received grants (NB Dunbar 2017 states 86 = 54%). Those not receiving grants had not completed training/business plan (24), returned to school (11), relocated (30). It was unclear what reasons were for non-completion of training and business plans but data from guidance counselling visits revealed language barriers and family demands.  **Dunbar 2017** reports findings from a new analysis of the 2014 paper with some differences: 2014 states 92 (60%) received grants vs Dunbar 2017: 86 = 54%). Intervention girls also improved ability to afford medications. Outcome for unintended pregnancy: magnitude & statistical significance of effect size increased.  Barriers: accredited vocational language courses taught in English instead of Shona; length of the course (6 months); family demands.  Those who had completed secondary education were thrice as likely to complete vocational training; those with an ill family member were 6% less likely to complete training. |
| **Comments** | **Dunbar 2010**: SHAZ!-I Offered adapted MFI with lump-sum loan instead of weekly instalments upon timely repayment; no prior savings or other collateral were required so it lacked the social pressure to repay. Without ensuring adequate social support, negative unintended consequences increased HIV risk (physical/sexual harm & coercion). Girls engaged in risky livelihood strategies like cross-border trading. Findings led to the adaptation of SHAZ!. They replaced loans with grants (without repayments) and added savings and conditional cash transfers to support formal education/vocational training and enhance family support.  **Dunbar 2014:** Due to the economic collapse in Zimbabwe, SHAZ!-II Was scaled down to a phase II RCT instead of the planned 1000 person trial to detect changes in biomarkers.  The lack of a true standard of care control arm + contamination (some control participants used compensation ($5/visit + $2 transport/day reimbursement) also for IGA (buying/selling), which may have diluted findings. There were few statistically significant outcomes across study arms despite statistically significant results when comparing pre-post indicators.  Evaluation period might have been too short (only 22% Rx girls had completed vocational training before 18-m visit, so economic benefits could have occurred later (likely after 24 months). Study was underpowered to detect biological differences, so the reduction of 40% unintended pregnancies is impressive.  Only 60% received micro-grants because of the overall contextual challenges, but also difficulties completing vocational training/developing business plans.  Lessons learnt: offer a wider-range of livelihoods options from basic to advanced; in the local language and at more flexible hours.  **Dunbar 2017:** Findings informed (unpublished) SHAZ-Plus (for AGYW living with HIV), which offered a wider range of livelihoods options from a 3-day course in the local language to medium length and nationally accredited courses. Life skills trainings were offered in weekends or evenings, over a shorter period of time. They undertook regular process evaluations to adapt the intervention. Currently enrolled: 715 participants with 80% retention over 18 months; 82% participants completed training + received micro-grants; working toward target of 85%. |

1. **SS&CF**

| **Programme/Project Name** | Stepping Stones & Creating Futures (2-year programme) |
| --- | --- |
| **Paper(s)** | **Jewkes 2014 (pilot)**  Jewkes R, Gibbs A, Jama-Shai N, Willan S, Misselhorn A, Mushinga M, et al. Stepping Stones and Creating Futures intervention: shortened interrupted time series evaluation of a behavioural and structural health promotion and violence prevention intervention for young people in informal settlements in Durban, South Africa. BMC public health. 2014;14(1):1325.  **Gibbs 2020 (CRCT)**  Gibbs A, Washington L, Abdelatif N, Chirwa E, Willan S, Shai N, et al. Stepping Stones and Creating Futures intervention to prevent intimate partner violence among young people: cluster randomized controlled trial. Journal of Adolescent Health. 2020;66(3):323-35. |
| **Implementing Partners** | Project Empower (NGO) |
| **WHAT?**  **Stated aim**  **Type of HIV-sensitive social protection** | To reduce GBV and HIV risk, interventions need to address poverty, women’s economic dependence on men, substance use and mental health by linking economic strengthening to gender transformative training.  Livelihood or employability aspect: critical thinking and livelihood training to leverage already existing resources  HIV-link: gender attitudes, IPV, rape, sexual behaviour change (last sex with main partner, condoms, transactional sex), HIV test |
| **Design** | **Pilot**: shortened interrupted time series–proof of concept study, not impact evaluation; mixed methods but this paper primarily reports cross sectional data.  **CRCT** (wait-list control) with 12- and 24-month follow-up |
| **WHEN?** | **Pilot**: 2012 – 2013 (1-year intervention - 58 weeks)  Surveys: 2x baseline (week 1, week 3), follow-up at 28 & 58 weeks; 3 FGD and 19 interviews with men (+ partners) before, at 6 months and at 12 months after intervention.  **CRCT**: 2016-2018 (2-year intervention – 12- and 24-months follow-up.  Baseline (recruitment) Sept 2015-Sept 2016; midline Feb-Nov 2017; endline March-Oct 2018 |
| **WHO?**  **Sample description Inclusion criteria** | 18-30-year-old unemployed and out-of-school youth  **Pilot**: 232 youth (2 women were 30+ years. At baseline/endline there were 122 and 111 women and 110 and 94 men)  **CRCT**: 34 clusters (20 men and 20 women per cluster – mean age was 23.8 years).  Baseline: n=1,351 (677 women; 674 men); midline (12-months: Feb-Nov 2017);  endline 24-months: n=1050 retained (545 women, 505 men) |
| **WHERE?**  **Country & Context** | **South Africa** –eThekwini district, KwaZulu Natal.  Informal urban settlements: overcrowded, lack of decent housing, utilities, sanitation, poor or no health services & roads.  Pilot: Little Japan & Mbazwana in eThekwini. Little Japan is located next to the highway near a large township,15-25 min to Durban by dirt road. Mbawanza takes 45 min from Durban, is poorer and relatively new, located on a steep hillside. |
| **HOW?**  **Description HIV-sensitive social protection intervention** | 2-component intervention: gender transformative training followed by livelihood component; facilitated by slightly older trained peers (3 hours, twice a week, for 12 weeks) to single sex groups of 20 participants.  **(1) Stepping Stones** (10 sessions): HIV and violence prevention programme aimed at more gender-equitable relationships and better communications. It draws on everyday reality through participatory learning, critical reflection, role play, drama.  Content: gender, peer influence, sex & love, conception/contraception, STI/HIV, safer sex/condoms, GBV, behaviour motivations (alcohol/poverty), communications skills  **(2) Creating Futures** (11 sessions): Participatory learning activities to critically reflect on livelihood/skill development using existing resources in their environment; goal setting; need for assets; coping with crises; social resources (trust and community participation); education & learning; building on past experiences; getting/keeping jobs; marketing skills; IGA; basic business training; strategies for saving/overcoming debt; coping with shocks. |
| **Outcomes of interest** | **Jewkes 2014**   1. Socioeconomic (earnings, hardship/crime, work stress, grants, supporting children, hunger, emergency funds, social capital) 2. Gender (attitudes, perpetration/experience of GBV & relationship control) 3. Health (mental health, sexual behaviour, HIV test, alcohol/drugs)   **Gibbs 2020**   1. IPV (physical, sexual, severe, emotional, economic), GBV 2. Socioeconomic outcomes (earnings past month, work shame/stress, stealing (hunger), emergency cash, savings) 3. Mental health (life satisfaction, depression, suicidal ideation) 4. Substance use (alcohol, problem alcohol, quarrel about alcohol) 5. Sexual behaviour (last sexual partner, transactional sex) |
| **Outcomes** | **Jewkes 2014** (below only reported for women)  **Baseline**: 80% <25year, 24% high school, 18.7% no current partner, 60% 1 child, 27% 2 children, 36% worked in past 12 m, baseline mean earnings R174 ($17), 54% financially supported their kids, 48.8% received child grant, 47% stole for food  **(1) Socioeconomic outcomes (Round 4)**: significant results: last month earnings R484 (=US$48 = 278% increase), feelings about work situation improved; 61% financially supported children, 56.9% received grant for child; 35% stole for food, more able to mobilize emergency money. No significant improvements in education, hunger, proportion borrowing money for food, crime, club/church membership, social cohesion  **(2) Gender outcomes (Round 4)**: significant results: gender attitudes, past 3 months sexual IPV; and combined physical/sexual IPV. None for physical IPV and relationship control.  **(3) Health outcomes** **(Round 4)**: significant results: improved life satisfaction, problem alcohol drinking increased from 26.6 to 35.5! but quarrelling about alcohol drinking reduced by half.  **Process**: challenges around attendance (60%)  **Conclusion**: this 2-pronged intervention has the potential to strengthen livelihoods, improve gender relations, & reduce gender violence.  **Gibbs 2020** (below only reported for women)  **Baseline**: all past year violence categories were very high: non-partner sexual violence (33%); IPV: physical (60%), sexual (30%), severe (65%), emotional (78%), economic (50%). 46% depressed; 20% post-traumatic stress syndrome (PTSD); 30% high food insecurity; 45% medium; 25% had stolen because of food insufficiency. Past month earnings were low (R169 =$11), ranging from $0 to $267.  **Outcomes**: significant (IT) at endline: 47% more past month earnings; 25% increase past 4-wk savings (b: .61, 95% CI: .18-1.03); more gender equitable attitudes at 12 months (not sustained at 24 months).  Per-protocol analysis showed that also depression and shame about lack of work were significantly lower. No difference in any of the IPV outcomes, but men’s self-reported violence perpetration did significantly reduce (severe, physical and economic IPV and positive trend sexual IPV & non-partner sexual violence).  **Process**: attendance of 3 or more sessions: 57% men & 71% women  **Conclusion**: Intervention should be scaled up but the lack of impact on IPV for women requires more research. |
| **Comments** | **Jewkes 2014**  Creating Futures does not require large financial investment like cash transfers or MFI so has the potential for scalability and transferability.  Unlike stand-alone Stepping Stones study, this pilot showed impact on experience of IPV among women. This supports a growing body of evidence that women need positive change in their material circumstances to leverage knowledge gained from gender-transformative programmes to reduce GBV. Nonetheless, men did not report significant reductions in violence perpetration (The Stepping Stones trial showed the opposite). Perhaps because the follow-up was too short (1 year instead of 24 months) or because the context of informal settlements was too harsh - violence is a ready resort and difficult to change. Positive results for men: increased mental health, HIV tests, and faithfulness. Women drinking more may result from more income; less quarrelling over women’s drinking may suggest that they had more conflict resolution skills.  **Qualitative** paper (Gibbs 2014): intervention contributed to a subtle shift from toxic masculinity to a more traditional form of masculinity whereby their improved livelihood allowed them to economic provision support a more stable and less conflictual household.  **Gibbs 2020**  During trial period there had been a significant investment in HIV/IPV prevention activities by other organizations in both arms (DREAMS with a shortened Stepping Stones version) so at mid/endline they added “attending workshops by others”. Although attendance to other workshops (35-45%) was high, there were no significant differences between arms, and they adjusted for it in analyses.  The intervention reduced men’s self-reported perpetration of IPV whilst women did not report experiencing reduction of IPV. This is similar to findings original Stepping Stones trial but contrary to pilot. Future studies should try recruiting couples to validate men’s self-report. For men, alcohol consumption also reduced, which may have contributed to reduced violence perpetration. Although women’s economic situation improved, their experience of IPV did not. Earnings/savings were still small and may have been insufficient to leave violent relationships. Moreover, economic theories of IPV assume stable monogamous relationships, whereas most young women did not live with their boyfriends and often had 2 or more ongoing relationships. |

1. **TRY**

| **Programme/Project Name** | Tap and Reposition Youth (**TRY**) |
| --- | --- |
| **Paper(s)** | **Erulkar and Chong 2005**  Erulkar AS. Evaluation of a Savings and Micro-Credit Program for Vulnerable Youth Women in Nairobi. New York: Population Council; 2005. 34 p.  **Erulkar 2006**  Erulkar A, Bruce J, Dondo A, Sebstad J, Matheka JK, Khan AB, et al. Tap and Reposition Youth (TRY): Providing social support, savings, and microcredit opportunities for young women in areas with high HIV prevalence. New York: Population Council; 2006. Contract No.: 23. |
| **Implementing Partners** | K-Rep Development Agency (KDA)  Population Council |
| **WHAT?**  **Stated aim**  **Type of HIV-sensitive social protection** | Overall aim of this multiphase initiative: to reduce adolescents’ vulnerabilities to adverse social and reproductive health outcomes in context of urban poverty and HIV infection, by improving their livelihood options (savings & credit) and social support.  Livelihood or employability aspect: microcredit, savings, business training, mentoring, safe space.  HIV-link: HIV knowledge & sexual negotiation of risky sexual behaviour |
| **Design** | Erulkar 2005 Longitudinal pre-post intervention with matched controls  Erulkar 2006 Case study |
| **WHEN?** | 4-year initiative  Data points over 36 months (baseline from June 2001; endline through to Dec 2004)  Pilot: 1998-2000 (Sebstad 2001) |
| **WHO?**  **Sample description**  **Inclusion criteria** | Out-of-school AGYW 16-22 years old (for pilot: 16-24 years) in urban slum areas in Nairobi  Pilot: 100 girls  Intervention: 326 baseline; 222 endline (matched pairs to control for socioeconomic status); more than two third of TRY girls were older than 20 years, often with older husbands/boyfriends (6.4 and 4.4 years older respectively) |
| **WHERE?**  **Country & Context** | **Kenya** –slum areas: Shauri Moyo, Mukuru, Majengo in Embakasi & Pumwani divisions, eastern Nairobi (600,000 people; lower middle class and urban poor; 20% lives in slums). Most participants came from slums or peripheries.  Urban slums in Kenya lack government infrastructure/services (water, electricity, health services, law enforcement), toilets, garbage collection; mostly 1-room houses made with semi-permanent material (mud, metal, wood); risk of flooding because of lack drainage system; high crime, job opportunities are mostly informal: petty trade & casual labour; attracts migrant from rural areas in search of education & employment. |
| **HOW?**  **Description HIV-sensitive social protection intervention** | Modified group-based micro-credit model in 4 phases to improve health & social outcomes:  Evaluations of phases 1-3 informed improvements in subsequent phases.  (1) pilot (1998-2000): minimalist model; locked up group collateral; social support  (2) 2001: loans require adult guarantors. In 2002: adult mentors & educational seminars were added (health & gender);  (3) 2004: assets replace adult guarantors. Savings-only clubs started: Young Savers Club with passbook; data collection endline  *((4) recommendation for next phase: vocational training, credit, on the job training for 'older & bolder')*  **Process**: Group (of 15-25 members) started registration with Ministry of Culture & Social Services, met weekly; K-rep procedures were explained; merry-go-rounds were established. Subgroups of five girls formed a *Watano* or saving group. Once registered, they opened a savings account and contributed US$0.65/wk. Each made a business plan. They selected the best two plans for which they submitted loan application. They received 6 days training. After 8 weeks of savings, group could receive first loan (=/>US$130/group) with an ROI of 15%. Group was collectively responsible for business/repayment & group savings served as collateral. If timely repayment for a month, the second 2 girls received their loans. If all 4 would repay timely for another month, the 5^th^ and final group member received a loan. Once first loan was repaid, a second loan could be requested and so on. Savings were locked up and inaccessible for the duration of participation. There was no grace period. The total repayment period was 3-12 (pilot) or 3-9 months (RCT).  Training (6 days) involved life skills, SRH info & discussion of social issues in addition to business and financial (planning) skills.  **Phase 2:** the added livelihood component aimed to improve capabilities and provide training/counselling by part-time adult mentors from different professional backgrounds (table 4). Mentors organized discussions, excursions, recreation activities, sports & seminars about gender awareness (gender-based violence, women’s rights, drug and alcohol abuse, male-female relationships); HIV and SRH (family planning) education.  **Phase 3**: 2004: stand-alone voluntary savings were added for those who wanted a safe place to save and enjoyed the social aspects of the club (meeting other girls, discussion, support, advice, mentoring)  **Businesses**: hairdressing; tailoring; battery charging, welding, operating telephone bureau & selling of vegetables, fruit juice, meat, firewood, and second-hand clothing. In rural areas: selling animal fodder, sawdust, firewood, telephone services, 2^nd^-hand clothes; animal husbandry, raising/selling poultry & running retail shops. |
| **Outcomes of interest** | (1) individual income, household assets, savings and keeping savings in a safe place; (2) liberal gender attitudes.  (3) HIV/SRH knowledge, sexual negotiation (condom use, risky sexual behaviour) |
| **Outcomes** | **Economic**: TRY girls were working more (from 44 to 57%); earned significantly more income (20%), savings (from 43 to 95%) although control had higher baseline savings (from 70 to 67%) & saved at safer places (bank; 42 vs 24%); Savings remained stable in 2002/03 but took off in 2004 with introduction of the voluntary saving scheme.  Older TRY participants (age 20 years and older) had significantly more assets, incomes, savings, safe savings.  **Social/health**: TRY girls had more liberal gender attitudes; significantly increased ability to refuse sex (OR 1.7) & insist on condom use (OR 2.86). However, the overall, the percentage of young women reporting the ability to refuse sex decreased. Also**,** at endline controls had more SRH/HIV Knowledge. |
| **Comments** | Small sample size and high attrition post-interview (32%) -included all dropouts.  66% dropout post intervention. Most were exposed to trainings, savings and mentoring but only 54% borrowed microloans. Girls were more interested in social aspects of groups and (informal) savings options.  Regarding borrowers: 93% felt it helped them; 56% found repayment (very) difficult.  Problems: inflexible group lending system, long waiting times to obtain credit (1-30 months with an average of 6 months) & inability to access savings locked up as collateral; divisive nature group collateral; lack of credit officers  49% respondents had moved houses in the last 3 years; only 17% of the original controls were located at endline: some controls were not interviewed at endline, others twice.  TRY expanded to rural areas in 2002-2003 (Kiambu -13.6 miles from Nairobi) where the intervention was more successful because of the small, close-knit community and reduced mobility: dropout rate was low there and repayment was high (85%).  Conclusion: mismatch project design with target group and intervention was late addressing the need for safe and accessible savings. Stand-alone savings and low-risk income generation activities were valued by (younger) girls. Recommendation: a comprehensive package, including social support, health education and financial services, implemented with a staged approach focusing on life skills, financial & business training prior to credit management, and appropriate for each stage. |

1. **WINGS**

| **Programme/Project Name** | Women’s Income Generating Support (**WINGS**) |
| --- | --- |
| **Paper(s)** | Green EP, Blattman C, Jamison J, Annan J. Women's entrepreneurship and intimate partner violence: A cluster randomized trial of microenterprise assistance and partner participation in post-conflict Uganda (SSM-D-14-01580R1). Social science & medicine. 2015;133:177-88. |
| **Implementing Partners** | Association of Volunteers in International Service (AVSI) |
| **WHAT?**  **Stated aim**  **Type of HIV-sensitive social protection** | To investigate impact skills and cash transfer (microgrant) programme on IPV among extremely poor and marginalized youth (mainly young women), with and without engaging male household members.  Livelihood or employability aspect: business training, micro grant, mentorship  HIV-link: risky sex behaviours: sexual initiation, unprotected sex, multiple partners |
| **Design** | **2 Cluster Randomized Pragmatic Superiority Trials**:  1: CRCT skills and cash transfer (WINGS vs. wait-list control)  2: wait-list group was randomized to WINGS+ (involving male household members) or standard programme |
| **WHEN?** | 2009-2011 (programme duration phase 1: 20 months; phase 2: 12 months)  Baseline: April-June 2009; after 16 months receiving cash grants (=/- 20 months) FOLLOW-UP survey; after further randomization for W+, endline survey June-August 2012 (1 year). |
| **WHO?**  **Sample description Inclusion criteria** | Purposive selection of 120 villages in 6 sub-counties (60 intervention vs. 60 control); 10-17 ultra-poor women per village.  1: Extremely poor and marginalized young adults (86% women); aged 14-30 (27.3 Mn age); 48% were cohabiting; average work (past month): 15.4 hours/week ($4.47) –mainly farming.  Total n=1800 participants in 60 villages randomized (via public lottery) for immediate (n=896) and 60 for delayed programme implementation (n=904); ITT women: 1488/1546 (93% retention)  2: Subsequently, the delayed group was randomized to the standard programme (n-439) and W+ (n=465). The intervention group was then further randomized to receive 0, 2 or 5 follow-up visits; ITT women: 386/400 (97% retention) |
| **WHERE?**  **Country & Context** | **Uganda** –Gulu & Kitgum districts –Northern Uganda after civil war government and the Lord of Resistance Army. In 2009, 2 million displaced people had returned home, leaving most without human or financial capital to pursue non-agricultural IGA. Villages ranged from 350-1000 people, located, on average, 45 km from district capital. |
| **HOW?**  **Description HIV-sensitive social protection intervention** | This study built on Blattman 2014 who reported that the AVSI microenterprise programme led to large increases in employment and earnings. It is a poverty alleviation programme for women empowerment.  In the current study, **phase 1** offered cash to encourage ultra-poor women to start non-farm businesses.  WINGS: 4 days business skills training (business plan, budget, marketing, financial management). Afterwards: written business plans with a start-up grant ($150; disbursed in 2 instalments), and follow-up support by AVSI field staff (every 6 weeks for monitoring & advice).  Businesses: crop sales, animal rearing, non-farm self-employment (petty trading) casual labour.  **Phase 2**: the regular economic intervention became control and intervention became *Women Plus* (W+): women and a male household member (mostly intimate partner but also father or brother) participated in training and follow-up visits together. On addition to WINGS business training, they received a 1-day training covering topics on cultural, gender, financial barriers to female entrepreneurship; couples communications & joint problem solving. Cash was transferred to women in both variants but framing in W+ shifted to collaborative household decision-making.  Hypotheses: (1) more inclusive approach would increase (in)direct (emotional) support for/relax constraints on women; (2) increased economic security & communications, collaboration will reduce tensions, hence IPV; (3) approach will create new gendered behaviour patterns + increase business success and improved relationship quality will change (wo)men’s gender attitudes. |
| **Outcomes of interest** | - IPV & gender attitudes (women’s rights and wife beating)  - Quality relationship (communications patterns, listening skills, dispute frequency etc.) partner support in household (chores)  - Autonomy (spending, household decision-making, buying clothes without permission, have a say in large purchases)  - Economic impact: (1) income (a. cash earnings; b. consumption; c. durable assets), (2) employment hours; (3) (access to) financial assets (savings, loans, credit). |
| **Outcomes** | **Phase 1**: produced large *economic impacts* (near) doubling of: microenterprise ownership from 40% to 79%; non-agricultural hours (from 5.2 to 10.1 hours); monthly earning (from $7.15 to $15.25).  Most women invested in petty trade/retail in addition to existing farming. Around a third of the grant was invested in business during the 1^st^ month; the rest was largely saved (in cash or in the form of durable assets). Counter to expectation: women chores increased with an average of 5.8. Those suffering from IPV earned $18.12/month less (wiping out intervention effect), but they increased their durable assets and consumption instead.  *IPV/relationship/gender norms impacts*: there were significant increases in marital control (having to give $ to partner/$ taken against will) and quality of the relationship. There was no effect on any form of IPV. There was a non-significant increase in women’s positive gender norms/autonomy.  **Phase 2**: showed little impact of W+ on *economic* outcomes: involving males did not cause different spending of grant. It decreased the proportion of women in business by 9 percentage points; increased women belonging to savings group by 6 percentage points; decreased consumption (0.31 standard deviation (sd)).  The largest and most significant results on relationships: increased quality (0.23sd); more support in business and even for ‘female’ chores. Effects were larger for those with intimate partners before starting W+.  *IPV/relationship/gender norms impacts*: non-significant decrease of marital control, IPV. The only significant result: a decrease in limiting partner contact with family & friends (0.11sd). Per protocol analyses produced similar results. Unchanged gender attitudes and women in W+ had slightly less autonomy (0.07sd).  **Conclusion**: economic success did not reduce IPV; male involvement did not increase business success, reduce IPV, but relationship quality could improve. |
| **Comments** | There was moderate baseline imbalance for Phase 1: Intervention group was slightly worse off, which could lead to an underestimation of the intervention effects. There was high retention in both phases; among W+: 100% compliance among women; attrition was mainly due to partner noncompliance. Data was self-reported. IPV could be underreported.  Reasons why, despite doubling of income, there was no reduced IPV: (1) male backlash/women’s bargaining power only weakly linked with IPV; (2) they cancelled each other out; (3) larger changes may be required (despite doubling, clients remained poor in absolute terms); (4) conflict-affected population are perhaps more vulnerable; (5) 1-day gender training is a small dose; (6) men’s co-ownership could both decrease women’s autonomy & improve cooperative behaviour.  Nonetheless, study indicated that partner relationship is an important determinant for economic success.  Need to study change mechanisms of how economic factors affect partner relationships to get a better insight in when to engage men in women’s empowerment. |

1. **WORTH**

| **Programme/Project Name** | **WORTH+** (is name, not acronym) |
| --- | --- |
| **Paper(s)** | Pettifor A, Wamoyi J, Balvanz P, Gichane MW, Maman S. Cash plus: exploring the mechanisms through which a cash transfer plus financial education programmeme in Tanzania reduced HIV risk for adolescent girls and young women. Journal of the International AIDS Society. 2019;22:e25316. |
| **Implementing Partners** | Sauti Project (USAID funded - Determined, Resilient, Empowered, AIDS-free, Mentored and Safe women (DREAMS) |
| **WHAT?**  **Stated aim**  **Type of HIV-sensitive social protection** | To explore perceived effects combined cash transfer (CT)/behaviour change communication (BCC) and MFI programme on risky sex among out-of-school young women  Livelihood or employability aspect: cash transfer + financial education, business development, mentorship, savings, loans  HIV-link: risky sexual behaviours: transactional, intergenerational and safe sex |
| **Design** | **Qualitative research**: 60 IDI; 20 narrative timeline interviews (indicating times cash transfers (CT) & sexual partners past 2 years + partner characteristics (financial support, condom use, HIV test, overall quality relationship) |
| **WHEN?** | June 2017-July 2018  20 baseline interviews June 2017 (after first CT); 20 follow-up interviews in June 2018; 40 new IDI.  20 narrative timeline interviews |
| **WHO?**  **Sample description Inclusion criteria** | N=80 Out-of-school, 15-23-year-old AGYW. There were nearly equal numbers of 15-19 years old & 20-23-years old to purposively sample more unmarried young women (65%) |
| **WHERE?**  **Country & Context** | **Tanzania** –rural area: Bulungwe is most rural; Shinyanga is district headquarters; Kahama experienced rapid population growth due to gold mine. All three locations are at 3 hours from each other. |
| **HOW?**  **Description HIV-sensitive social protection intervention** | AGYW who had attended at least 10 hours of BCC were eligible to receive CT ($31/3 months) for a duration of 18 months. They were offered WORTH+, an MFI programme with financial literacy, individual savings and loan programme. |
| **Outcomes of interest** | Experiences, perspectives, reported behaviours, especially partner choice and transactional sex |
| **Outcomes** | Programme design played a key role in programme effects: young women internalized stated aim of the CT to develop businesses, earn money and become less dependent on men.  Cash helped meet basic needs (food, clothes, sanitary pads, soap) for the most vulnerable (poorest) AGYW. The better off were able to develop/expand business, attend training, and some even received help from family, like a sewing machine.  Cash reduced transactional sex among poorer women (basic needs were covered by CT), who were mostly unmarried. They purchased small personal items with CT (sanitary pads, lotion, oil).  Entrepreneurial skills enhanced future aspirations like going for job training and/or buying assets (livestock/land/sewing machine) to secure income beyond the CT time period.  *Social support* (family & programme mentors) enhanced entrepreneurial success in the form of advice to stay on track with business/financial goals. Male partners were perceived as supportive when married, as cash was used for the household. Unmarried women preferred not telling partners (relationships were not serious enough, they worried to lose financial support or there was a lack of trust). AGYW in savings group asked money of group members rather than men (transactional sex) & had programme mentors to help keep them focused on business development. Also, by not having to ask parents/partner for cash, there was less tension/potential violence & improved relationship quality.  Regarding abstinence & refusal unwanted sex: self-esteem gave them agency to say no. Younger women could abstain and sexually active women could refuse partners, reducing partner numbers. There was little talk of the effect of CT on condom use/ HIV testing. |
| **Comments** | CT was especially effective in reducing transactional sex for basic needs. It is less likely that such small transfers would affect other motivations for transactional sex (social status & material expressions of love). Increased agency and self-esteem had likely a greater effect on HIV risk reduction: business development, money management contributed to greater self-confidence, courage to try new things, decision-making, aspirations and future orientation.  Future programmes should include more training on future goals and aspirations. |

1. **ZOE**

| **Programme/Project Name** | **ZOE** Orphan empowerment |
| --- | --- |
| **Paper(s)** | Goodman ML, Selwyn BJ, Morgan RO, Lloyd LE, Mwongera M, Gitari S, et al. Sexual behavior among young carers in the context of a Kenyan Empowerment Programme Combining Cash-Transfer, Psychosocial Support, and Entrepreneurship. The Journal of Sex Research. 2016;53(3):331-45. |
| **Implementing Partners** | ZOE Orphan empowerment  Kenyan AIDS Control Council (ABC prevention training of trainers -training of trainers (ToT))  Mission for essential drugs and supplies (education on VCT) |
| **WHAT?**  **Stated aim**  **Type of HIV-sensitive social protection** | To reduce HIV risk in orphans and vulnerable children (OVC) through a comprehensive economic empowerment and HIV prevention programme.  Livelihood or employability aspect: CT, microenterprise development, psychosocial support, life skills  HIV-link: risky sex behaviours: sexual initiation, unprotected sex, multiple partners |
| **Design** | Analytical cross-sectional: 1060 structured questionnaires March 2014 |
| **WHEN?** | 2012-2014 (programme duration 3-years; new cohort enrolled every February) |
| **WHO?**  **Sample description Inclusion criteria** | Most at risk households of OVC siblings were identified by local leaders & programme social workers based on orphan status, food security, household economic strength at baseline; eldest sibling had caring responsibilities (for younger siblings, older relatives, or terminally ill parents).  There were 64 working groups with young carers and a community mentor: total n=1060  Cohort 1: n= 359 (female: 223; Mn age 18); joined prior to data collection =control  Cohort 2: n=446; (female 321; Mn age 18); 1-year exposure  Cohort 3: n=255; (female 162; Mn age 19.5); 2 years exposure |
| **WHERE?**  **Country & Context** | **Kenya** –Meru County; 13 programme regions |
| **HOW?**  **Description HIV-sensitive social protection intervention** | Working groups elected their own leaders; held weekly meetings and monthly regional meetings. They received sex education training; shared personal stories and provided peer support.  *Economic empowerment*: They received micro grants in a joint bank account; decided on entrepreneurial endeavours/training; decided on financial products by majority vote. Entrepreneurial training: general business management & vocational skills (barbering, tailoring, car mechanics), often with start-up kits (hardware to start business –e.g., sewing machine, tools etc.). Financial products: permanent revolving funds for loans/emergency cash, short-term payments; longer-term education-related/business payments. Material inputs depended on needs/interests of families in workshops.  *Sexual behaviour change*: ABC training+ VCT: ToT to community & workshop leaders who trained workshops. The Mission provided for essential drugs and supplies for VCT. |
| **Outcomes of interest** | Predictor variables: time in programme; age; orphan household factors (size, years since parent had died); food consumption/security; psychological assets (self-efficacy, resilience); education (years & basic literacy); material inputs programme/personal monthly income.  Outcome measures: sexual initiation in the past 6 months; unprotected sex / multiple sex partners in the past year. |
| **Outcomes** | Cohort 3 girls had received more material inputs (cash & kits) but monthly income ($12.05 was the same as for Cohort 2 girls, who in turn had twice the income compared with Cohort 1 ($6.02); higher food security; more condom use than Cohort 1;  *Sexual initiation*: Cohort 3 had 3x the odds vs Cohort 1 (increased 50% by each added year of age) –but this was not associated with material inputs/monthly income. Being a year older at the time of parental death was associated with 2% reduction of sexual initiation odds. Improved food consumption was associated with increased odds of sexual initiation. Mental health was associated with sexual initiation: 1 point increase self-efficacy: 8% reduction odds; one-point resilience: 2% increase.  *Unprotected sex*: programme participation was associated with reduced odds of unprotected sex: Cohort 1 had 3x the odds of Cohort 3; material inputs & monthly income were unassociated. Better food was associated with higher odds: those with borderline/acceptable food consumption had 3x higher odds than females with poor food consumption. Other associations: being older increased odds; more years passed since parental death decreased odds; increase of self-efficacy by 1 point was associated with 11% reduced odds of unprotected sex; 1 added school year reduced odds by13%.  *Multiple sex partners*: programme participation, age, years since parental death were not associated; improved food consumption increased odds; 1-point increase of self-efficacy: 8% decrease odds; one-point increase in resilience: 3% increase odds.  **Conclusion**: Although overall programme participation seemed protective against sexual initiation/unprotected sex; material transfers and increased monthly income were largely unassociated with risky sex behaviour. |
| **Comments** | Self-efficacy was protective across three outcomes which may indicated that programme design offered unmeasured psychosocial support.  A possible explanation for increasing sexual risk behaviour among more resilient carers could be linked to an increased ability to meet need for support/intimacy.  Correlation increased food consumption and increased risky sex: could be reverse causation –transactional sex? Girls more desirable? There is a need for longitudinal and qualitative data to find that out.  Outcomes were quite limited: there was no information on economic outcomes, biological data (STI, pregnancy), on age discordance, or school attendance.  The amount of the cash transfer was not reported. |

**Additional File 3. Mentors and Safe Spaces -data extraction**

| **Project** | **Mentor** | **Safe space** |
| --- | --- | --- |
| AGEP | Female mentors from the community aged 25-35 years.  10-day initial training & 5-day FU training | Social safe space through weekly group meetings in which 15-25 girls meet for 30-90 min to (1) build a platform for info dissemination (H & financial); (2) to build social assets (friends, trusting relationships and self-efficacy) |
| Asset | Graduates from Binti run girl groups. They receive stipends. | Physical safe space (Binti Pamoja Centre) for youth development programme offering health education and professional and life skills. |
| ELA Uganda  NB 2018 paper | Slightly older female mentor selected from the community.  They received 1-week initial training and monthly refresher courses. They received small stipend.  Rationale behind ‘close in age’ helps to transfer knowledge (Ray 2006) -youth uncomfortable to discuss issues with teachers (Gallant 2004; Ross 2006) | Designated adolescent development clubs that are fixed meeting places in communities rather than in schools to reach dropouts (BRAC model).  For girls 14-20 years to learn life skills and vocational training. Clubs are a protected local space where girls meet, socialize and develop skills, free from pressures by (older) men. They are open 5 afternoons per week to not interfere with school attendance but provide a safe place for the time parents have not yet returned from work. Clubs host recreational activities: reading, staging plays, singing, dancing, games.  After 2 years no more training, only social safe space, but sustained reduced sexual risk behaviour. |
| ELA Tanzania | Adolescent leader recruited from the community conducts training and facilitated club activities, 1-week residential training on club maintenance and TOT for life skills component. Monthly stipend was US$15.  There was inadequate training of new mentors; each month 5 out of 20-30 mentors would drop out and then it took long before new mentors were trained. There were few supervisory visits | Safe space to share experiences. Usually set up in a 1-room house at an easy-to-reach location and equipped with books and equipment for in-and outdoor games. Set up to attract girls with recreational activities and then leverage interest to conduct skills training.  Increased participation in informal savings groups is likely spillover effect of social interaction/social networks.  Problem was that BRAC had little control over donated space (public spaces like school or church or donated house that were often too small for 20-30 girls), hence ability to plan club activities + spaces were often insecure. It does not qualify ‘insecure’. |
| IMAGE |  | Social safe space through biweekly loan repayment meetings and training using the participatory curriculum ‘Sisters for Life’. Integrated in loan centre, meetings are for group-based learning and to foster solidarity and collective action. |
| SHAZ! 2010 | Mentors were successful businesswomen identified through community outreach. They agreed to regularly meet to offer business-related support. Lack of structured and regular meetings hampered support resulting in a lack of trust between mentors and participants with mentors lacking time and mentees lacking money for transport. Accusation of the other wanting free labour/not wanting to work for money.  Exception: girls found very ‘good aunts’ in life skills trainers. | Social safe place through weekly microcredit meetings.  HIV education and life skills offered. They used Stepping Stones curriculum with gender, communications and relationship skills. |
| SHAZ! 2014 | Self-selected adult mentors but no reporting of results associated with mentors. | Social safe space through life and financial skills training with social support element. There are 25 participants per group who attend 14 modules over four to six weeks. |
| SS&CF | Training given by slightly older peers who received mentor training. | Social safe space through group meetings for gender transformative and participatory livelihood development programme, which leveraged critical dialogue and reflection for livelihoods.  Single sex groups with 20 participants. Three-hour meetings for 12 weeks. |
| TRY 2005  NB 2006 has extensive mentor description | Part-time adult mentors with counselling, social work, business, health care and community development background recruited by the NGO Population Council on part-time contracts ($104/month ± salary teacher). They received 4 (mentors) or 5 (credit officers) days training on team building, comms, gender issues, SRH/HIV and life skills. After needs assessment, they organized group discussions, recreation, excursions, sports and fitness. Sometimes education seminars. They occurred during credit meetings or at other times.  Nine mentors were grouped into 3 teams: (1) business; (2) public health; (3) social work/community development.  Mentors offered advice and counselling.  For credit officers it became difficult to be both mentor and the person admonishing girls to repay loans.  When TRY moved to separating the two roles, it found focusing on mentors for social support overlooked the girls’ saving needs. That’s when they moved to the Young Savers Club.  Social support mechanisms: encourage mutual support and develop reciprocal relationships. The opportunity to make new friends and create a wider social network also contribute to development of new business and social contacts.  Mentors were much appreciated by girls, helped boost attendance and create social cohesion. High turnover. Older, more financially stable remained more stably with TRY. | Weekly 2-hour meetings with credit officers in community social halls or church meeting rooms. Groups of 20-25 girls. The group meetings also became social events in which girls shared intimate experiences of their lives and trouble.  Young Savers Club: members form the groups themselves. Groups are led by either credit officer or mentor. Clubs meet weekly and engage with mentors and participate in recreational activities (sports/games). Girls appreciate a safe place to save (so boyfriends would not know they had money) and the friendships they form.  “In the group, problems—even individual problems—are less troublesome  when we share them.—Age 20, married, one child, eight y. of education  Merry-go-rounds: spontaneous establishment from the start. Some groups would congregate in recipients’ house to socialize with tea and snacks.  The value girls place on group membership was clear from qual findings in 2006 publication. For most girls the groups were their only source of social contact and support, translating to support with medical aid, baby showers.  Since I joined the group, I have acquired a number of friends with whom I share problems or with whom I just have fun. I don’t feel lonely.  —Focus-group participant, age 22, divorced, eight years of education  Meeting venues were often rented and time in venues was constrained. Often credit meetings were given priority over mentor meetings. |
| WINGS | The follow-up support by trained field staff after initial business training to support clients with advice and monitor spending every 6 weeks. No results reported re this specific support. | Not mentioned. |
| WORTH+ | Programme mentors lead small savings/loan groups, helping young women to set up businesses and stay on track. Social support from programme mentors (and family support) enhanced young women’s success. They could help transition AGYW from adolescence into adulthood with training in key job and life skills.  Authors mention future programmes could benefit from strengthening the mentorship component and focus more on future goals and aspirations. They call enhancing CT with mentorship, financial education and health services cash plus programmes. | Social safe place in savings groups after having attended 10 hours of behaviour change communication training. Girls and young women would ask money from members in savings groups, rather than from parents or boyfriends, which improved relationship quality, and led to less tension and less risk of potential violence. |
| ZOE | Use of community mentor in working groups with weekly meeting and monthly regional meetings, but not elaborated on, not any outcomes reported for mentoring. | Social safe space through weekly meetings and monthly regional meetings. They receive sex education, share personal stories and offer and receive peer support. |

**Additional File 4. Table of excluded full text papers with reasons for exclusion**

| **Nr** | **First author name & year** | **Title** | **Reason for exclusion** |
| --- | --- | --- | --- |
| 1 | Abebe & Skovdal 2010 | Livelihoods, care and familial relations of orphans eastern Africa | Not intervention of interest |
| 2 | Adato, Hoddinott & Haddad 2005 | Power, politics, and performance: Community participation in South African public works program | Not intervention of interest: community participation is intervention, not public works |
| 3 | Adato & Basset 2009 | Social protection to support vulnerable children and families: The potential of cash transfers to protect education, health and nutrition | Excluded publication type: Review |
| 4 | Adato, Devereux & Sabates-Wheeler 2016 | Accessing the "right" kinds of material and symbolic capital: the role of cash transfers in reducing adolescent school absence and risky behaviour South Africa | Not intervention of interest (UCT) |
| 5 | Agadjanian & Sen 2007 | Promises and challenges of faith-based AIDS care and support Mozambique | Wrong population: faith-based institutions |
| 6 | Akanle et al 2018 | Turbulent but I must endure in silence: female breadwinners Nigeria | Wrong country: Nigeria |
| 7 | Ama et al 2014 | Exploring challenges facing women entrepreneurs in informal cross-border trade in Botswana | No HIV-related outcomes |
| 8 | Angeles et al 2019 | Government of Malawi's unconditional cash transfer improves youth mental health | Not intervention of interest (UCT) |
| 9 | Austrian & Muthengi 2014 | Can economic assets increase girls' risk of sexual harassment? Evaluation results from social, health and economic asset-building intervention for vulnerable adolescent girls in Uganda | Wrong outcome of interest: this was verbal abuse (teasing), not IPV |
| 10 | Ayuku et al 2015 | The government of Kenya cash transfer for orphaned and vulnerable children: cross-sectional comparison of household and individual characteristics of those with & without | Not intervention of interest (UCT); no gender stratification |
| 11 | Baird et al 2010 | The short-term impacts of a schooling conditional cash transfer program on the sexual behaviour of young women | Not intervention of interest (focus on return to school and school attendance) |
| 12 | Baird 2011 | Cash or condition? Evidence from cash transfer experiment | Wrong population: Baseline dropouts were excluded |
| 13 | Baird & Ozler 2012 | Examining the reliability of self-reported data on school participation | Excluded publication type: Method paper |
| 14 | Baird et al 2012 | Effect of a cash transfer programme for schooling on prevalence of HIV and Herpes simplex type 2 in Malawi: a cluster randomised trial | Not intervention of interest (focus on return to school and school attendance) |
| 15 | Baird et al 2013 | Income shocks and adolescent mental health | No HIV-related outcomes (mental health) |
| 16 | Baird et al 2018 | Can interventions to increase schooling and incomes reduce HIV incidence among young women in sub-Saharan Africa? | Excluded publication type: Review |
| 17 | Balfour et al 2013 | HIV prevention in action on the football field: The Whizzkids United Program South Africa | Wrong population: kids too young; Not intervention of interest |
| 18 | Barcucci, Zanola, Axmann 2017 | Vocational education and training (VET) and the transition of young women and men to the labour market in middle-income countries: A comparative analysis based on ILO surveys in Jamaica, Jordan, Peru, Tunisia, Ukraine, Vietnam and Zambia | No HIV-related outcomes |
| 19 | Beauclair et al 2018 | Partner age differences and associated sexual risk behaviours among adolescent girls and young women in a cash transfer programme for schooling in Malawi | Wrong population: no baseline dropouts |
| 20 | Bell et al 2008 | Building protective factors to offset sexually risky behaviours among black youth: a randomized controlled trial | Wrong population: kids too young (9-13 years) |
| 21 | Berejena 2017 | Preparing for successful transitions beyond institutional care in Zimbabwe: adolescent girls' perspectives and programme needs | Excluded publication type: Not an empirical study |
| 22 | Blattman 2013 | Generating skilled self-employment in developing countries: experimental evidence from Uganda | No HIV-related outcomes |
| 23 | Brinbaum, Guegnard 2012 | Education and training pathways and transition to work among young people from immigrant families seen from the angle of guidance | Wrong country: France |
| 24 | Bukuluki et al 2019 | Facilitating transition of adolescent and emerging adults from care into employment: case study Uganda Youth Development Link | No HIV-related outcomes |
| 25 | Campbell et al 2014 | Community resistance to a peer education programme in Zimbabwe | Wrong population: female sex workers |
| 26 | Carlson et al 2012 | Enhancing ado self-efficacy & collective efficacy through public engagement around HIV/AIDS competence: a multilevel cluster randomized-controlled trial | Wrong population: too young (9-14 years) |
| 27 | Cho et al 2011 | Keeping adolescent orphans in school to prevent HIV infection: evidence from a randomized controlled trial in Kenya | Wrong population: too young (12-14 years) |
| 28 | Cho et al 2017 | A school support intervention and educational outcomes among orphaned adolescents: results of a cluster randomized controlled trial in Kenya | Wrong population: no baseline dropouts |
| 29 | Cho et al 2018 | School support as structural HIV prevention for adolescent orphans in Western Kenya | Wrong population: no baseline dropouts |
| 30 | Cluver et al 2013 | Child-focused state cash transfers and adolescent risk of HIV infection in South Africa: a propensity score-matched case-control study | Not intervention of interest (UCT) |
| 31 | Cluver et al 2014 | Cash plus care: social protection cumulatively mitigates HIV-risk behaviour among adolescents in South Africa | Wrong population Girls too young: baseline age 14.3 |
| 32 | Cluver et al 2016 | Structural drivers and social protection: mechanism HIV risk and HIV prevention for South African adolescents | Wrong population Girls too young: baseline age 14.3 |
| 33 | Cluver et al 2016 | Can social protection improve sustainable development goals for adolescent health? | Wrong population Girls too young: baseline age 14.3 |
| 34 | Cluver et al 2016 | Combination social protection for reducing HIV-risk behaviour among ado in South Africa | Wrong population Girls too young: baseline age 14.3 |
| 35 | Cluver et al 2019 | Improving lives by accelerating progress towards UN sustainable development goals for adolescents living with HIV: a prospective cohort study | Wrong population Girls too young: baseline age 13.78 |
| 36 | Corbett-Ondiek 2016 | Peering into "spaces for change": empowerment, subversion and resistance in gendered violence prevention education programme in Kenya | Wrong population: facilitators, not young women |
| 37 | Cockcroft et al 2018 | Access of choice-disabled young women in Botswana to government structural support programmes: a cross-sectional study | Describes characteristics young women rather than programme outcomes |
| 38 | Dageid & Duckert 2007 | The process of evaluating a capacity-building support initiative for HIV positive South Africans | Wrong population: not for young women (31.7 & 35.6 years old) |
| 39 | Daniels 2007 | Improving health, improving lives: impact of the African Youth Alliance and new opportunities for programmes | Excluded publication type: Commentary with some results in ++ rather than in numbers |
| 40 | Daum 2019 | Of bulls and bulbs: aspirations, opinions and perceptions rural ado and youth in Zambia | Wrong topic of interest: to get deeper understanding of aspirations rural youth; no HIV-related outcomes |
| 41 | Delva et al 2010 | HIV Prevention through sport: case of Mathare Youth Sport Association in Kenya | Not intervention of interest (sports membership). |
| 42 | Dladla & Mutambara 2018 | The impact of training and support interventions on small businesses in the Expanded Public Works programme-Pretoria region | Wrong population: women too old |
| 43 | Draper & Coalter 2016 | 'There's just something about this club. It's been my family'. Sport for development programme | Wrong population: males |
| 44 | Embleton et al 2019 | Adapting evidence-based gender, livelihoods & HIV prevention intervention with street-connected young people in Eldoret, Kenya | Excluded publication type: Method paper |
| 45 | Evans 2012 | Safeguarding inheritance and enhancing resilience of orphaned young people living in child- & youth-headed households in Tanzania and Uganda | Not intervention of interest |
| 46 | Fernald et al 2008 | Small individual loans and mental health: a randomized controlled trial among South African adults | Wrong population: women too old (36 years) |
| 47 | Fieno, Leclerc-Madlala 2014 | The promise and limitations of cash transfer programs for HIV prevention | Social protection intervention not intervention of interest: focus on cost simulation, not intervention |
| 48 | Galarraga et al 2018 | The empower nudge lottery to increase dual protection use: a proof-of-concept randomised controlled trial in South Africa | Not intervention of interest |
| 49 | Gnauck et al 2013 | Economic empowerment and AIDS-related stigma in rural Kenya: A double-edged sword? | Wrong population: women too old (median age 40 years) |
| 50 | Hajdu et al 2013 | Rural young people's opportunities for employment and entrepreneurship in globalised southern Africa: the limitations of targeting policies | Not intervention of interest |
| 51 | Hallfors et al 2012 | Process and outcome evaluation of a community intervention for orphan adolescents in Western Kenya | Wrong population: too young (12.9 years); schoolgirls |
| 52 | Hallfors et al 2015 | The Impact of School Subsidies on HIV-Related Outcomes Among Adolescent Female Orphans | Wrong population: too young (12 years) |
| 53 | Hallfors et al 2017 | Process Evaluation of a Clinical Trial to Test School Support as HIV Prevention Among Orphaned Adolescents in Western Kenya | Wrong population: too young (14.8 years) |
| 54 | Han et al 2013 | Family economic empowerment and mental health among AIDS-affected children living in AIDS-impacted Communities: evidence from a randomised controlled trial in southwestern Uganda | Wrong population: too young (12-14 years) |
| 55 | Hanass-Hancock 2014 | Tangible skill building and HIV youth prevention intervention in rural South Africa | Wrong population: too young (13.4 years) |
| 56 | Handa et al 2014 | The government of Kenya cash transfer program reduces risk of sexual debut among young people 15-25 | Not intervention of interest (UCT) |
| 57 | Handa et al 2015 | Impact of the Kenya Cash Transfer for orphans and vulnerable children on early pregnancy and marriage of adolescent girls | Not intervention of interest (UCT) |
| 58 | Handa et al 2017 | How does a national poverty programme influence sexual debut among Kenyan adolescents? | Not intervention of interest (UCT) |
| 59 | Heinrich & Brill 2015 | Stopped in the Name of the Law: Administrative Burden and its Implications for Cash Transfer Program Effectiveness | Not intervention of interest (UCT) |
| 60 | Heinrich et al 2017 | Reducing Adolescent Risky Behaviours in a High-Risk Context: The Effects of Unconditional Cash Transfers in South Africa | Not intervention of interest (UCT) |
| 61 | Hershow et al 2015 | Using soccer to build confidence and increase HCT uptake among adolescent girls: A MM study of an HIV prevention programme in South Africa | Wrong population: too young (14.2 years) |
| 62 | Hjelm et al 2017 | Poverty and perceived stress: Evidence from two unconditional cash transfer programs in Zambia | Not intervention of interest (UCT) |
| 63 | Iritani et al 2016 | Educational Outcomes for Orphan Girls in Rural Zimbabwe: Effects of a School Support Intervention | Wrong population: too young (12 years) |
| 64 | Ismayilova et al 2012 | Imagining the future: Community perceptions of a family-based economic empowerment intervention for AIDS-orphaned adolescents in Uganda | Wrong population: too young (13.8 years); schoolgirls |
| 65 | Ismayilova et al 2012 | Family Support as a Mediator of Change in Sexual Risk-Taking Attitudes Among Orphaned Adolescents in Rural Uganda | Wrong population: too young (13.7 years) |
| 66 | James et al 2006 | The Impact of an HIV and AIDS Life Skills Program on Secondary School Students in KwaZulu-Natal, South Africa | Not intervention of interest |
| 67 | Jennings et al 2016 | Effect of savings-led economic empowerment on HIV preventive practices among orphaned adolescents in rural Uganda: results from the Suubi-Maka randomized experiment | Wrong population: too young (13.4 years) |
| 68 | Jewkes et al 2008 | Impact of Stepping Stones on incidence of HIV and HSV-2 and sexual behaviour in rural South Africa: cluster randomised controlled trial | Not intervention of interest |
| 69 | Jewkes et al 2012 | Motivations for, and perceptions and experiences of participating in, a cluster randomised controlled trial of a HIV-behavioural intervention in rural South Africa | Not intervention of interest |
| 70 | Jiyane & Zawada 2013 | Sustaining Informal Sector Women Entrepreneurs through Financial Literacy | Wrong population: women too old (35-60 years) |
| 71 | Junior et al 2016 | The Perspectives of Young Women in Rural Western Kenya on Unconditional Cash Transfers | Not intervention of interest (UCT) |
| 72 | Kagotho et al 2012 | Correlates of depression among caregivers of children affected by HIV/AIDS in Uganda: Findings from the Suubi-Maka Family Study | Wrong population: too young caregivers too old -43.4 years |
| 73 | Kagotho et al 2018 | Assessing the Association Between Depression and Savings for Kenyan Youth Using a Validated Child Depression Inventory Measure | Wrong population: too young (13.5 years) |
| 74 | Kakuru 2008 | Rural livelihoods, HIV/AIDS and women's activism: The struggle for gender equality in primary education in Uganda | Excluded publication type: Editorial |
| 75 | Karamagi et al 2018 | Quality improvement as a framework for behavio0r change interventions in HIV-predisposed Communities: a case of AGYW in northern Uganda | Not intervention of interest |
| 76 | Karimli & Ssewamala 2015 | Do Savings Mediate Changes in Adolescents' Future Orientation and Health-Related Outcomes? Findings From a Randomized Experiment in Uganda | Wrong population: too young (13 years) |
| 77 | Kassile et al 2014 | Health and social support services to HIV/AIDS infected individuals in Tanzania: Employees and employers’ perceptions | Not intervention of interest |
| 78 | Kennedy et al 2014 | Exploring the potential of a conditional cash transfer intervention to reduce HIV risk among young women in Iringa, Tanzania | Excluded publication type: Feasibility study about hypothetical CCT. |
| 79 | Khoza et al 2018 | Men's perspectives on the impact of female-directed cash transfers on gender relations: Findings from the HPTN 068 qualitative study | Wrong population: schoolgirls |
| 80 | Khoza et al 2018 | Cash transfer interventions for sexual health: meanings and experiences of adolescent males and females in inner-city Johannesburg | Not intervention of interest |
| 81 | Kilburn et al 2016 | Effects of a large-scale unconditional cash transfer program on mental health outcomes of young people in Kenya | Not intervention of interest (UCT) |
| 82 | Kilburn et al 2018 | CCT & the reduction in partner violence for young women: an investigation of causal pathways using evidence from a randomized experiment in South Africa (HPTN 068) | Wrong population: schoolgirls |
| 83 | Kilburn et al 2019 | Cash Transfers, Young Women's Economic Well-Being, and HIV Risk: Evidence from HPTN 068 | Wrong population: schoolgirls |
| 84 | Kim et al 2007 | Understanding the impact of a microfinance-based intervention on women's empowerment and the reduction of intimate partner violence in South Africa | Wrong population: no baseline dropouts |
| 85 | Kim et al 2016 | Short-Term Impacts of a Cash Transfer Program for Girls' Education on Academic Outcomes: Evidence from a Randomized Evaluation in Malawian Secondary Schools | Wrong population: women too old: mean age 41 years -population from IMAGE |
| 86 | Kirsten 2012 | Microfinance and women's well-being: Evidence from South Africa | Wrong population: women too old - population from IMAGE |
| 87 | Kivumbi et al 2019 | Utilizing a family-based economic strengthening intervention to improve mental health wellbeing among female adolescent orphans in Uganda | Wrong population: too young (12.7 years); schoolgirls |
| 88 | Koski et al 2018 | The impact of eliminating primary school tuition fees on child marriage in sub-Saharan Africa: A quasi-experimental evaluation of policy changes in 8 countries | Not intervention of interest |
| 89 | Koyana & Mason 2017 | Rural entrepreneurship and transformation: the role of learnerships | No HIV-related outcomes |
| 90 | Kwiringira et al 2019 | Livelihood Risk, Culture, and the HIV Interface: Evidence from Lakeshore Border Communities in Buliisa District, Uganda | Not intervention of interest |
| 91 | Langevang & Gough 2012 | Diverging pathways: young female employment and entrepreneurship in sub-Saharan Africa | Wrong country: Ghana |
| 92 | Larson et al 2013 | Exploring impacts of multi-year, community-based care programs for orphans and vulnerable children: A case study from Kenya | Data issues: no gender-stratified data |
| 93 | Lu et al 2013 | Assessing barriers and facilitators of implementing an integrated HIV prevention and property rights program in Western Kenya | Not intervention of interest |
| 94 | Luseno et al 2013 | A multilevel analysis of the effect of Malawi's Social Cash Transfer Pilot Scheme on school-age children's health | Not intervention of interest (UCT) |
| 95 | Luseno et al 2015 | HIV infection and related risk behaviours: does school support level the playing field between orphans and nonorphans in Zimbabwe? | Wrong population: schoolgirls |
| 96 | Lusinje et al 2015 | Youth clubs’ contributions towards promotion of sexual and reproductive health services in Machinga district, Malawi | Not intervention of interest |
| 97 | MacPhail et al 2013 | Acceptability and Feasibility of Cash Transfers for HIV Prevention Among Adolescent South African Women | Wrong population: schoolgirls |
| 98 | MacPhail et al 2018 | Cash transfers for HIV prevention: what do young women spend it on? Mixed methods findings from HPTN 068 | Wrong population: schoolgirls |
| 99 | MacPherson 2015 | Exploring the complexity of microfinance and HIV in fishing communities on the shores of Lake Malawi | Wrong population: women too old (30-50 years) |
| 100 | Magnani et al 2005 | The impact of life skills education on adolescent sexual risk behaviours in KwaZulu-Natal, South Africa | Wrong population: schoolgirls |
| 101 | Makufa et al 2017 | Empowering caregivers of orphans and vulnerable children in Swaziland | Wrong population: caregivers; too old |
| 102 | Malema, Naidoo 2017 | Spaces for the empowerment of women: Rural arts and crafts projects | Wrong population: women too old -44.6 years |
| 103 | Maman et al 2016 | Leveraging strong social ties among young men in Dar es Salaam: A pilot intervention of microfinance and peer leadership for HIV and GBV prevention | Wrong population: intervention for men |
| 104 | Mantsios et al 2016 | Community Savings Groups, Financial Security, and HIV Risk Among Female Sex Workers in Iringa, Tanzania | Wrong population: female sex workers |
| 105 | Mantsios et al 2018 | 'That's how we help each other': Community savings groups, economic empowerment and HIV risk among FSW in Iringa, Tanzania | Wrong population: female sex workers |
| 106 | Meinck et al 2019 | Does free schooling affect pathways from adverse childhood experiences (ACE) via mental health distress to HIV risk among adolescent girls in South Africa: a longitudinal model pathway model | Not intervention of interest; Wrong population: too young (14.3 years) |
| 107 | Merrill et al 2018 | Linking at-risk SA girls to sexual violence and reproductive H services: A MM assessment of a soccer-based HIV prevention program and pilot SMS campaign | Not intervention of interest; Wrong population: too young (11.9 years) |
| 108 | Mohapi, Pitsoane 2017 | Life skills as a behaviour change strategy in the prevention of HIV and AIDS: Perceptions of students in an open and distance learning institution | Data issues: no gender or age stratification; no actual intervention |
| 109 | Moreki et al 2011 | Strengthening HIV/AIDS food security mitigation mechanisms through village poultry | Wrong population: too old (54.6 years) |
| 110 | Mutenje et al 2007 | Sustainable income-generating projects for HIV-affected HH in Zimbabwe: Evidence from two high-density suburbs | Data issues: age and gender not clear from text |
| 111 | O'Neill Berry 2013 | Entrepreneurial training for girls’ empowerment in Lesotho: process evaluation of a model programme | No HIV-related outcomes, only testing (is excluded) |
| 112 | Nabunya, Ssewamala 2014 | Family econ strengthening and parenting stress among caregivers of AIDS-orphaned children: Results from a CRCT in Uganda | Wrong population: caregivers; too old |
| 113 | Natali et al 2018 | Does money buy happiness? Evidence from an unconditional cash transfer in Zambia | Not intervention of interest (UCT) |
| 114 | Ngware 2016 | Moderated effects of risky behaviour on academic perform among ado girls living in urban slums of Kenya | Wrong population: schoolgirls |
| 115 | Odek et al 2009 | Effects of micro-enterprise services on HIV risk behaviour among female sex workers in Kenya's urban slums | Wrong population: female sex workers |
| 116 | Odimegwu et al 2016 | Risky sexual behaviours among women: Does economic empowerment matter? Case of Gabon, Mozambique, Sierra-Leone and Zambia | Not intervention of interest |
| 117 | Ogando et al 2017 | Gender and informal livelihoods Coping strategies and perceptions of waste pickers in Sub-Saharan Africa and Latin America | Not intervention of interest |
| 118 | Omwami et al 2011 | Effects of a school feeding intervention on school attendance rates among elementary schoolchildren rural Kenya | Wrong population: too young (7 years) |
| 119 | Patel et al 2019 | Perspectives of South African caregivers in receipt of Child Support Grants: Implications for family strengthening interventions | Not intervention of interest (UCT) |
| 120 | Peacock et al 2007 | Grassroot Soccer resiliency pilot program: building resiliency through sport-based education in Zambia and South Africa | Data issues: no gender or age stratification |
| 121 | Peterman et al 2018 | List randomization for soliciting experience of intimate partner violence: Application to the evaluation of Zambia's unconditional child grant program | Not intervention of interest (UCT) |
| 122 | Pettifor et al 2016 | The effect of a conditional cash transfer on HIV incidence in young women in rural South Africa (HPTN 068): a phase 3, randomised controlled trial | Wrong population: schoolgirls |
| 123 | Pettifor et al 2016 | HPTN 068: A RCT of a CCT to Reduce HIV Infection in Young Women in South Africa - Study Design and Baseline Results | Wrong population: schoolgirls |
| 124 | Pronyk et al 2006 | Effect of a structural intervention for the prevention of intimate-partner violence and HIV in rural South Africa: a cluster randomised controlled trial | Wrong population: women too old (41 years) |
| 125 | Proscovia et al 2019 | Assessing the impact of an asset-based intervention on educational outcomes of orphaned children and adolescents: findings from a randomised experiment in Uganda | Wrong population: too young (12.7 years); in school |
| 126 | Robertson 2012 | Household-based cash transfer targeting strategies in Zimbabwe: Are we reaching the most vulnerable children? | Excluded publication type: Methods paper |
| 127 | Robertson 2013 | Effects of unconditional and conditional cash transfers on child health and development in Zimbabwe: A cluster-randomised trial | Wrong population: too young; no gender stratification |
| 128 | Rosenberg et al 2014 | The Impact of a National Poverty Reduction Program on the Characteristics of Sex Partners Among Kenyan Adolescents | Not intervention of interest (UCT) |
| 129 | Rotheram-Borus 2012 | Vocational training with HIV prevention for Ugandan youth | Data issues: no gender stratification |
| 130 | Rotheram-Borus 2016 | Feasibility of Using Soccer and Job Training to Prevent Drug Abuse and HIV | Wrong population: young men |
| 131 | Sampa et al 2018 | Effect of cash transfer on school dropout rates using longitudinal data modelling: A randomized trial of research initiative to support the empowerment of girls (RISE) in Zambia | Wrong population: schoolgirls |
| 132 | Sarnquist et al 2014 | Rape Prevention Through Empowerment of Adolescent Girls | Not intervention of interest |
| 133 | Shangani 2017 | Unconditional government cash transfers in support of orphaned and vulnerable adolescents in western Kenya: Is there an association with psychological wellbeing? | Not intervention of interest (UCT) |
| 134 | Sherr et al 2017 | Can cash break the cycle of educational risks for young children in high HIV-affected communities? A cross-sect study in SA and Malawi | Not intervention of interest (UCT); Wrong population: too young (10.21 years) |
| 135 | Shimamura et al 2010 | Credit Program Participation and Child Schooling in Rural Malawi | Wrong population: heads of households (46 years) |
| 136 | Sinclair et al 2013 | A Self-Defence Program Reduces the Incidence of Sexual Assault in Kenyan Adolescent Girls | Not intervention of interest |
| 137 | Sitienei, Pillay 2019 | Psycho-education and social interventions provided for orphans and vulnerable children at a community-based organisation in Soweto, South Africa | Data issues: no gender-stratified data; no HIV-outcomes |
| 138 | Skovdal 2010 | Community relations and child-led microfinance: A case study of caregiving children in Kenya | Wrong population: too young |
| 139 | Skovdal et al 2013 | Social acceptability and perceived impact of a community-led cash transfer programme in Zimbabwe | Wrong population: caregiver of kids |
| 140 | Skovdal et al 2013 | The impact of community-based capital cash transfers on orphan schooling in Kenya | Wrong population: no dropouts |
| 141 | Smith et al 2019 | Cash transfers and HIV/HSV-2 prevalence: A replication of a cluster randomized trial in Malawi | Wrong population: schoolgirls + wrong intervention: return to school (replication Baird 2012 trial) |
| 142 | Ssewamala et al 2009 | Integrating Children's Savings Accounts in the Care and Support of Orphaned Adolescents in Rural Uganda | Wrong population: too young and in school |
| 143 | Ssewamala et al 2009 | Asset ownership and health and mental health functioning among AIDS-orphaned adolescents: Findings from a randomized controlled trial in rural Uganda | Wrong population: too young and in school |
| 144 | Ssewamala et al 2010 | Effect of Economic Assets on Sexual Risk-Taking Intentions Among Orphaned Adolescents in Uganda | Wrong population: too young and in school |
| 145 | Ssewamala et al 2010 | Gender and the Effects of an Econ Empowerment Program on Attitudes Toward Sex Risk-Taking Among AIDS-Orphaned Ado Youth in Uganda | Wrong population: too young and in school |
| 146 | Ssewamala et al 2012 | The Impact of a Comprehensive Microfinance Intervention on Depression Levels of AIDS-Orphaned Children in Uganda | Wrong population: too young and in school |
| 147 | Ssewamala et al 2014 | Integrating a Mentorship Component in Programming for Care and Support of AIDS-Orphaned and Vulnerable Children: Lessons from the Suubi and Bridges Programs in Sub-Saharan Africa | Wrong population: too young and in school |
| 148 | Ssewamala et al 2016 | Applying a Family-Level Economic Strengthening Intervention to Improve Education and health-related Outcomes of School-Going AIDS-Orphaned Children: Lessons from randomized experiment in Southern Uganda | Wrong population: too young and in school |
| 149 | Ssewamala et al 2018 | Cost-Effectiveness of a Savings-Led Econ Empowerment Intervention for AIDS-Affected Adolescents in Uganda: Implications for Scale-up in Low-Resource Communities | Wrong population: too young and in school |
| 150 | Stoner et al 2019 | The effects of a cash transfer intervention on sexual partnerships and HIV in the HPTN 068 study in South Africa | Not intervention of interest |
| 151 | Thwala 2008 | Skills development in South Africa: Group Five's social investment project | Data issues: no gender stratification |
| 152 | Toska et al 2017 | School, Supervision and Ado-Sens Clinic Care: Combination SP and Reduced Unprotected Sex Among HIV-Positive Adolescents in South Africa | Not intervention of interest |
| 153 | Tshishonga 2016 | Women growing livelihoods through food security: Inanda's Inqolobane Yobumbano Secondary Co-operative | Wrong population: older women |
| 154 | Visser 2005 | Life skills training as HIV/AIDS preventive strategy in secondary schools: Evaluation of a large-scale implementation process | Not intervention of interest |
| 155 | Vyas et al 2015 | Exploring the association between women's access to economic resources and intimate partner violence in Dar es Salaam and Mbeya, Tanzania | Data issues: no age stratification |
| 156 | Wang et al 2018 | Effects of Financial Incentives on Saving Outcomes and Material Well-Being: Evidence From a RCT in Uganda | Wrong population: too young and in school |
| 157 | Weinhardt 2017 | Effects of Financial Incentives on Saving Outcomes and Material Well-Being: Evidence From a RCT in Uganda | Wrong population: women too old (40 years) |
| 158 | Weiser et al 2015 | Shamba Maisha: Randomized controlled trial of an agricultural and finance intervention to improve HIV health outcomes | Wrong population: women too old (37 years); no gender disaggregation |
| 159 | Zulu et al 2018 | Community based intervention for strengthening ado sex reproductive H and rights: how can they be integrated and sustained? A realist evaluation protocol from Zambia | Excluded publication type: Protocol |
|  | **Grey literature** |  |  |
| 160 | Adoho et al 2014 | the impact of an AG employment program: the EPAG project in Liberia | Wrong country: Liberia |
| 161 | Brudewold-Newman 2017 | A firm one’s own | No HIV-related outcomes |
| 162 | Buehren 2016 | Women empowerment, sibling rivalry, and competitiveness: evidence from a lab experiment and a randomized controlled trial in Uganda | Not intervention of interest |
| 163 | BOMA project 2018 | A longitudinal Assessment of REAP: a follow up on program participants 3-5 years after graduation | No HIV-related outcomes |
| 164 | Cho et al 2013 | Gender differences in the effects of vocational training – constraints on women and dropout behaviour | No HIV-related outcomes |
| 165 | Devereux 2016 | Accessing the right kinds of material and symbolic capital: role of cash transfers in reducing adolescent school absence and risky behaviour in South Africa | Not intervention of interest |
| 166 | Dietrich 2016 | Women’s opportunities and challenges in sub-Saharan Africa Job markets- IMF working paper | Not intervention of interest |
| 167 | Hallman 2007 | Enhancing Financial Literacy, HIV/AIDS Skills, and Safe Social Spaces among Vulnerable South African youth | Data issues: no data stratification possible between in/out-of-school boys/girls |
| 168 | Innocenti Res Centre/UNICEF 2018 | A Cash Plus model for safe transitions to a healthy and productive adulthood: baseline report | Data issues: only baseline results |
| 169 | Lukas 2008 | Reducing adolescent girls’ vulnerability to HIV infection: examining microfinance and sustainable livelihood approaches | Excluded publication type: Review |
| 170 | Marcus 2017 | GAGA (Gender & Adolescence: Global Evidence) Rigorous Review; Girls’ clubs, life skills programmes and girls’ wellbeing outcomes | Excluded publication type: Review |
| 171 | Palermo 2018 | Tanzania Youth study of the productive social safety net (PSNN) Impact evaluation | Data issues: no results stratification for public works component; no gender stratification |
| 172 | Transfer project 2018 | Ujana Salama: Cash plus model on youth well-being and safe, healthy transitions | Excluded publication type: not an empirical study. This is the summary of the Innocent 2018 publication |
|  |  |  |  |

Abbreviations: CCT: conditional cash transfer; IPV: intimate partner violence; UCT: unconditional cash transfer.

**Additional File 5. MMAT Quality Appraisal Results**

Hong QN, Fàbregues S, Bartlett G, Boardman F, Cargo M, Dagenais P, et al. The Mixed Methods Appraisal Tool (MMAT) 2018. Registration of Copyright (#1148552), Canadian Intellectual Property Office, Industry Canada.

**Austrian (2015): *Barriers and facilitators to health behaviour change and economic activity among slum dwelling adolescent girls and young women in Nairobi, Kenya: the role of social, health and economic assets - high***

| **Type study** | **Meth. Quality criteria** | **yes** | **no** | **Can’t tell** | **Comments** |
| --- | --- | --- | --- | --- | --- |
| 1. Qualitative | 1.1. Is the qualitative approach appropriate to answer the research question? | x |  |  | Qualitative description: in-depth interview (IDI), focus group discussion (FGD), hybrid thematic analysis. To explore barriers/facilitators health behaviour change and understand how different assets support transition into economic activity. |
|  | 1.2. Are the qualitative data collection methods adequate to address the research question? | x |  |  | Data sources: 128 young women aged 18-25 years; 63 IDI; 11 FGD with 65 participants (2 Binti; 5 vocational training; 4 no programme participation). Experienced peer interviewers. FGD and IDI guides written, translated into Swahili; field-tested with the three different types of girls. Recording, translation & transcription by interviewers. |
|  | 1.3. Are the findings adequately derived from the data? | x |  |  | Coding, reconciliation & analysis by two study staff. Thematic analysis: themes regrouped as barrier or facilitator for both health behaviour change and transition into economic activity. |
|  | 1.4. Is the interpretation of results sufficiently substantiated by data? | x |  |  | Every theme is supported by 1-3 relevant quotes. |
|  | 1.5. Is there coherence between qualitative data sources, collection, analysis and interpretation? | x |  |  | Good coherence. Discussion key barriers/facilitators; interaction of economic, human and social assets and their potential to strengthen effects when AGYW possess all three. Programme recommendations. |

**Austrian (2020): The impact of the Adolescent Girls Empowerment Program (AGEP) on short and long term social, economic, education and fertility outcomes: a randomized controlled trial in Zambia *- high***

| **Type study** | **Meth. Quality criteria** | **yes** | **no** | **Can’t tell** | **Comments** |
| --- | --- | --- | --- | --- | --- |
| 2. Quantitative  RCT | 2.1. Is randomization appropriately performed? | x |  |  | Stratified (urban/rural) randomization of sites. Random selection intervention clusters and assignment study arms through public lottery. From vulnerability list, most vulnerable AGYW randomly selected. |
|  | 2.2. Are the groups comparable at baseline? | x |  |  | Baseline balanced although differential attrition in analytical sample (excluded girls: older, less likely in school/living with mom; lower grades) |
|  | 2.3. Are there complete outcome data? | x |  |  | Retention at endline was 88%; 2 years after endline 66% of baseline sample); balanced across treatment and control groups. |
|  | 2.4. Are outcome assessors blinded to the intervention provided? |  | x |  | Author replied that due to nature of the intervention it was impossible to blind data collectors to random assignment |
|  | 2.5 Did the participants adhere to the assigned intervention? | In part |  |  | 25% did not participate at all; 30% participated in half the program sessions. Analysis used ITT and TOT so we can see the impact of the intervention among those who were meant to complete the intervention and those that actually did. Adherence for attendance of meetings was defined as having attended at least half of the weekly meetings. |

**Bandiera (2015): *Women’s economic empowerment in action: Evidence from a randomized control trial in Africa– moderate***

| **Type study** | **Meth. Quality criteria** | **yes** | **no** | **Can’t tell** | **Comments** |
| --- | --- | --- | --- | --- | --- |
| 2. Quantitative  RCT | 2.1. Is randomization appropriately performed? |  |  | x | Cluster randomization: In each of 5 BRAC branches, 15 potential communities identified. From this list, communities were randomly assigned to intervention (10) and control (5) = total communities = 100.  Based on 2008 census, average of 130 eligible girls/community identified, of which a random sample of 40 girls was drawn. The process of randomization is not described. |
|  | 2.2. Are the groups comparable at baseline? | x |  |  | OLS regression, followed by computation of normalized difference show that groups are balanced at baseline (Table 1). |
|  | 2.3. Are there complete outcome data? | x |  |  | 82% retention after 2 years (baseline: n=5,966 & FU n=4,888). |
|  | 2.4. Are outcome assessors blinded to the intervention provided? |  |  | x | This is not reported. Assessors were not implementers. |
|  | 2.5 Did the participants adhere to the assigned intervention? | In part |  |  | Only 21% intervention girls participated (club relies on voluntary participation) but did so intensely: 63% for 2-year duration & 77% once or twice per week or more. Participants do not significantly differ from non-participants (on demographic characteristics and outcome variables); 4.7% control girls participated, but do not continue for long (75% dropped out 6 months prior midline –Bandiera 2018).  Given voluntary participation: estimation based on both ITT and TOT |

**Bandiera (2018): *Women’s empowerment in action: Evidence from a randomized control trial in Africa – moderate***

| **Type study** | **Meth. Quality criteria** | **yes** | **no** | **Can’t tell** | **Comments** |
| --- | --- | --- | --- | --- | --- |
| 2. Quantitative  RCT | 2.1. Is randomization appropriately performed? |  |  | x | See Bandiera 2015 |
|  | 2.2. Are the groups comparable at baseline? | x |  |  | See Bandiera 2015 |
|  | 2.3. Are there complete outcome data? | x |  |  | 65% retention after 4 years (authors attempted to track all girls from baseline). |
|  | 2.4. Are outcome assessors blinded to the intervention provided? |  |  | x | Not reported. |
|  | 2.5 Did the participants adhere to the assigned intervention? | In part |  |  | Item less relevant as this follow up study explores sustainability of effects. See Bandiera 2015: after 2 years girls were allowed to continue using clubs as safe space, but training was not continued. |

**Buehren (2017): *Evaluation of an adolescent development program for girls in Tanzania – low***

| **Type study** | **Meth. Quality criteria** | **yes** | **no** | **Can’t tell** | **Comments** |
| --- | --- | --- | --- | --- | --- |
| 2. Quantitative  RCT | 2.1. Is randomization appropriately performed? |  |  | x | Multi-level cluster randomization: (1) In each of 10 BRAC branches, 15 potential communities identified. From this list communities were randomly assigned to intervention (100) and control (50); (2) Treatment communities were further divided into 3 blocks: (A) Club only; (B) Club + MFI; (C) Control. Randomization description could be more detailed. |
|  | 2.2. Are the groups comparable at baseline? | x |  |  | Table A1: normalized difference between 2 Rx groups and control <0.1; significant in only 1/40 cases (HIV knowledge in Club only girls). |
|  | 2.3. Are there complete outcome data? |  | x |  | Attrition is 42%: girls enrolled in schools and those with children at baseline more likely retained. Estimated coefficients on interactions of baseline characteristics with treatment dummies show they are jointly not significant and programme dropouts are not statistically different from those retained. Robustness checks help account for attrition. |
|  | 2.4. Are outcome assessors blinded to the intervention provided? |  |  | x | Is not reported |
|  | 2.5 Did the participants adhere to the assigned intervention? |  | x |  | Overall participation rates lower than in Uganda (21%), but participation is voluntary. Tanzania: 19% Club+MFI, which is significantly higher than 13% Club only & 7% control. Although participation in all programme activities increased 6% after introduction MFI, only 4% participated in microfinance activities. – They don’t report how many sessions they attended. |

**Burke (2019): *Quasi-experimental evaluation using confirmatory procedures: A case study of economic and social empowerment intervention to reduce girls' vulnerability to HIV in rural Mozambique – moderate***

| **Type study** | **Meth. Quality criteria** | **yes** | **no** | **Can’t tell** | **Comments** |
| --- | --- | --- | --- | --- | --- |
| 3. Quantitative non randomized | 3.1. Are the participants representative of the target population? | x |  |  | Purposive sampling of intervention communities and participants aged 13-19 years; vulnerable girls having lost at least one parent, living in child-headed household, engaged in transactional sex or other HIV risk behaviours; lacking knowledge re HIV transmission, GBV and unwilling to report GBV/lack access to HIV prevention services. |
|  | 3.2. Are measurements appropriate regarding both the outcome and intervention (or exposure)? | x |  |  | In theory, there were detailed descriptions of creation of composite HIV & GBV knowledge. In practice, it was difficult to collect accurate data (e.g., lying about age to be eligible to participate.) |
|  | 3.3. Are there complete outcome data? |  | x |  | 87% retention in the intervention, but during analysis, 59/233 (25% intervention group) and 266/589 (45% control group) girls were dropped, as they lacked exact match. |
|  | 3.4. Are the confounders accounted for in the design and analysis? | x |  |  | Through multilevel exact matching, stratification, and dropping of outliers/cases with missing values/lack of exact match, individual and community confounders were accounted for. |
|  | 3.5. During the study period, is the intervention administered (or exposure occurred) as intended? |  |  | x | The authors report some variability in implementation by community. |

**Burke (2019): *A longitudinal qualitative evaluation of economic and social empowerment intervention to reduce girls' vulnerability to HIV in rural Mozambique - high***

| **Type study** | **Meth. Quality criteria** | **yes** | **no** | **Can’t tell** | **Comments** |
| --- | --- | --- | --- | --- | --- |
| 1. Qualitative | 1.1. Is the qualitative approach appropriate to answer the research question? | x |  |  | Qualitative description: IDI, FGD, hybrid thematic analysis. To understand /explore multi-faceted, interacting effects of personal & environmental factors that determine behaviour/ leverage health promotion using the Social Ecological Model. |
|  | 1.2. Are the qualitative data collection methods adequate to address the research question? | x |  |  | Data sources: IDIs with 49 girls, 36 influential males, 24 heads of households; 12 FGD with 6-11 community members: government, local leaders, Women First staff. Trained interviewers conducted two rounds of gender matched IDIs and FGDs in local languages. Semi-structured interview guides; audio recording; translation/transcription to Portuguese by interviewers; professional Portuguese-English translation. |
|  | 1.3. Are the findings adequately derived from the data? | x |  |  | Development of initial codebook for each respondent type; iterative coding by 3 analysts; use of software; intercoder-reliability; coding reports and summarized data at overarching theme/SEM levels. |
|  | 1.4. Is the interpretation of results sufficiently substantiated by data? | x |  |  | Detailed reporting of findings per theme, each supported by 1-9 quotes, mostly from girls, a few from FGDs, only one from an influential male; none from heads of households. |
|  | 1.5. Is there coherence between qualitative data sources, collection, analysis and interpretation? | x |  |  | Good coherence. Unintended consequences adequately addressed in recommendations. Strengths and limitations addressed. |

**Dunbar (2010): *Findings from SHAZ!: a feasibility study of a microcredit & life-skills HIV prevention intervention to reduce risk among adolescent female orphans in Zimbabwe –moderate***

| **Type study** | **Meth. Quality criteria** | **yes** | **no** | **Can’t tell** | **Comments - *moderate*** |
| --- | --- | --- | --- | --- | --- |
| 1. Qualitative | 1.1. Is the qualitative approach appropriate to answer the research question? | x |  |  | To explore barriers and refine programme. |
|  | 1.2. Are the qualitative data collection methods adequate to address the research question? | In part |  |  | Qualitative description using semi-structured, open-ended interviews with 13 loan recipients. Paper mentions there are FGD post-intervention to improve life-skills sessions but does not describe processes to ensure data quality and instrumental reliability. |
|  | 1.3. Are the findings adequately derived from the data? | x |  |  | Data analysis relied on rapid content analysis and priority ranking; mentorship component analysed information from monitoring reports. |
|  | 1.4. Is the interpretation of results sufficiently substantiated by data? | In part |  |  | Two out of three themes well supported by relevant quotes; No quotes reported for the mentorship theme; not clear whether monitoring reports were suitable to derive quotes from. |
|  | 1.5. Is there coherence between qualitative data sources, collection, analysis and interpretation? | x |  |  | Good coherence. Unintended consequences of the study addressed in the discussion and authors described their efforts and recommendations to mitigate these. |
| **Type study** | **Meth. Quality criteria** | **yes** | **no** | **Can’t tell** | **Comments - *moderate*** |
| 4. Quantitative descriptive | 4.1. Is the sampling strategy relevant to address the research question? | x |  |  | Non-probability sampling of a subset of 50 participants recruited from a cross-sectional survey of 200 (non)orphan girls aged 16-19 years (Kang 2008). Two groups of 25 girls, which is the group size required to pilot the life-skills curriculum. Sampling procedure is not described. |
|  | 4.2. Is the sample representative of the target population? | x |  |  | Eligibility criteria: girls aged 16-19 years being orphaned and out of school in Chitungwiza & Epworth, (peri-)urban communities near Harare. |
|  | 4.3. Are the measurements appropriate? | In part |  |  | Most variables appropriate and some clearly defined. Use of validated sexual relationship power scores (Dunkle 2004; Pulewitz 2000) and clinical tests for biological outcomes (HIV; HSV2; pregnancy tests). Not enough detail on measures for sexual behaviour and economic outcomes |
|  | 4.4. Is risk of nonresponse bias low? |  | x |  | Baseline n=49; 6-month follow up n=37 (Table 2). Attrition rate is 24%, which seems quite high for small n and short time period. Paper does not report reasons for no-response or whether non-respondents were different. This is surprising given stated aim included identifying methods for retention. |
|  | 4.5. is the statistical analysis appropriate to answer research the question? | x |  |  | Pre-post descriptive study compared baseline and 6-months survey data using Chi square or Fischer exact tests for significance. Small sample size precludes assessing outcome data. |
| **Type study** | **Meth. Quality criteria** | **yes** | **no** | **Can’t tell** | **Comments *- low*** |
| 5. Mixed methods | 5.1. Is there an adequate rationale for using a mixed methods design to address the research question? |  | x |  | No explicit justification for use of mixed methods |
|  | 5.2. Are the different components of the study effectively integrated to answer the research question? |  | x |  | Qualitative and quantitative findings are presented separately but not combined. |
|  | 5.3. Are the outputs of the integration of qualitative and quantitative components adequately interpreted? |  | x |  | Outputs were not integrated |
|  | 5.4. Are divergences and inconsistencies between quantitative and qualitative results adequately addressed? |  |  |  | n/a qualitative and quantitative components addressed different research questions. |
|  | 5.5. Do the different components of the study adhere to the quality criteria of each tradition of the methods involved? | x |  |  |  |

**Dunbar (2014): *The SHAZ! Project: results from a pilot randomized trial of a structural intervention to prevent HIV among adolescent women in Zimbabwe – moderate***

| **Type study** | **Meth. Quality criteria** | **yes** | **no** | **Can’t tell** | **Comments** |
| --- | --- | --- | --- | --- | --- |
| 2. Quantitative  RCT | 2.1. Is randomization appropriately performed? | x |  |  | Randomized with computer, then opaque envelopes. Out-of-school, not currently pregnant, HIV negative adolescent female orphans aged 16-19 years living in Chitungwiza. |
|  | 2.2. Are the groups comparable at baseline? | In part |  |  | Yes, except for completion secondary school: 80% intervention participants vs 70% control completed secondary school (p=0.03) |
|  | 2.3. Are there complete outcome data? | x |  |  | 19% attrition for 2-year study: 60/315 (26 Rx vs 34 control) discontinued from study for known reasons n=24 (death, relocation, return to education, partner influence); unknown reasons n=36 |
|  | 2.4. Are outcome assessors blinded to the intervention provided? |  |  | x | Is not reported. |
|  | 2.5 Did the participants adhere to the assigned intervention? | In part |  |  | Overall training completion was 82%. No statistical difference for completing life skills (94%) or Red Cross training (77%); 70% initiated vocational training (63% passed) and less than 60% received micro grant. |

**Dunbar (2017): *Empowering adolescent girls and women for improved sexual health in Zimbabwe: lessons learned from a combined livelihoods and life skills intervention (SHAZ!) - low***

| **Type study** | **Meth. Quality criteria** | **yes** | **no** | **Can’t tell** | **Comments** |
| --- | --- | --- | --- | --- | --- |
| 1. Qualitative | 1.1. Is the qualitative approach appropriate to answer the research question? | x |  |  | Case study aiming to explore and understand differences between treatment and control groups |
|  | 1.2. Are the qualitative data collection methods adequate to address the research question? |  |  | x | Paper refers to data from 2010 study (semi-structured interviews with 13 loan recipients & FGD post-intervention -lacks description methods) along with qualitative programme monitoring data and guidance counselling visit notes. |
|  | 1.3. Are the findings adequately derived from the data? |  |  | x | Analysis consisted of reviewing these documents for common themes and issues. No mention of coding process, within/cross-case analysis. |
|  | 1.4. Is the interpretation of results sufficiently substantiated by data? |  | x |  | There is a single theme –no quote |
|  | 1.5. Is there coherence between qualitative data sources, collection, analysis and interpretation? |  |  | x |  |

**Erulkar (2005): *Evaluation of a savings & micro-credit program for vulnerable young women in Nairobi – moderate***

| **Type study** | **Meth. Quality criteria** | **yes** | **no** | **Can’t tell** | **Comments** |
| --- | --- | --- | --- | --- | --- |
| 3. Quantitative non randomized | 3.1. Are the participants representative of the target population? |  | x |  | Out of school AGYW 16-22 years old in urban slum areas in Nairobi  Pilot n=100 (16-24 years); Intervention: 326 baseline, 222 endline.  AGYW self-selected into programme, 70% heard about TRY from friends and only 6% from credit officers. |
|  | 3.2. Are measurements appropriate regarding both the outcome and intervention (or exposure)? | x |  |  | Relevant variables: demographic details, family info, household conditions/assets, education, time use, mobility, group participation, gender attitudes, savings, (un)paid work, sexual behaviour and sexual violence).  Process data: monthly performance records with numbers and rates for participation, (cumulative amount of) savings, loan disbursement, repayment and dropouts. |
|  | 3.3. Are there complete outcome data? |  | x |  | Response rate of 68%; among controls it was 17%. NB: all girls lost to follow-up were dropouts. |
|  | 3.4. Are the confounders accounted for in the design and analysis? | x |  |  | Each participant was matched to control for age, education, marital status, parenthood, employment and neighbourhood (SES). Controls identified through house-so-house survey. |
|  | 3.5. During the study period, is the intervention administered (or exposure occurred) as intended? | In part |  |  | The project (2001-04) was adapted several times, which likely affected outcomes. 90% exposed to training, savings, mentors; 54% to MFI; at endline 66% had dropped out of programme. |

**Erulkar (2006): *Tap and Reposition Youth (TRY): Providing social support, savings, and microcredit opportunities for young women in areas with high HIV prevalence – low***

| **Type study** | **Meth. Quality criteria** | **yes** | **no** | **Can’t tell** | **Comments** |
| --- | --- | --- | --- | --- | --- |
| 1. Qualitative | 1.1. Is the qualitative approach appropriate to answer the research question? | x |  |  | Case study: in-depth exploration & explanation of TRY intervention |
|  | 1.2. Are the qualitative data collection methods adequate to address the research question? |  |  | x | Data collection methods are not described in this report. |
|  | 1.3. Are the findings adequately derived from the data? |  |  | x | Data analysis is not described in this report. |
|  | 1.4. Is the interpretation of results sufficiently substantiated by data? | x |  |  | Findings related to girls’ experiences are supported by 2-11 relevant quotes per them (social support in group meetings, safe space for savings, linking vulnerability & HIV risk, microfinance, new cadre of mentors, personal savings, young savers club). |
|  | 1.5. Is there coherence between qualitative data sources, collection, analysis and interpretation? |  |  | x | Data collection methods and analysis are not described. This report contributes valuable in-depth process descriptions explaining how and why the TRY intervention evolved over time. It likely targets a practice rather than research audience. It should therefore be read in conjunction with the 2005 report that reports on methodology. |

**Gibbs (2020): *Stepping Stones and Creating Futures Interventions to prevent intimate partner violence among young people: Cluster Randomized Controlled Trial – high***

| **Type study** | **Meth. Quality criteria** | **yes** | **no** | **Can’t tell** | **Comments** |
| --- | --- | --- | --- | --- | --- |
| 2. Quantitative  RCT | 2.1. Is randomization appropriately performed? | x |  |  | Use of Excel random number generator for cluster allocation prior to participant recruitment (n=34 clusters) |
|  | 2.2. Are the groups comparable at baseline? | x |  |  | Table 2: similar group sizes and primary and secondary outcomes were similar across arms |
|  | 2.3. Are there complete outcome data? | x |  |  | At 12 months retention for women was 64.3% (is low) but at 24 months it was 80.5% |
|  | 2.4. Are outcome assessors blinded to the intervention provided? |  | x |  | Intervention provider, participants, and statistician were not blinded to arm allocation. |
|  | 2.5 Did the participants adhere to the assigned intervention? | x |  |  | 71.1% women attended 3 or more sessions.  NB a total of 38.6% reported also having attended similar workshops (DREAMS) but there were no significant differences by arm. |

**Goodman (2015): *Sexual behavior among young carers in context of a Kenyan empowerment program combining cash-transfer, psychosocial support, and entrepreneurship - high***

| **Type study** | **Meth. Quality criteria** | **yes** | **no** | **Can’t tell** | **Comments** |
| --- | --- | --- | --- | --- | --- |
| 3. Quantitative non randomized | 3.1. Are the participants representative of the target population? | x |  |  | Systematic & purposive sampling of OVC households into intervention by local leaders and social workers.  Inclusion criteria: orphan status with eldest sibling having caring responsibilities, food security, household economic strength at baseline. |
|  | 3.2. Are measurements appropriate regarding both the outcome and intervention (or exposure)? | x |  |  | Outcome measures clearly defined: sexual initiation, unprotected sex in previous six months, and multiple sex partners in the previous year.  Appropriate predictor variables: time in programme; age; orphan household factors; food security & consumption; psychological assets; education; material inputs programme (cash & kits) & personal monthly income. |
|  | 3.3. Are there complete outcome data? | x |  |  | Only 2/1062 programme participants abstained from study. Every February new cohort starts; study compares outcomes between 3 different cohorts who had 0, 1 & 2 years of programme exposure. |
|  | 3.4. Are the confounders accounted for in the design and analysis? | x |  |  | Stratification by gender; use of 2 hierarchical logit models; calculation random intercepts for region/working group & model diagnostics (likelihood ratio test & Global Wald test).  Model nr 2: used all potential covariates and worked backwards using stepwise regression modelling, ascertained coefficients with parallel analyses; nr 1 controls for age & years since most recent parental death. |
|  | 3.5. During the study period, is the intervention administered (or exposure occurred) as intended? | x |  |  | Exposure to sexual behaviour change: ABC training+ VCT similar across groups, but economic intervention used a participatory approach whereby working groups decided themselves on entrepreneurial endeavours, training, loans, revolving funds, emergency cash, skill transfer or starting kits. So, intervention administered as intended but exposure to different programme elements not uniform. |

**Green (2015): *Women's entrepreneurship and intimate partner violence: A cluster randomized trial of microenterprise assistance and partner participation in post-conflict Uganda - high***

| **Type study** | **Meth. Quality criteria** | **yes** | **no** | **Can’t tell** | **Comments** |
| --- | --- | --- | --- | --- | --- |
| 2. Quantitative  RCT | 2.1. Is randomization appropriately performed? | x |  |  | Population of purposively selected 10-17 ultra-poor women per village (no self-selection) in 120 villages; 6 sub-counties (60:60). Allocation first experiment through public lottery; second experiment via computer algorithm. |
|  | 2.2. Are the groups comparable at baseline? | x |  |  | Group sizes similar. Phase I: moderate imbalance: immediate treatment group slightly worse off economically, which would lead to an underestimation of Rx effects; Phase 2: little imbalance. Control for covariates to account for potential bias. |
|  | 2.3. Are there complete outcome data? | x |  |  | At the end of both Phases I & II, 96% retention. |
|  | 2.4. Are outcome assessors blinded to the intervention provided? |  | x |  | Participants and survey enumerators were not blinded. |
|  | 2.5 Did the participants adhere to the assigned intervention? | x |  |  | Phase I: 96% participation; Phase II: 87% programme compliance: 100% women complied, so non-compliance due to male partner non-attendance. |

**Jewkes (2014): *Stepping Stones and Creating Futures intervention: shortened interrupted time series evaluation of a behavioural and structural health promotion and violence prevention intervention for young people in informal settlements in Durban, South Africa – high***

| **Type study** | **Meth. Quality criteria** | **yes** | **no** | **Can’t tell** | **Comments** |
| --- | --- | --- | --- | --- | --- |
| 3. Quantitative non randomized | 3.1. Are the participants representative of the target population? | x |  |  | NGO with local knowledge used flyers and snowball sampling.  Inclusion: out-of-school youth (18-34 years; most under 30 years). |
|  | 3.2. Are measurements appropriate regarding both the outcome and intervention (or exposure)? | x |  |  | Appropriate variables were clearly defined and measured: items in self-completed questionnaires used validated, standard scales. The authors assessed demographic and socio-economic variables, sexual behaviour, gender attitudes, mental health, and participation in crime |
|  | 3.3. Are there complete outcome data? | x |  |  | Retention is 90.2% among women. |
|  | 3.4. Are the confounders accounted for in the design and analysis? | x |  |  | Analysis was by intention to treat, irrespective of attendance. |
|  | 3.5. During the study period, is the intervention administered (or exposure occurred) as intended? | In part |  |  | Estimated attendance rate among those who attended was only 60% but this did not affect outcome as analysis was ITT. |

**Pettifor (2019): *Cash plus: exploring mechanisms through which a cash transfer plus financial education programme in Tanzania reduced HIV risk for adolescent girls and young women - high***

| **Type study** | **Meth. Quality criteria** | **yes** | **no** | **Can’t tell** | **Comments** |
| --- | --- | --- | --- | --- | --- |
| 1. Qualitative | 1.1. Is the qualitative approach appropriate to answer the research question? | x |  |  | Qualitative description: 60 IDI; 20 narrative timeline interviews, inductive thematic analysis. To explore perceived effects combined cash transfer, behaviour change communication and microfinance programme on risky sex. |
|  | 1.2. Are the qualitative data collection methods adequate to address the research question? | x |  |  | Out-of-school AGYW aged 15-23 years: 20 baseline and 20 FU interviews; 40 new IDI & 20 narrative timeline interviews. Experienced female research assistants; interview guide piloted; interviews in local language, audio-recorded, transcribed, and checked for quality. |
|  | 1.3. Are the findings adequately derived from the data? | x |  |  | Applied thematic analysis supported by software. Multiple coders, measures to ensure coding consistency. Emergent themes discussed among 4 researchers. Matrix-supported comparison of thematic summaries across interviews. |
|  | 1.4. Is the interpretation of results sufficiently substantiated by data? | x |  |  | Every theme is supported by 1-3 relevant quotes. Findings also visually supported by a conceptual framework using observed and hypothetical pathways. |
|  | 1.5. Is there coherence between qualitative data sources, collection, analysis and interpretation? | x |  |  | Good coherence. Discussion highlights different pathways through which the intervention may have reduced HIV risk. Relevant recommendations. Limitations addressed. |

**Pronyk (2008): *A combined microfinance and training intervention can reduce HIV risk behaviour in young female participants – high***

| **Type study** | **Meth. Quality criteria** | **yes** | **no** | **Can’t tell** | **Comments *– high*** |
| --- | --- | --- | --- | --- | --- |
| 1. Qualitative | 1.1. Is the qualitative approach appropriate to answer the research question? |  |  | x | There is no specific qualitative research question, but it can be derived from statement to contextualize results –would answer the ‘how’ |
|  | 1.2. Are the qualitative data collection methods adequate to address the research question? | x |  |  | 8 key informant interviews with loan recipients, twice 8 FGDs, non-participant observation (160 women followed 1 year), diaries of IMAGE training facilitators. |
|  | 1.3. Are the findings adequately derived from the data? | x |  |  | All data were translated, transcribed and analysed in Nud*ist software with thematic content analysis. Table 2 shows raw data in categories and overarching themes. |
|  | 1.4. Is the interpretation of results sufficiently substantiated by data? | x |  |  | Table 2 gives overview of raw data rather than these being interspersed in the textual reporting of findings. |
|  | 1.5. Is there coherence between qualitative data sources, collection, analysis and interpretation? | x |  |  | Good coherence. Discussion addresses limitations and provides relevant recommendations on delivery integrated health and development programmes. |
| **Type study** | **Meth. Quality criteria** | **yes** | **no** | **Can’t tell** | **Comments *– high*** |
| 2. Quantitative  RCT | 2.1. Is randomization appropriately performed? | x |  |  | 8 villages pair-matched (accessibility & size); one from each pair randomly selected for Rx. Each young woman in intervention matched for age and poverty with randomly selected woman in control village. Blind randomization described in Pronyk 2006 |
|  | 2.2. Are the groups comparable at baseline? | x |  |  | No significant differences in baseline characteristics. |
|  | 2.3. Are there complete outcome data? | x |  |  | 2-year follow up rates were 92% intervention and 79% for control. |
|  | 2.4. Are outcome assessors blinded to the intervention provided? |  |  | x | Not described whether interviewers were blinded; analysis was blinded to the village of origin (Pronyk 2006) |
|  | 2.5 Did the participants adhere to the assigned intervention? | in  part |  |  | Paper only says that process evaluation data (reported in Pronyk 2006) showed high participation rates; per protocol analysis. |
| **Type study** | **Meth. Quality criteria** | **yes** | **no** | **Can’t tell** | **Comments - *high*** |
| 5. Mixed methods | 5.1. Is there an adequate rationale for using a mixed methods design to address the research question? |  | x |  | No mention of mixed methods, but they justify using qualitative methods. |
|  | 5.2. Are the different components of the study effectively integrated to answer the research question? | x |  |  | Qualitative and quantitative results are presented for different effects. |
|  | 5.3. Are the outputs of the integration of qualitative and quantitative components adequately interpreted? | x |  |  | Authors report that qualitative findings seem to support quantitative results. |
|  | 5.4. Are divergences and inconsistencies between quantitative and qualitative results adequately addressed? |  |  |  | n/a |
|  | 5.5. Do the different components of the study adhere to the quality criteria of each tradition of the methods involved? | x |  |  |  |
